# Supplementary material for: Cell-penetrating protein-recognizing polymeric nanoparticles through dynamic covalent chemistry and double imprinting
Source: Nat Commun. 2024 May 3;15:3731. doi: 10.1038/s41467-024-48131-5 (PMC11068882; doi:10.1038/s41467-024-48131-5)
Supplement: Supplementary file 1 — Supplementary Information [file 41467_2024_48131_MOESM1_ESM.pdf]

Supplementary Information

Cell-Penetrating Protein-Recognizing Polymeric Nanoparticles through Dynamic Covalent Chemistry and Double Imprinting

Avijit Ghosh, Mansi Sharma, and Yan Zhao*

Department of Chemistry, Iowa State University, Ames, Iowa 50011-3111, USA

1. Supplementary Methods

1.1 General Experimental Methods

All reagents and solvents were of ACS-certified grade or higher and used as received from commercial suppliers. Millipore water (18.2 MU; Millipore Co., USA) was used to prepare buffers and nanoparticles. All new compounds were characterized by ^1H NMR, ^{13}C NMR, and HRMS. NMR spectra were recorded on a Bruker DRX-400, a Bruker AV III 600, or a Varian VXR-400 spectrometer. Chemicals shifts are reported in ppm relative to residual solvent peaks. High resolution mass spectra (HRMS) were recorded on Agilent QTOF 6540 mass spectrometer with a QTOF detector. Dynamic light scattering (DLS) was recorded at 25 °C on a Malvern Zetasizer Nano ZS instrument. Isothermal titration calorimetry (ITC) was performed using a MicroCal VP-ITC Microcalorimeter with Origin 7 software and VPViewer2000 (GE Healthcare, Northampton, MA). TEM was analyzed on a 200kV JEOL 2100 electron microscope. Zeiss upright and Zeiss confocal microscope was used for fluorescence cell imaging. ZEN microscopy software was used to analyze the images.

1.2 Syntheses

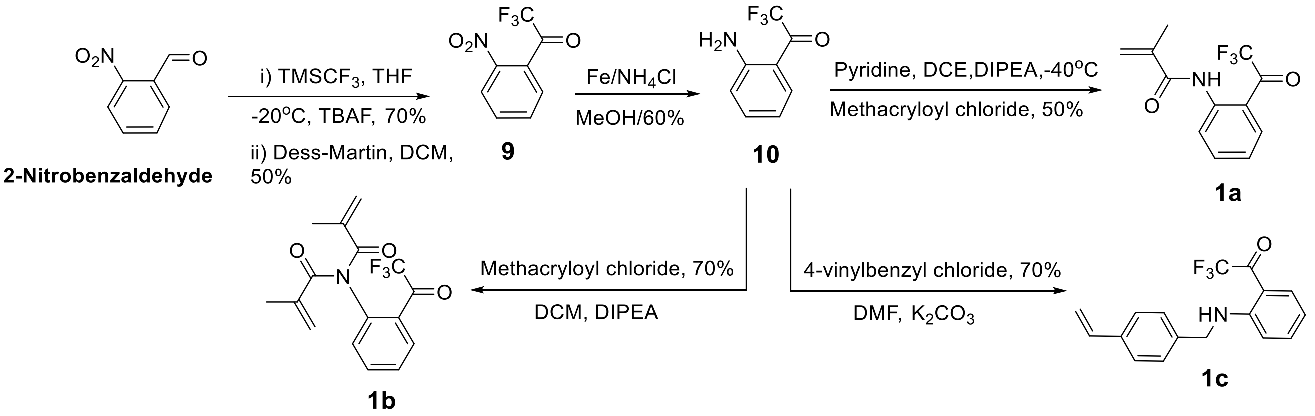

Supplementary Figure 1. Synthesis of compound **1a–c.**

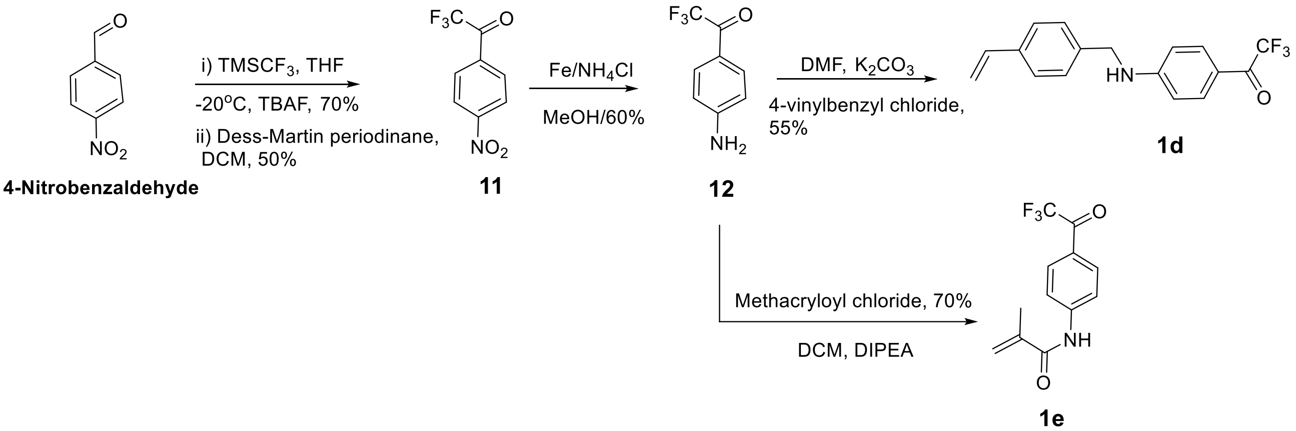

Supplementary Figure 2. Synthesis of compound **1d–e**.

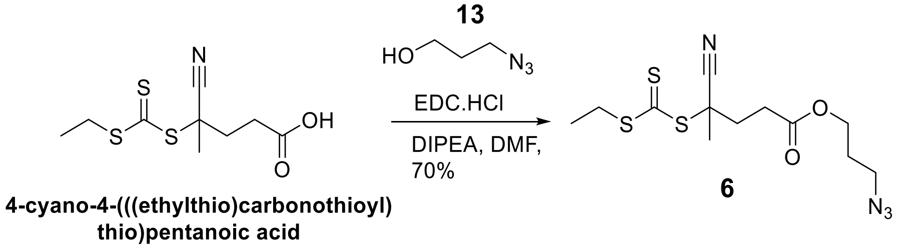

Supplementary Figure 3. Synthesis of compound **6**.

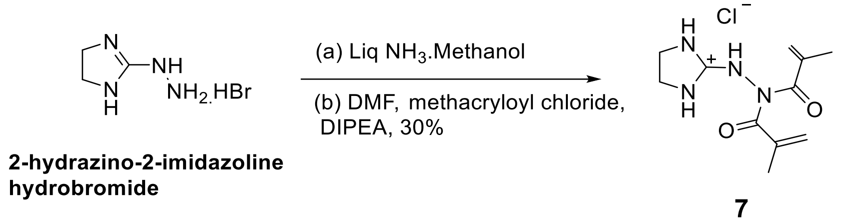

Supplementary Figure 4. Synthesis of compound **7**.

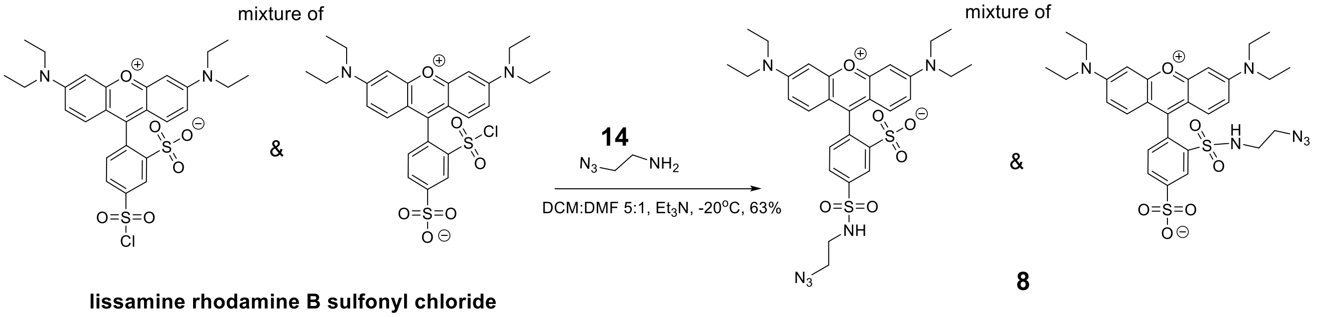

Supplementary Figure 5. Synthesis of compound **8**.

Syntheses of compounds **3**,[1] **4**,[2] **5**,[3] **9–12**,[4,5] **13**,[6] and **14**[1] followed previously reported procedures.

Compound 1a. Methacryloyl chloride (0.047 mL, 0.5 mmol) was slowly added to a stirred solution of compound **10** (190 mg, 1.0 mmol), N, N-diisopropylethylamine (DIPEA, 0.17 mL, 1.0 mmol) and pyridine (0.08 mL, 1.0 mmol) in dichloroethane (10 mL) at -40 °C. After being stirred for 15-20 min, the reaction mixture was diluted with CH_2Cl_2 (5 mL). The combined organic solution was washed with 1 M HCl (10 mL) and water (2 × 10 mL), dried with magnesium sulfate, filtered, and concentrated by rotary evaporation. The residue was purified by column chromatography over silica gel using 5:1 hexane/ethyl acetate as eluent to afford a light-yellow oil (129 mg, 50 %). ^{1}H NMR (600 MHz, $CDCl_3$, δ): 11.51 (s, 1H), 8.98 (d, *J* = 6.0 Hz,1H), 8.03 (m, 1H), 7.76 (m, 1H), 7.24 (m, 1H), 6.08 (s, 1H), 5.64 (s, 1H), 2.17 (s, 3H). ^{13}C NMR (150 MHz, CDCl3, δ): 183.3 (q, *J* = 34.5 Hz), 167.1, 143.8, 140.4, 137.9, 132.1 (q, *J* = 4.5 Hz), 122.8, 122.2, 121.3, 116.7 (q, *J* = 289.5 Hz), 115.5, 18.6. ESI-HRMS (*m/z*): $[M+H]^+$ calcd for $C_{12}H_{11}$ F_3NO_2, 258.0736; found, 258.0732.

Compound 1b. Methacryloyl chloride (0.19 mL, 2.0 mmol) was slowly added to a stirred solution of compound **10** (190 mg, 1.0 mmol) and N, N-diisopropylethylamine (DIPEA, 0.34 mL, 2.0 mmol) in dichloromethane (10 mL) at -20 °C. The reaction mixture warmed up to room temperature and stirred for 6 h, before it was diluted with CH_2Cl_2 (5 mL). The combined organic solution was washed with 1 M HCl (10 mL) and water (2 × 10 mL), dried with magnesium sulfate, filtered, and concentrated by rotary evaporation. The residue was purified by column chromatography over silica gel using 10:1 hexane/ethyl acetate as eluent to afford a light-yellow oil (228 mg, 70 %). ^{1}H NMR (600 MHz, $CDCl_3$, δ): 7.47 (m, 3H), 7.28 (m, 1H), 6.24 (s, 1H), 6.07 (s, 1H), 5.72 (s, 1H), 5.55 (s, 1H), 2.14 (s, 3H), 1.88 (s, 3H). ^{13}C NMR (150 MHz, $CDCl_3$, δ): 163.4, 152.6, 140.1, 136.2,135.4, 131.8, 128.0, 127.6, 126.7, 124.4, 122.2, 121.2 (q, *J* = 283.5 Hz), 115.8, 18.8, 17.7. ESI-HRMS (m/z): $[M+H]^+$ calcd for $C_{16}H_{15}$ F_3NO_3, 326.0998; found, 326.0995.

Compound 1c. 4-Vinylbenzyl chloride (0.14 mL, 2.0 mmol) was added slowly to a stirred mixture of compound **10** (190 mg, 1.0 mmol) and anhydrous K_2CO_3 (138.2 mg, 1.0 mmol) in dry DMF (5 mL). After the reaction mixture was stirred at 80 °C for 6 h, it was diluted with ethyl acetate (30 mL). The combined organic solution was washed with 1 M HCl (10 mL) and brine (2 × 10 mL), dried with magnesium sulfate, filtered, and concentrated by rotary evaporation. The residue was purified by column chromatography over silica gel using 20:1 hexane/ethyl acetate as eluent to afford a light-yellow oil (214 mg, 70 %). ^{1}H NMR (600 MHz, $CDCl_3$, δ): 9.17 (s, 1H), 7.85 (d, *J* = 8.5 Hz,1H), 7.44 (m, 3H), 7.34 (m, 2H), 6.74 (m, 2H), 5.78 (d, *J* = 16.9 Hz,1H), 5.29 (d, *J* = 10.9 Hz,1H), 4.54 (s, 2H). ^{13}C NMR (100 MHz, DMSO-d_6, δ): 178.8 (q, *J* = 31.5 Hz), 152.9, 138.0, 137.8, 136.2, 136.1, 131.2, 127.3, 126.3, 117.0 (q, *J* = 291.0 Hz), 115.3, 114.0, 113.45, 109.85, 45.54. ESI-HRMS (*m/z*): $[M+H]^+$ calcd for $C_{17}H_{15}$ F_3NO, 306.1100; found, 306.1095.

Compound 1d. 4-Vinylbenzyl chloride (0.14 mL, 2.0 mmol) was added slowly to a stirred mixture of compound **12** (190 mg, 1.0 mmol) and anhydrous K_2CO_3 (138.2 mg, 1.0 mmol) in dry DMF (5 mL). After the reaction mixture was stirred at 80 °C for 6 h, it was diluted with ethyl acetate (30 mL). The combined organic solution was washed with 1 M HCl (10 mL) and brine (2 × 10 mL), dried with magnesium sulfate, filtered, and concentrated by rotary evaporation. The residue was purified by column chromatography over silica gel using 15:1 hexane/ethyl acetate as eluent to afford a light-yellow oil (168 mg, 55 %). ^{1}H NMR (600 MHz, $CDCl_3$, δ): 7.92 (m, 2H), 7.41 (m, 2H), 7.28 (m, 2H), 6.72 (m, 1H), 6.63 (m, 2H), 5.76 (d, *J* = 30.0 Hz,1H), 5.27 (d, *J* = 18.0 Hz,1H), 4.44 (s, 2H). ^{13}C NMR (150 MHz, CDCl3, δ): 178.1 (q, *J* = 33.0 Hz), 153.6, 137.4 137.0, 136.3, 133.1,127.6, 126.9, 119.3, 117.4 (q, *J* = 289.5 Hz), 114.4, 112.1, 47.3. ESI-HRMS (*m/z*): $[M+H_3O]^+$ calcd for $C_{17}H_{17}$ F_3NO2, 324.1205; found, 324.1197.

Compound 1e. Methacryloyl chloride (0.19 mL, 2.0 mmol) was slowly added to a stirred solution of compound **12** (190 mg, 1.0 mmol) and N,N-diisopropylethylamine (DIPEA, 0.34 mL, 2.0 mmol) in dichloromethane (10 mL) at -20 °C. The reaction mixture warmed up to room temperature and stirred for 6 h, before it was diluted with CH_2Cl_2 (5 mL). The combined organic solution was washed with 1 M HCl (10 mL) and water (2 × 10 mL), dried with magnesium sulfate, filtered, and concentrated by rotary evaporation. The residue was purified by column chromatography over silica gel using 8:1 hexane/ethyl acetate as eluent to afford a light brown powder (180 mg, 70 %). ^{1}H NMR (600 MHz, $CDCl_3$, δ): 8.08 (d, *J* = 9.0 Hz,2H), 7.95 (s, 1H), 7.80 (d, *J* = 8.9 Hz,2H), 5.87 (s, 1H), 5.59 (s, 1H), 2.10 (s, 3H). ^{13}C NMR (100 MHz, CDCl3, δ): 179.2 (q, *J* = 34.5 Hz), 166.9, 144.4, 140.6, 131.96, 125.5, 121.1, 119.4, 116.9 (q, *J* = 289.5 Hz), 18.7. ESI-HRMS (*m/z*): $[M+H]^+$ calcd for $C_{12}H_{11}F_3NO_2$, 258.0736; found, 258.0729.

Compound 6. 1-Ethyl-3-(3-dimethylaminopropyl)carbodiimide (EDCI, 109 mg, 0.56 mmol) and DIPEA (0.09 mL, 0.56 mmol) was added slowly to a stirred solution of 4-cyano-4-(((ethylthio)carbonothioyl)thio)pentanoic acid (100 mg, 0.37 mmol) in dry DMF (5 mL), followed by compound **13** (56.6 mg, 0.56 mmol). After stirred for another 12 h, the reaction mixture was diluted with ethyl acetate (30 mL). The combined organic solution was washed with 1 M HCl (10 mL) and brine (2 × 20 mL), dried with magnesium sulfate, filtered, and concentrated by rotary evaporation. The residue was purified by column chromatography over silica gel using 10:1 hexane/acetone as eluent to afford a light-yellow oil (90 mg, 70 %). ^{1}H NMR (600 MHz, $CDCl_3$, δ): 4.22 (m, 1H), 3.43 (m, 2H), 3.37 (m, 2H), 2.66 (m, 2H), 2.55 (m, 1H), 2.41 (m, 1H), 1.95 (m, 2H), 1.90 (m, 3H), 1.38 (m, 3H). ^{13}C NMR (150 MHz, CDCl3, δ): 171.3, 118.9, 61.9, 48.1, 46.3, 33.8, 31.4, 29.7, 28.1, 24.9, 12.8. ESI-HRMS (*m/z*): $[M+H]^+$ calcd for $C_{12}H_{19}N_4O_2S_3$, 347.0664; found, 347.0662.

Compound 7. An ammonia solution in methanol (0.5 mL, 2.0 M, 1.0 mmol) was added slowly to a stirred solution of 2-hydrazino-2-imidazoline hydrobromide (180.0 mg, 1.0 mmol) in methanol (1 mL). After 30 min, the volatiles were removed by rotary evaporation. The residue was dissolved in DMF (10 mL), followed by slow addition of DIPEA (0.34 mL, 2.0 mmol) and methacryloyl chloride (0.19 mL, 2.0 mmol). After another 12 h, the reaction mixture was diluted with ethyl acetate (20 mL). The combined organic solution was washed with 1 M NaOH (10 mL) and water (2 × 30 mL), dried with magnesium sulfate, filtered, and concentrated by rotary evaporation. The residue was purified by column chromatography over silica gel using 30:1 hexane/acetone as eluent to afford a light-yellow oil (72 mg, 30 %). ^{1}H NMR (600 MHz, CD_3OD, δ): 5.72 (m, 2H), 5.41 (m, 2H), 3.48 (m, 4H), 2.09 (m, 3H), 1.92 (m, 3H). ^{13}C NMR (100 MHz, CD_3OD, δ): 170.0, 163.8, 159.6, 139.8, 128.4, 119.4, 117.5, 42.0, 38.7, 17.4, 17.0. ESI-HRMS (*m/z*): $[M-H]^+$ calcd for $C_{11}H_{17}N_4O_2$, 237.1346; found, 237.1342.

Compound 8. A solution of compound **14** (34.4 mg, 0.4 mmol) in dichloromethane (1 mL) was added slowly to a stirred solution of lissamine rhodamine B sulfonyl chloride mixed isomer (50 mg, 0.08 mmol) in dry dichloromethane (10 mL) and DMF (2 mL) at -20 °C under argon, followed by triethylamine (0.05 mL, 0.35 mmol). The reaction mixture was stirred at -10°C overnight. After TLC indicated completion of the reaction, the mixture was diluted with dichloromethane (30 mL). The combined organic solution was washed with 1 M NaOH (10 mL) and water (2 × 30 mL), dried with magnesium sulfate, filtered, and concentrated by rotary evaporation. The residue was purified by column chromatography over silica gel using 10% methanol in chloroform as the eluent to afford a pink oil as an isomeric mixture of compound **8** (32 mg, 63 %). ^{1}H NMR (400 MHz, CD_3OD + $CDCl_3$, δ): 8.60 (s, 1H), 8.25 (m, 1H), 7.79 (s, 1H), 7.48 (m, 1H), 7.15 (m, 3H), 7.04 (m, 2H), 6.94 (m, 2H), 6.77 (m, 1H), 6.42 (m, 1H), 3.68 (m, 6H), 3.39 (m, 2H), 3.29 (m, 2H), 3.06 (m, 2H), 3.00 (m, 2H), 1.33 (m, 12H), 1.20 (m, 2H). ^{13}C NMR (100 MHz, CD_3OD + $CDCl_3$, δ):157.8, 155.85, 155.0, 153.0, 149.2, 147.9, 146.8, 140.8, 132.2, 131.7, 131.3, 129.5, 126.0,

114.0, 113.9, 108.5, 105.5, 97.2, 95.9, 48.2, 45.6, 44.1, 41.9, 38.9, 29.4, 11.7. ESI-HRMS (*m/z*): $[M]^+$ calcd for $C_{29}H_{35}N_6O_6S_2$, 627.2054; found 627.2056.

1.3 Dynamic Light Scattering

The particle size of $\mathbf{NP_A}$ and $\mathbf{NP_B}$ was determined on a Malvern Zetasizer Nano ZS using the Zetasizer software according to the Stokes-Einstein equation (S1). The volume of a spherical nanoparticle (V_{D_h}) was calculated from equation (S2). Assuming a density of 1.37 g/cm^3 (the density of protein), the molecular weight of the particle can be calculated using equation (S3).[7] A nanoparticle with a hydrodynamic diameter of 5 nm has a calculated molecular weight of 50 kDa, which was used in making MINP solution for ITC titration.

$$D_h = \frac{k_B T}{6\pi\eta D_t} \qquad (1)$$

in which D_h is the hydrodynamic diameter, D_t the translational diffusion coefficient measured by dynamic light scattering, T the temperature, k_B the Boltzmann's constant, and η is dynamic viscosity of water (0.890 cP at 298 K).

$$V_{D_h} = \frac{4\pi}{3}\left(\frac{D_h}{2}\right)^3 \qquad (2)$$

$$\text{Mw in dalton} = \left(\frac{D_h}{0.132}\right)^3 \qquad (3)$$

in which D_h is the hydrodynamic diameter in nm.

1.4 Determination of Binding Constants by ITC

In general, a solution of an appropriate guest protein in 10 mM (HEPES) buffer (pH 7.5) at 298 K was injected in equal steps into 1.43 mL of the corresponding $\mathbf{NP_A}$ or $\mathbf{NP_B}$ in the same solution. An average molecular weight of 50 kDa $\mathbf{NP_A}$ and 760 kDa for $\mathbf{NP_B}$ for was used for making the stock solutions. The top panel shows the raw calorimetric data. The area under each peak represents the amount of heat generated at each ejection and is plotted against the molar ratio of the $\mathbf{NP_A}$ or $\mathbf{NP_B}$ to the guest protein. The solid line is the best fit of the experimental data to the sequential binding of N equal and independent binding sites on the $\mathbf{NP_A}$ or $\mathbf{NP_B}$. The heat of dilution for the substrate, obtained by adding the substrate to the buffer, was subtracted from the heat released during the binding. Binding parameters were autogenerated after curve fitting using Microcal Origin 7.

1.5 Cytochrome c Oxidase Assay

The Cytochrome c Oxidase Assay Kit (Cytochrome c Oxidase Assay Kit, CYTOCOX1, sigma Aldrich) is designed for the determination of cytochrome c oxidase activity in soluble and membrane bound mitochondrial samples. The colorimetric assay kit is based on observation of the decrease in absorbance at 550 nm of ferrocytochrome c caused by its oxidation to ferricytochrome c by cytochrome c oxidase. Cytochrome c is reduced with dithiothreitol and then re-oxidized by the cytochrome c oxidase. The cytochrome *c* oxidase assay was performed according to the instructions given in the kit. The COX activity of the mitochondrial protein at different concentrations (0–5.0 µg) was measured in the presence of 10.9 µM cytochrome c preincubated with 10–50 µM $\mathbf{NP_B}$(Cytc) for 30 min. Control experiments were performed in the absence of $\mathbf{NP_B}$(Cytc).

1.6 Inhibition of HRP activity

The HRP activity was measured according to a literature procedure, using 3,3´,5,5´-Tetramethylbenzidine (TMB) as the colorimetric substrate.[8, 9] A solution of **NP$_A$**(HRP) or **NP$_B$**(HRP) (10–50 μM) and HRP (0–14 nM) in a 50 mM Tris-HCl buffer (pH= 7.5 with 150 mM NaCl) was prepared. After 30 min at room temperature, a working solution of TMB in 200 mM phosphate/citrate buffer (pH 5.0) was added and the final concentration of TMB in the mixture was 0.40 mM. After 2 min, the absorbance at 650 nm was measured for each sample with a microplate reader. Control experiments were performed in the absence of **NP$_A$**(HRP) or **NP$_B$**(HRP). The TMB working solution was prepared by mixing a solution of TMB (1.0 mg in 1.0 mL in DMSO) with 9.0 mL of the phosphate/citrate buffer, followed by the addition of a solution of 2.0 µL of fresh 30% hydrogen peroxide aqueous solution in 10 ml of the same buffer.

1.7 Cell culture

The MDA-MB-231 cell line was acquired from ATCC and cultured in Dulbecco's Modified Eagle's Medium (DMEM) consisting of 100 mL/L of heat inactivated Fetal Bovine Serum (FBS) and 100 mg/L of antibiotics (penicillin, streptomycin and anti-fungal amphotericn B). The cells were maintained in a humid 5% CO_2-containing incubator until they reached 60–70% confluency. The cells were trypsinized (1 × trypsin) and re-plated in fresh medium overnight prior to the experiments. The viable number of cells was determined using the Trypan blue exclusion test.

1.8 Cytotoxicity Assay

The viable cells were analyzed using the 3(4,5-dimethylthiazolyl-2)2,5-diphenyltetrazolium bromide assay (MTT, Sigma-Aldrich) according to the manufacturer's protocol. The cells were seeded at a concentration of 1.5×10^4 cells per well in 100 μL culture medium onto 96 well plates overnight, followed by treatment with different amounts of **NP$_B$**(Cytc) or **NP$_B$**(Cytc)***** for 24 or 48 h. The previous medium was removed and the cells were reconstituted with colorless DMEM, followed by the addition of MTT to a final concentration of 0.5 mg/mL. The cells containing MTT were incubated for 4 h, followed by the addition of 100 µL DMSO. The MTT-containing cells were rocked gently for 10 min to ensure proper mixing. The absorbances at 570 and 575 nm were measured using a micro plate reader. Cell viability was defined as the percentage of live cells relative to the untreated controls.

1.9 Cellular uptake

Cellular internalization profile of **NP$_B$**(Cytc) was measured by lissamine rhodamine B-labelled **NP$_B$**(Cytc) or **NP$_B$**(Cytc)*. MDA-MB-231 cells were seeded in a 96 well-plate at a density of 1.5×10^4 cells per well and incubated for 24 h. The cells were then incubated with 5–20 µM **NP$_B$**(Cytc)* for 1 h. The cells were washed with a PBS buffer for 3 times before they were imaged under a fluorescent microscope. For confocal microscopy, cells were seeded in a glass slide and fixed with 3.7% formaldehyde before imaging.

1.10 AO/EtBr staining

1×10^5 MDA-MB-231 cells were seeded onto 12-well plates per well and maintained in a humid 5% CO_2-containing incubator. Then the cells were treated with 100 μM of **NP$_B$**(Cytc) for 2 h. The cells were incubated with the appropriate apoptosis inducer (20–60 μM 5-fluorouracil for 48 h or 0.1–0.5 μM Staurosporine for 2 h). Next, the cells were washed, trypsinized and resuspended in PBS. A 5.0 µL aliquot of a mixture of 100 µg/mL AO and 100 µg/mL EB solutions was added to the cells and the cells were incubated in dark for 5 min. Then a 10 μL suspension of cells was spread onto a microscope slide and covered with a glass coverslip. At least 400 cells were examined under a fluorescence microscope.

1.11 Fluorescence-activated cell sorting (FACS)

1×10^5 MDA-MB-231 cells were seeded onto 12-well plates per well and maintained in a humid 5% CO_2-containing incubator. The cells were treated with 5–25 µM **NP$_B$**(Cytc)***** and incubated for 1 h. They were washed with a cold phosphate-buffered saline (PBS) and harvested with 1 × trypsin. The washed cells were centrifugated and resuspended in the FACS-binding buffer to a density of ~1×10^6 cells/mL. The nanoparticle-stained cells were analyzed by flow cytometry in the red channel.

1.12 Apoptosis measurement with Annexin V for Flow Cytometry

The percentage of dead cells were measured using apoptosis kits with annexin V conjugates including Alexa Fluor 488 and PI (Thermo fisher, Catalog number: V13241) following the manufacturer's protocol. Briefly, 3×10^5 cells were seeded onto 6-well plates per well, followed by treatment with different doses (20–100 µM) of **NP$_B$**(Cytc). The cells were incubated with the appropriate apoptosis inducer (20–60 µM 5-fluorouracil for 48 h or 0.1–0.5 µM Staurosporine for 2 h). Control experiments were performed in the absence of **NP$_B$**(Cytc). They were washed with a cold phosphate-buffered saline (PBS) and harvested with 1 × trypsin. The washed cells were centrifugated and resuspended in 1 × annexin-binding buffer to a density of ~1×10^6 cells/mL. Then, 5 µL of Alexa Fluor 488 Annexin V (supplied with kit) and 1 µL of the 100 µg/mL PI working solution was added to each 100 µL of cell suspension. The cells were incubated at room temperature for 15 min in dark, followed by the addition of 400 µL of 1 × annexin-binding buffer. After gentle mixing, the cells were kept on ice before they were analyzed by flow cytometry.

1.13 Endocytosis inhibitory experiments

1×10^5 MDA-MB-231 cells were seeded onto 12-well plates per well and maintained in a humid 5% CO_2-containing incubator. The cells were incubated with 20 µM **NP$_B$**(Cytc)***** and various inhibitors under the following conditions: 10 mM sodium azide/6.0 mM 2-deoxy-D-glucose for 1 h; 5.0 mM methyl-β-cyclodextrin for 30 min; 0.45 M sucrose for 10 min; 20 µg/mL chlorpromazine for 45 min; 1.0 mM amiloride for 30 min. The cells were washed with a cold phosphate-buffered saline (PBS) and harvested with 1 × trypsin. The washed cells were centrifugated and resuspended in the FACS-binding buffer to a density of ~1×10^6 cells/mL. The nanoparticle-stained cells were analyzed by flow cytometry in the red channel.

2. Supplementary Notes

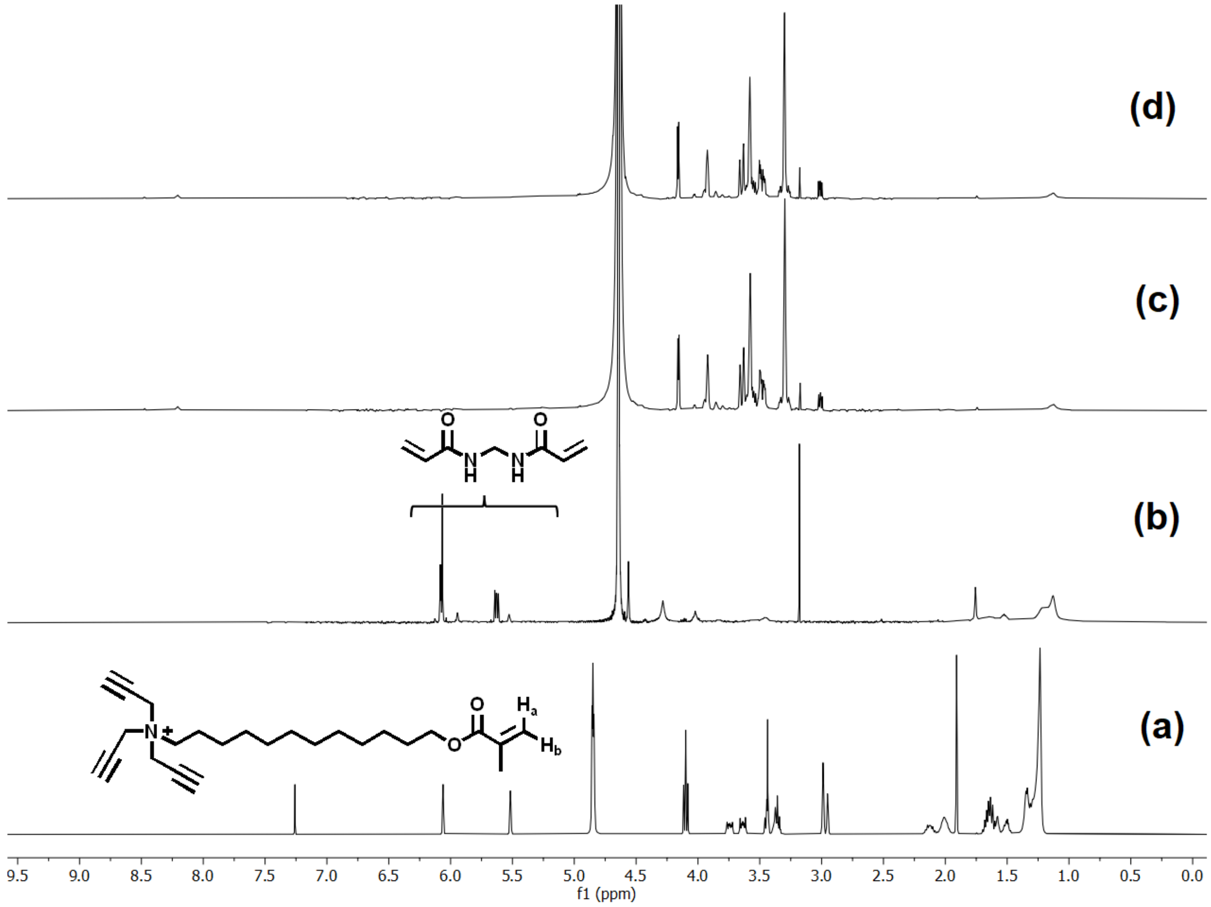

Supplementary Figure 6. Surface-cross-linking and the core-cross-linking of the micelles were monitored by ^{1}H NMR spectroscopy. ^{1}H NMR spectra of (a) surfactant **3** in $CDCl_3$, (b) typical alkynyl surface cross-linked micelles after addition of the protein template in D_2O, (c) typical surface cross-linked micelles after UV irradiation and subsequent functionalization with surface ligand **5**, (d) typical **NP_A** in D_2O.

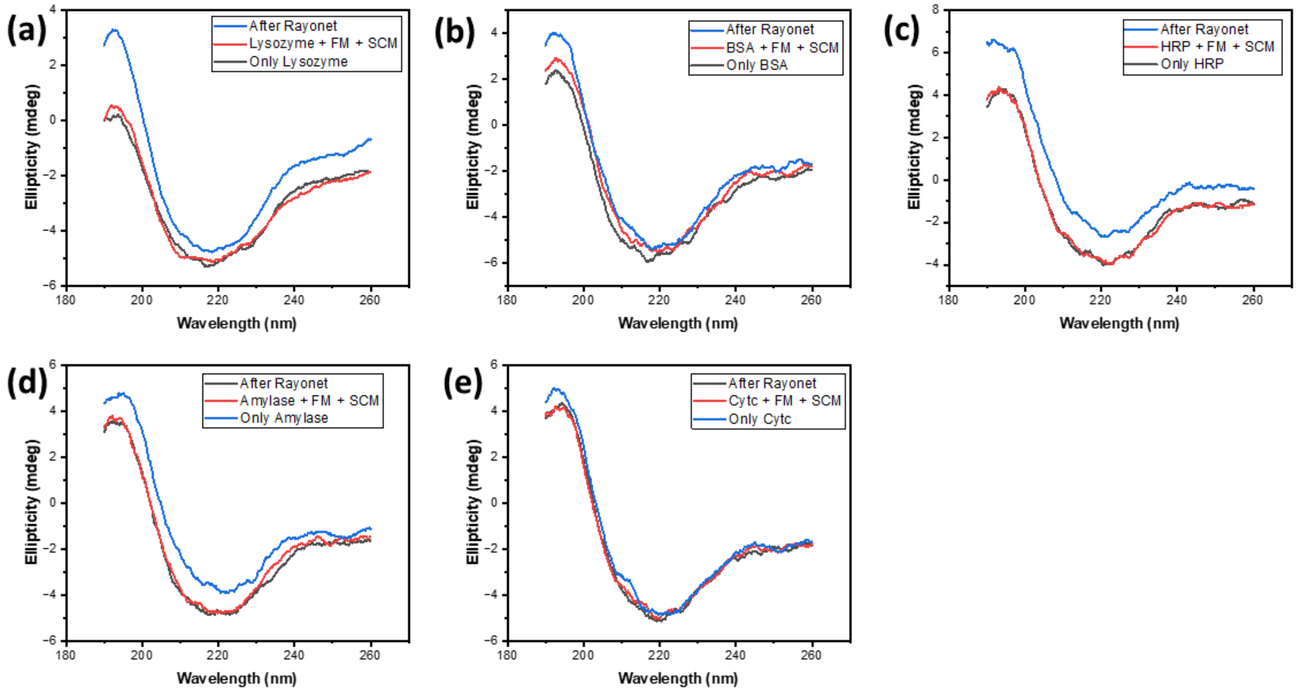

Supplementary Figure 7. Comparison of CD spectra of protein (black), a mixture of protein, FM **1a**, and SCM (red), and the as-prepared **NP$_A$(**protein)/protein mixture (blue) in water. The protein involved is lysozyme (a), BSA (b), HRP (c), amylase (d), and Cytc (e), respectively. [Protein] = 0.5 μM. [FM **1a**]/[SCM]/[lysozyme] = 8:1:1.

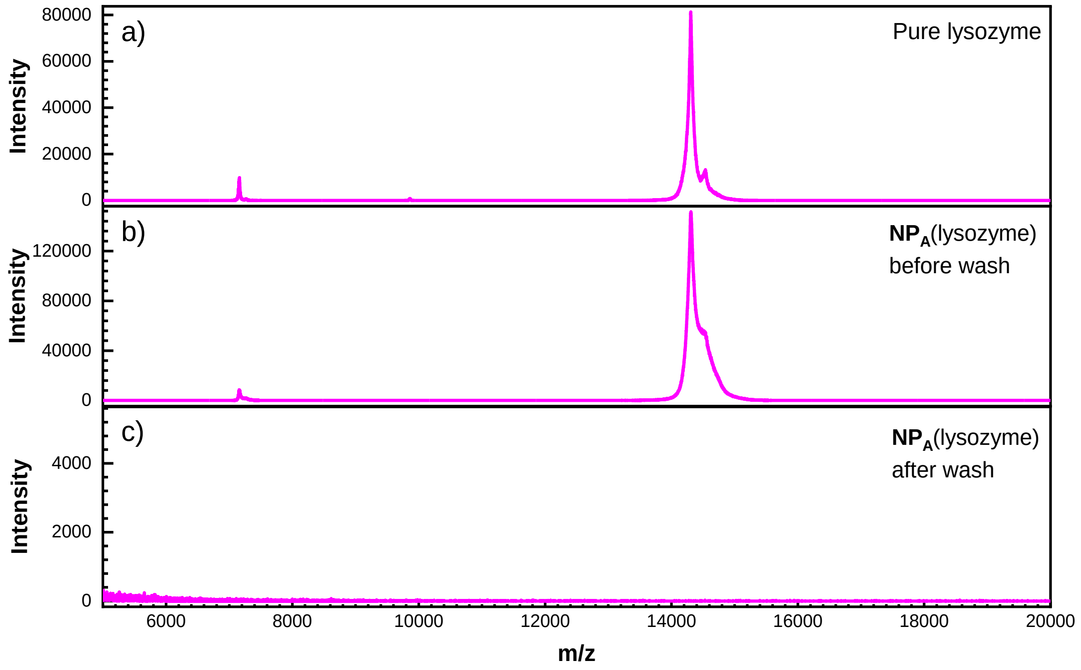

Supplementary Figure 8. MALDI-TOF mass spectrum of (a) lysozyme, (b) $\mathbf{NP_A}$**(**lysozyme)/lysozyme mixture, and (c) $\mathbf{NP_A}$(lysozyme) after purified by precipitation and solvent washing.

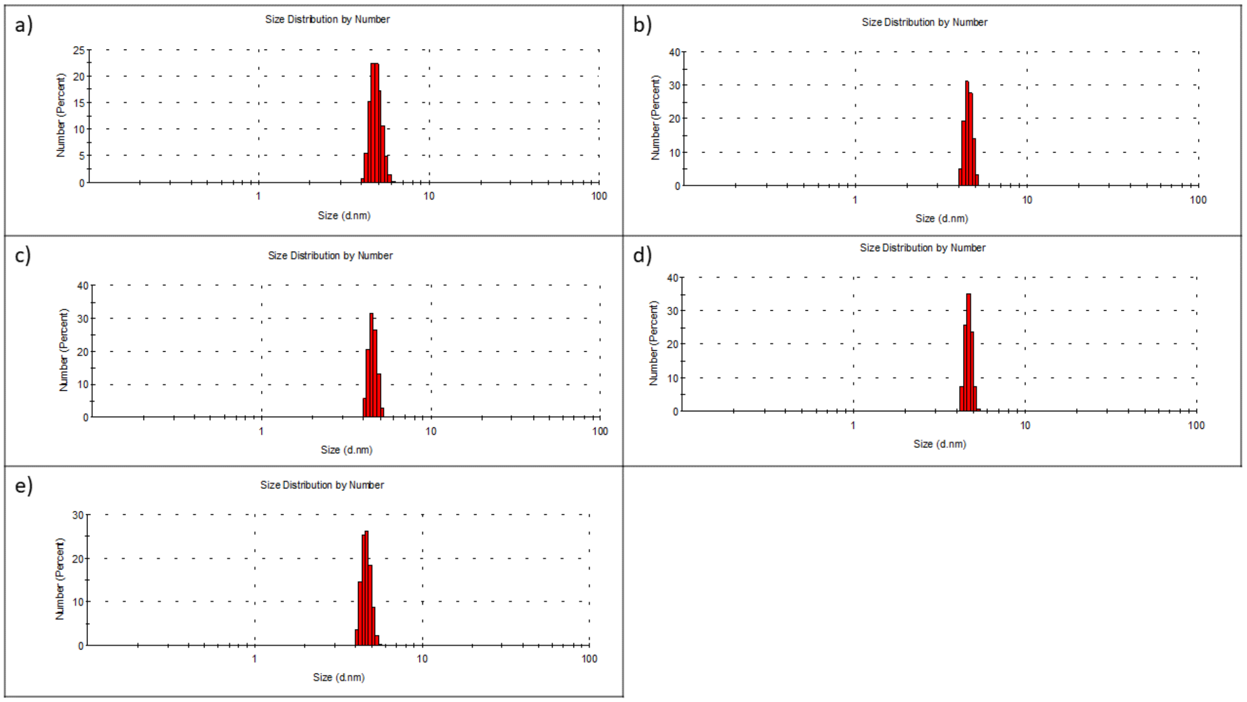

Supplementary Figure 9. Distribution of the hydrodynamic diameters of the nanoparticles in water as determined by DLS for (a) $\mathbf{NP_A}$(lysozyme), (b) $\mathbf{NP_A}$(BSA), (c) $\mathbf{NP_A}$(HRP), (d) $\mathbf{NP_A}$(amylase), and (e) $\mathbf{NP_A}$(cytc).

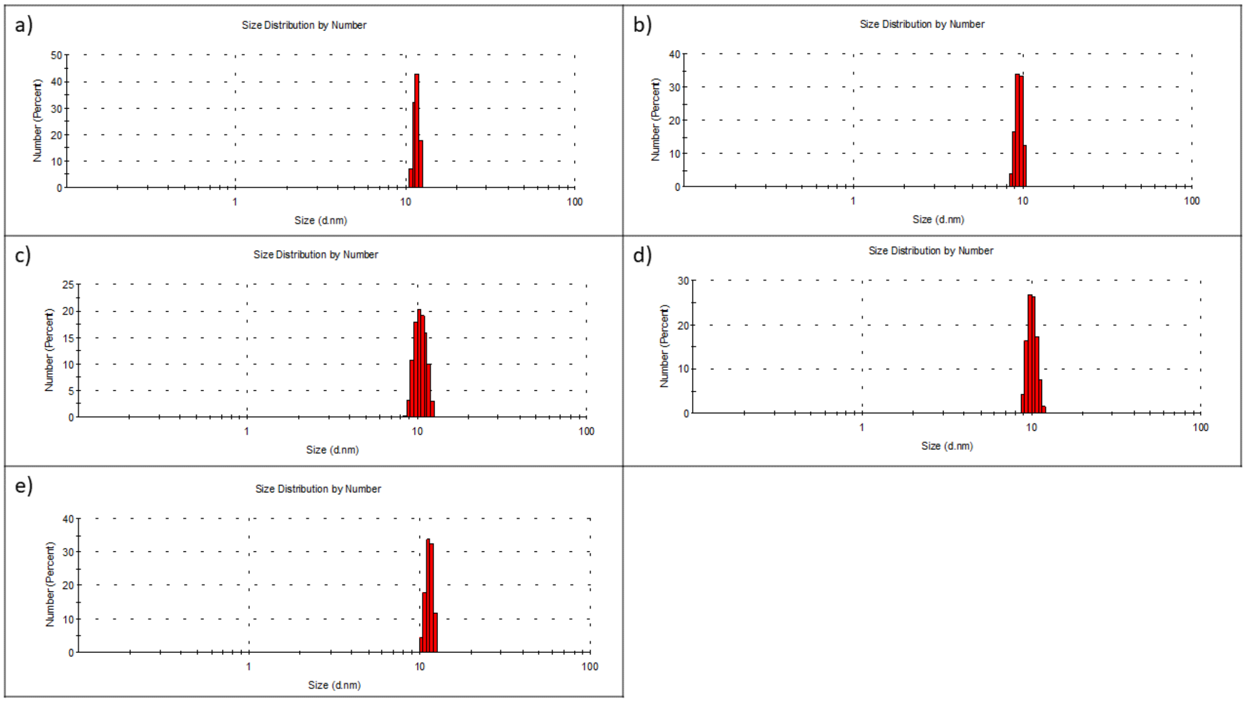

Supplementary Figure 10. Distribution of the hydrodynamic diameters of the nanoparticles in water as determined by DLS for (a) **NP$_B$**(lysozyme), (b) **NP$_B$**(BSA), (c) **NP$_B$**(HRP), (d) **NP$_B$**(amylase), and (e) **NP$_B$**(cytc).

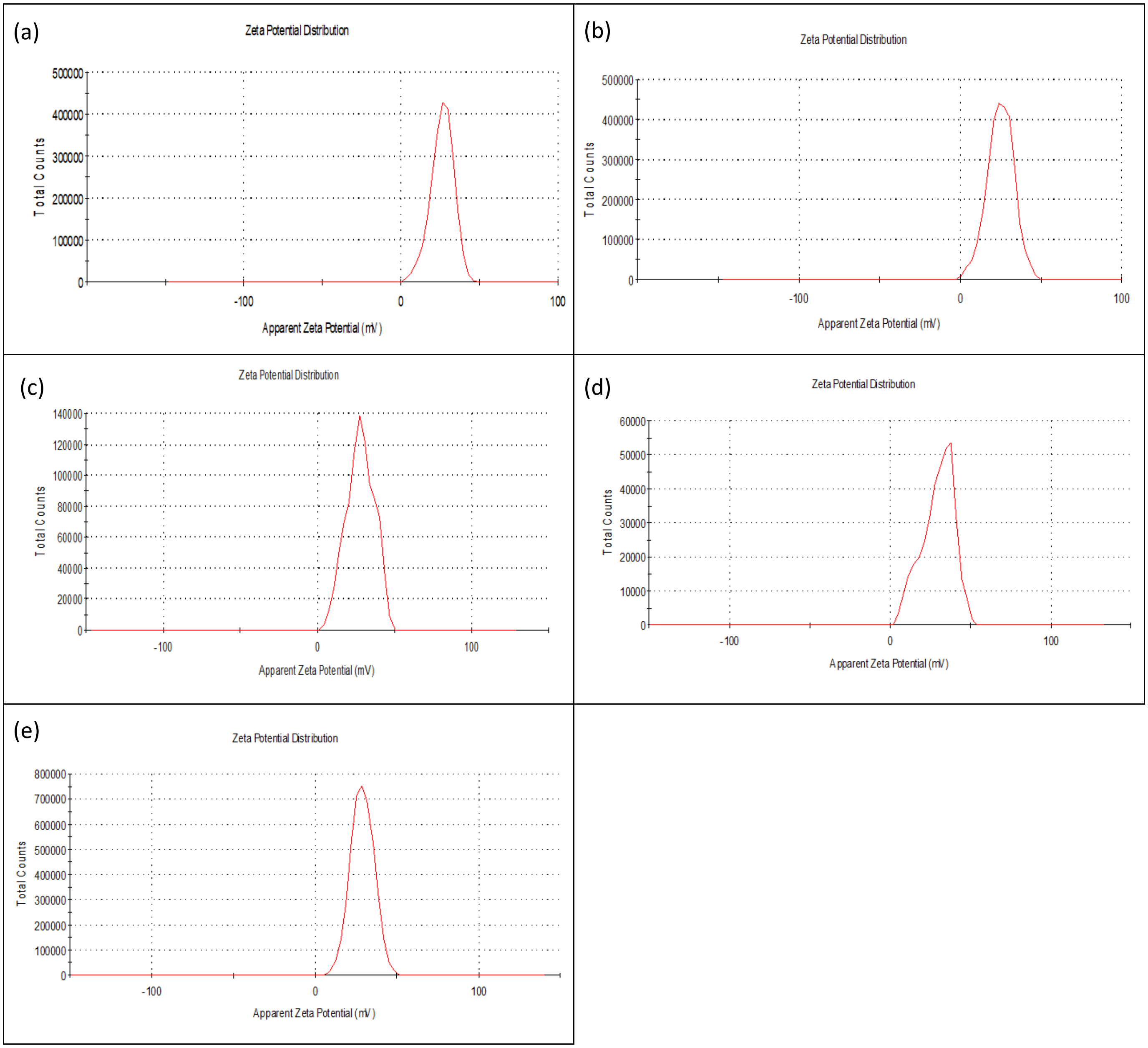

Supplementary Figure 11. Zeta potential of (a) **NP$_A$** (lysozyme), (b) **NP$_A$** (BSA), (c) **NP$_A$** (HRP), (d) **NP$_A$** (amylase), and (e) **NP$_A$** (cytc). The experiments were repeated 3 times with similar results.

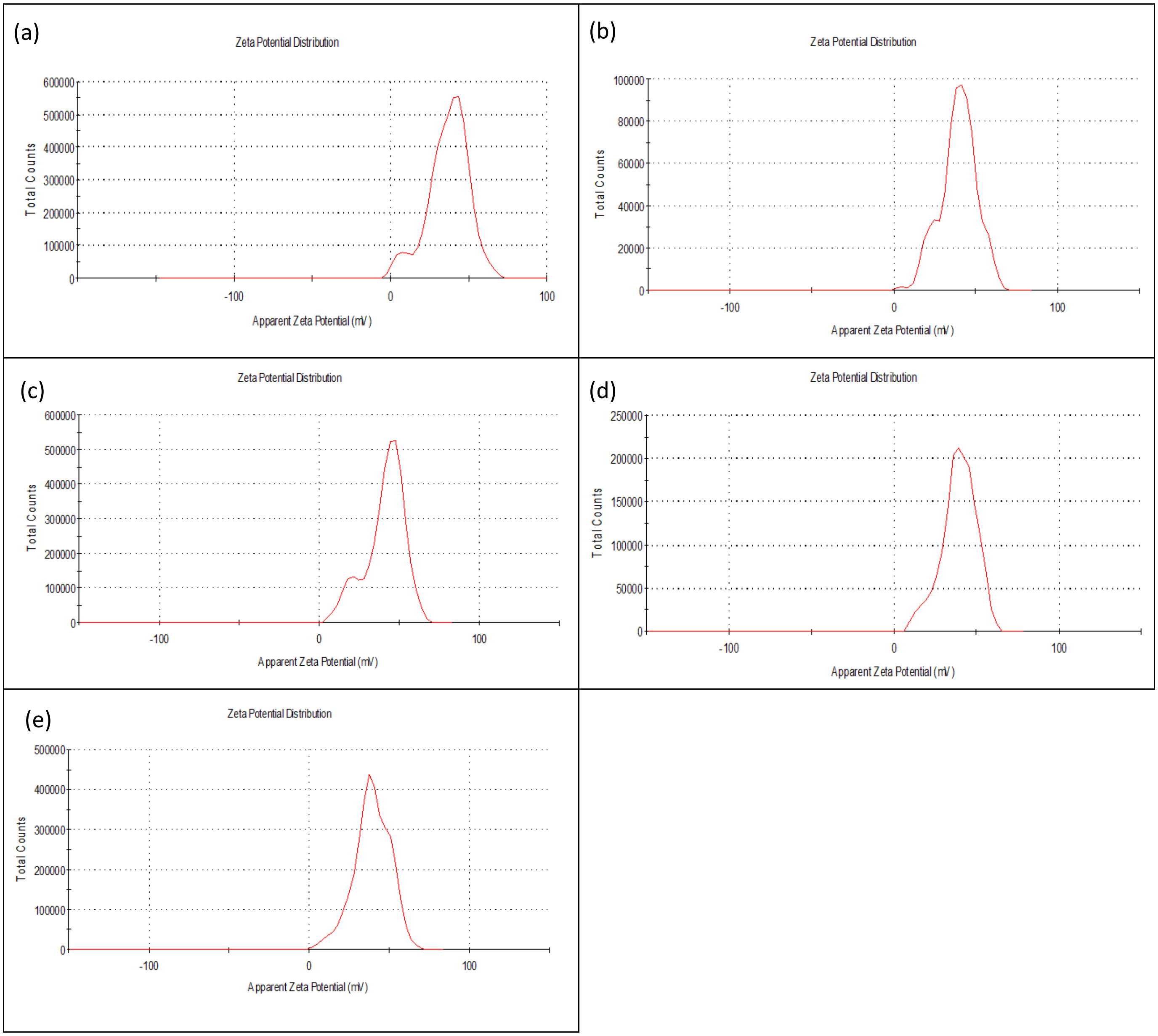

Supplementary Figure 12. Zeta potential of (a) **NP$_B$** (lysozyme), (b) **NP$_B$** (BSA), (c) **NP$_B$** (HRP), (d) **NP$_B$** (amylase), and (e)**NP$_B$** (cytc). The experiments were repeated 3 times with similar results.

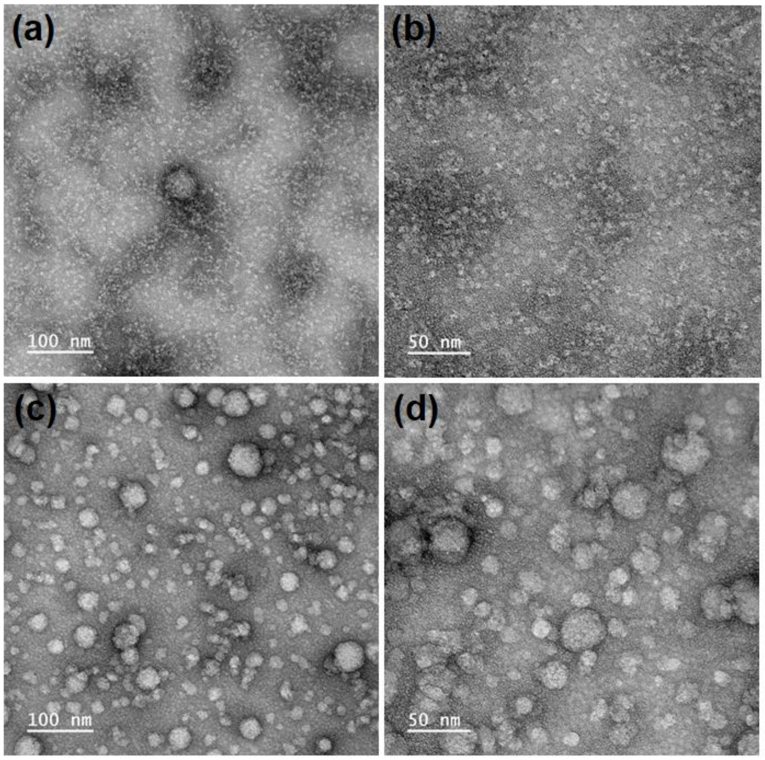

Supplementary Figure 13. TEM image of typical **NP$_A$** (a and b) and **NP$_B$** (c and d). For the TEM imaging, 0.1 mg of **NP$_A$/NP$_B$** was dissolved in 1 mL of Millipore water and the solution was ultra-sonicated for 10 min. A micro syringe was used to load one small drop (~1 μL) of the above solution onto a TEM copper grid covered with carbon film. The sample was left to form a thin layer, and then one small drop (~1 μL) of 2% uranyl acetate solution was loaded on the grid for the negative staining. The sample was left to dry and analyzed on a 200kV JEOL 2100 electron microscope.

Supplementary Table 1. Properties of proteins studied int this work.

Protein	M.W. (KDa)	PI
lysozyme	14	11
BSA	66	4.7–5.0
HRP	44	7.5
α-amylase	54	3.5
cytochrome C	12	11
chymotrypsin	25	8.7
OVA	43	4.5
transferrin	80	5.7
trypsin	23	10.5

Supplementary Table 2. Binding properties of $\mathbf{NP_A}$ (lysozyme) in presence of an interfering protein determined by ITC.[a]

entry	host	interferent[b]	K_a (× 10^5 M^{-1})	-ΔG (kcal/mol)	-ΔH (kcal/mol)	TΔS (kcal/mol)	N[c]
1	$\mathbf{NP_A}$**(**lysozyme**)**	BSA	6.30 ± 0.65	7.92	100.9 ± 2.3	-92.98	0.84 ± 0.01
2	$\mathbf{NP_A}$**(**lysozyme)	HRP	5.96 ± 1.24	7.84	103.2 ± 6.1	-95.36	0.66 ± 0.02
3	$\mathbf{NP_A}$**(**lysozyme**)**	α-amylase	5.47 ± 0.94	7.73	46.47 ± 1.52	-38.74	1.14 ± 0.02
4	$\mathbf{NP_A}$**(**lysozyme**)**	Cytc	5.33 ± 1.67	7.98	197.5 ± 1.9	-189.52	0.50 ± 0.02
5	$\mathbf{NP_A}$**(**lysozyme**)**	chymotrypsin	5.28 ± 0.76	7.72	97.12 ± 3.28	-89.4	1.03 ± 0.02
6	$\mathbf{NP_A}$**(**lysozyme**)**	OVA	6.29 ± 0.75	7.90	28.53 ± 0.49	-20.62	0.83 ± 0.01
7	$\mathbf{NP_A}$**(**lysozyme**)**	transferrin	4.09 ± 0.80	7.63	116.1 ± 7.59	-108.47	0.64 ± 0.02
8	$\mathbf{NP_A}$**(**lysozyme**)**	trypsin	4.24 ± 0.36	7.82	53.12 ± 1.01	-45.30	0.78 ± 0.01

[a]Titrations were performed at 298K in 10mM HEPES buffer (pH=7.5) in triplicates, with the errors <5%. $\mathbf{NP_A}$**(**lysozyme**)** was prepared using an optimized ratio of [**3**]/[**4**]/[**5**]/[MBAm]/[FM]/[lysozyme] = 50:50:100:100:8:1. [b] The protein solution for each cross reactivity experiment contained a ratio of [interferent]/[lysozyme] = 2:1. [c] N is the average number of binding sites per nanoparticle, as measured by ITC curve fitting.

Supplementary Table 3. Binding properties of $\mathbf{NP_A}$(HRP) determined by ITC.[a]

entry	host	guest	K_a (× 10^4 M^{-1})	FM/Template	K_{rel}	-ΔG (kcal/mol)	-ΔH (kcal/mol)	TΔS (kcal/mol)
1	$\mathbf{NP_A}$**(HRP)**	HRP	23.5 ± 1.6	2:1	0.48	7.33	13.12 ± 0.29	-5.79
2	$\mathbf{NP_A}$**(HRP)**	HRP	28.5 ± 1.4	3:1	0.58	7.44	20.36 ± 0.44	-12.92
3	$\mathbf{NP_A}$**(HRP)**	HRP	32.8 ± 2.9	4:1	0.66	7.52	38.36 ± 2.84	-30.84
4	$\mathbf{NP_A}$**(HRP)**	HRP	48.0 ± 4.9	6:1	0.98	7.75	48.87 ± 1.93	-41.12
5	$\mathbf{NP_A}$**(HRP)**	HRP	49.0 ± 2.7	8:1	1	7.76	40.52 ± 1.48	-32.76
6	NINP	HRP	<0.2	6:0	<0.004	-	-	-
7	$\mathbf{NP_A}$**(HRP)**	BSA	4.73 ± 1.6	6:1	0.096	6.38	5.02± 3.38	0.51
8	$\mathbf{NP_A}$**(HRP)**	OVA	<0.03[b]	6:1	<0.0006	-	-	-
9	$\mathbf{NP_A}$**(HRP)**	chymotrypsin	<0.06[b]	6:1	<0.001	-	-	-
10	NP_A(HRP)	transferrin	<0.1 [b]	6:1	<0.002	-	-	-
11	$\mathbf{NP_A}$**(HRP)**	trypsin	<0.005 [b]	6:1	<0.0001	-	-	-
12	$\mathbf{NP_A}$**(HRP)**	Cytc	5.74 ± 1.7	6:1	0.12	6.49	3.89 ± 1.25	2.60
13	$\mathbf{NP_A}$**(HRP)**	Lysozyme	9.74 ± 2.2	6:1	0.20	6.81	2.46 ± 0.22	4.21
14	$\mathbf{NP_A}$**(HRP)**	Amylase	<0.4[b]	6:1	<0.008	-	-	-

[a] Titrations were performed at 298K in 10mM HEPES buffer (pH=7.5) in triplicates, with the errors <5%. $\mathbf{NP_A}$(HRP**)** was prepared using an optimized ratio of [**3**]/[**4**]/[**5**]/[MBAm]/[FM]/[HRP] = 50:50:100:100:6:1. [b]The binding constant was estimated from ITC due to the weak binding. Because of the weak binding, the titration curve fits poorly and has large uncertainties in the estimated binding constant.

Supplementary Table 4. Binding properties of **NP_B**(HRP) determined by ITC.[a]

entry	host	guest	K_a ($\times 10^5 M^{-1}$)	[**7**]/template	K_{rel}	-Δ*G* (kcal/mol)	-Δ*H* (kcal/mol)	TΔ*S* (kcal/mol)
1	**NP_B(HRP)**	HRP	10.3 ± 1.1	1:0	0.32	8.20	47.16 ± 1.19	-38.96
2	**NP_B(HRP)**	HRP	13.2 ± 2.0	1:2	0.41	8.35	43.35 ± 1.31	-35.00
3	**NP_B(HRP)**	HRP	18.9 ± 4.1	1:4	0.59	8.56	29.64 ± 1.62	-21.08
4	**NP_B(HRP)**	HRP	24.4 ± 2.2	1:6	0.76	8.71	83.43 ± 1.69	-74.72
5	**NP_B(HRP)**	HRP	32.1 ± 3.3	1:8	1	8.88	49.21 ± 1.38	-40.33
6	**NP_B(HRP)**	HRP	28.4± 6.3	1:10	0.88	8.80	64.09 ± 3.23	-55.29
7	NINP	HRP	<0.1[b]	1:8	<0.003	-	-	-
8	**NP_B(HRP)**	BSA	0.91 ± 0.1	1:8	0.028	6.77	8.41 ± 0.69	-1.64
9	**NP_B(HRP)**	OVA	0.34 ± 0.6	1:8	0.011	6.19	19.19 ± 10.66	-13.00
10	**NP_B(HRP)**	chymotrypsin	<0.02[b]	1:8	<0.001	-	-	-
11	**NP_B(HRP)**	transferrin	<0.0002[b]	1:8	<0.0001	-	-	-
12	**NP_B(HRP)**	trypsin	<0.02[b]	1:8	<0.001	-	-	-
13	**NP_B(HRP)**	Cytc	0.34 ± 0.1	1:8	0.011	6.18	5.59 ± 2.93	0.59
14	**NP_B(HRP)**	Lysozyme	0.68 ± 0.3	1:8	0.021	6.59	15.62 ± 5.83	-9.03
15	**NP_B(HRP)**	Amylase	<0.01	1:8	<0.0003	-	-	-

[a] Titrations were performed at 298K in 10mM HEPES buffer (pH=7.5) in triplicates, with the errors <5%. **NP_B(HRP)** was prepared in the following formulation unless otherwise indicated: [3]/[4]/[5]/[MBAm]/[FM]/[HRP] = 50:50:100:100:6:1 for **NP_A**; [6]/[7]/[MBAm]/[AM]/[3]= 0.05:0.2:4.8:31.6: 1 for **NP_B**. [b] The binding constant was estimated from ITC due to the weak binding. Because of the weak binding, the titration curve fits poorly and has large uncertainties in the estimated binding constant.

Supplementary Table 5. Binding properties of **NP_A**(BSA) determined by ITC[a]

entry	host	guest	K_a ($\times 10^4 M^{-1}$)	FM/Template	K_{rel}	-Δ*G* (kcal/mol)	-Δ*H* (kcal/mol)	TΔ*S* (kcal/mol)
1	**NP_A(BSA)**	BSA	26.6 ± 1.2	2:1	0.49	7.40	41.67± 0.56	-34.27
2	**NP_A(BSA)**	BSA	31.5 ± 2.5	4:1	0.58	7.50	31.38 ± 0.96	-23.88
3	**NP_A(BSA)**	BSA	35.0 ± 2.8	8:1	0.64	7.56	24.73 ± 0.74	-17.17
4	**NP_A(BSA)**	BSA	42.6 ± 1.4	10:1	0.78	7.68	19.81 ± 0.16	-12.13
5	**NP_A(BSA)**	BSA	50.3 ± 7.5	15:1	0.92	7.78	66.98 ± 1.57	-59.20
6	**NP_A(BSA)**	BSA	54.6 ± 6.9	20:1	1	7.83	64.98 ± 1.23	-57.15
7	**NP_A(BSA)**	BSA	51.2 ± 7.1	25:1	0.94	7.79	73.33 ± 1.33	-65.54
8	**NINP**	BSA	<0.02[b]	20:1	<0.004	-	-	-
9	**NP_A(BSA)**	OVA	9.03 ± 0.4	6:1	0.17	6.76	0.58 ± 0.01	6.18
10	NP_A(BSA)	chymotrypsin	4.54 ± 0.5	20:1	0.08	6.35	0.64 ± 0.04	5.71
11	**NP_A(BSA)**	transferrin	<0.04[b]	20:1	<0.0007	-	-	-
12	**NP_A(BSA)**	trypsin	<0.001[b]	20:1	<0.00002	-	-	-
13	**NP_A(BSA)**	Cytc	<0.6	20:1	<0.01	-	-	-
14	**NP_A(BSA)**	HRP	6.65 ± 1.1	20:1	0.12	6.58	0.55 ± 0.04	6.03
15	**NP_A(BSA)**	Amylase	1.42 ± 0.2	20:1	0.03	5.66	1.07 ± 0.20	4.59
16	**NP_A(BSA)**	Lysozyme	<0.8[b]	20 :1	<0.02	-	-	-

[a] Titrations were performed at 298K in 10mM HEPES buffer (pH=7.5) in triplicates, with the errors <5%. **NP_A(BSA)** was prepared using an optimized ratio of [3]/[4]/[5]/[MBAm]/[FM]/[BSA] = 50:50:100:100:20:1. [b] The binding constant was

estimated from ITC due to the weak binding. Because of the weak binding, the titration curve fits poorly and has large uncertainties in the estimated binding constant.

Supplementary Table 6. Binding properties of $\mathbf{NP_B}$(BSA) determined by ITC.[a]

entry	host	guest	K_a (× 10^5 M^{-1})	[**7**]/template	K_{rel}	-Δ*G* (kcal/mol)	-Δ*H* (kcal/mol)	TΔ*S* (kcal/mol)
1	$\mathbf{NP_B}$**(BSA)**	BSA	13.9 ± 1.6	1:2	0.44	8.38	70.19 ± 1.87	-61.81
2	$\mathbf{NP_B}$**(BSA)**	BSA	18.0 ± 2.3	1:0	0.58	8.53	49.93 ± 1.53	-41.40
3	$\mathbf{NP_B}$**(BSA)**	BSA	21.4 ± 3.3	1:4	0.68	8.64	68.51 ± 2.10	-59.87
4	$\mathbf{NP_B}$**(BSA)**	BSA	23.2 ± 3.0	1:8	0.74	8.68	52.06 ± 1.68	-43.38
5	$\mathbf{NP_B}$**(BSA)**	BSA	28.8 ± 7.6	1:12	0.92	8.81	47.77 ± 2.85	-38.96
6	$\mathbf{NP_B}$**(BSA)**	BSA	31.3 ± 6.5	1:16	1	8.86	73.66 ± 3.21	-64.80
7	$\mathbf{NP_B}$**(BSA)**	BSA	30.0 ± 6.6	1:20	0.96	8.84	88.92 ± 4.88	-80.08
8	NINP	BSA	<0.03[b]	1:0	<0.001	-	-	-
9	$\mathbf{NP_B}$**(BSA)**	OVA	0.97 ± 0.06	1:8	0.030	6.80	12.74 ± 0.73	-5.94
10	$\mathbf{NP_B}$**(BSA)**	chymotrypsin	<0.09 [b]	1:8	<0.003	-	-	-
11	$\mathbf{NP_B}$**(BSA)**	transferrin	<0.09 [b]	1:8	<0.003	-	-	-
12	$\mathbf{NP_B}$**(BSA)**	trypsin	<0.04 [b]	1:8	<0.001	-	-	-
13	$\mathbf{NP_B}$**(BSA)**	Cytc	0.41 ± 0.37	1:8	0.013	6.29	2.45 ± 1.27	3.84
14	$\mathbf{NP_B}$**(BSA)**	HRP	<0.09 [b]	1:8	<0.003	-	-	-
15	$\mathbf{NP_B}$**(BSA)**	Amylase	0.26± 0.03	1:8	0.008	6.02	23.17 ± 9.39	-29.7
16	$\mathbf{NP_B}$**(BSA)**	Lysozyme	0.64 ± 0.42	1:8	0.020	6.56	19.9 ± 1.40	-23.8

[a] Titrations were performed at 298K in 10mM HEPES buffer (pH=7.5) in triplicates, with the errors <5%. $\mathbf{NP_B}$**(**BSA**)** was prepared in the following formulation unless otherwise indicated: [3]/[4]/[5]/[MBAm]/[FM]/[BSA] = 50:50:100:100:20:1 for $\mathbf{NP_A}$; [6]/[7]/[MBAm]/[AM]/[3]= 0.05:0.2:4.8:31.6: 1 for $\mathbf{NP_B}$. [b]The binding constant was estimated from ITC due to the weak binding. Because of the weak binding, the titration curve fits poorly and has large uncertainties in the estimated binding constant.

Supplementary Table 7. Binding properties of $\mathbf{NP_A}$(Amylase) determined by ITC.[a]

entry	host	guest	K_a (× 10^4 M^{-1})	FM/Template	K_{rel}	-Δ*G* (kcal/mol)	-Δ*H* (kcal/mol)	TΔ*S* (kcal/mol)
1	$\mathbf{NP_A}$**(**Amylase**)**	Amylase	21.3 ± 1.5	4:1	0.39	7.27	41.98 ± 0.93	-34.71
2	$\mathbf{NP_A}$**(**Amylase**)**	Amylase	32.5 ± 1.8	6:1	0.59	7.52	31.38 ± 0.53	-23.86
3	$\mathbf{NP_A}$**(**Amylase**)**	Amylase	46.1 ± 4.0	8:1	0.84	7.73	51.81 ± 0.96	-44.08
4	$\mathbf{NP_A}$**(**Amylase**)**	Amylase	54.9 ± 5.8	10:1	1	7.83	63.02 ± 1.04	-55.19
5	$\mathbf{NP_A}$**(**Amylase**)**	Amylase	52.1± 4.9	12:1	0.95	7.80	86.18 ± 2.06	-78.38
6	NINP	Amylase	<0.3 [b]	6:0	<0.005	-	-	-
7	$\mathbf{NP_A}$**(**Amylase**)**	BSA	4.11 ± 0.79	10:1	0.075	6.29	6.17 ± 1.28	0.12
8	$\mathbf{NP_A}$**(**Amylase**)**	OVA	<0.07 [b]	10:1	<0.001	-	-	-
9	$\mathbf{NP_A}$**(**Amylase**)**	chymotrypsin	<0.0005 [b]	10:1	<0.0001	-	-	-
10	$\mathbf{NP_A}$**(**Amylase**)**	transferrin	<0.09 [b]	10:1	<0.001	-	-	-
11	$\mathbf{NP_A}$**(**Amylase**)**	trypsin	<0.09 [b]	10:1	<0.001	-	-	-
12	$\mathbf{NP_A}$**(**Amylase**)**	Cytc	<0.1 [b]	10:1	<0.001	-	-	-
13	$\mathbf{NP_A}$**(**Amylase**)**	Lysozyme	7.38 ± 1.24	10:1	0.13	6.64	5.24 ± 0.66	1.40
14	$\mathbf{NP_A}$**(**Amylase**)**	HRP	5.38 ± 0.70	10:1	0.10	6.45	6.42 ± 0.74	0.03

[a] Titrations were performed at 298K in 10mM HEPES buffer (pH=7.5) in triplicates, with the errors <5%. $\mathbf{NP_A}$**(**Amylase**)** was prepared using an optimized ratio of [3]/[4]/[5]/[MBAm]/[FM]/[Amylase] = 50:50:100:100:10:1. [b] The binding constant was estimated from

ITC due to the weak binding. Because of the weak binding, the titration curve fits poorly and has large uncertainties in the estimated binding constant.

Supplementary Table 8. Binding properties of **NP$_B$**(Amylase) determined by ITC.[a]

entry	host	guest	K_a (× 10^5 M^{-1})	[**7**]/template	K_{rel}	-ΔG (kcal/mol)	-ΔH (kcal/mol)	TΔS (kcal/mol)
1	**NP$_B$(**Amylase**)**	Amylase	12.1 ± 2.6	1:0	0.36	8.30	50.16 ± 2.70	-41.86
2	**NP$_B$(**Amylase**)**	Amylase	18.8 ± 1.9	1:2	0.56	8.56	15.69 ± 0.49	-7.13
3	**NP$_B$** (Amylase**)**	Amylase	23.7 ± 1.6	1:4	0.70	8.70	74.30 ± 1.20	-65.60
4	**NP$_B$(**Amylase**)**	Amylase	25.7 ± 3.5	1:6	0.76	8.74	61.16 ± 2.05	52.42
5	**NP$_B$(**Amylase**)**	Amylase	26.9 ± 5.3	1:8	0.80	8.77	40.30 ± 1.74	-31.53
6	**NP$_B$(**Amylase**)**	Amylase	33.8 ± 5.0	1:10	1	8.91	13.02 ± 0.42	-4.11
7	**NP$_B$(**Amylase**)**	Amylase	28.4 ± 4.0	1:12	0.84	8.80	22.39 ± 0.76	-13.59
8	NINP	Amylase	<0.1 [b]	1:0	<0.002	-	-	-
9	**NP$_B$(**Amylase**)**	BSA	0.33 ± 0.15	1:10	0.009	6.16	10.78 ± 8.21	-4.62
10	**NP$_B$(**Amylase**)**	OVA	0.44 ± 0.14	1:10	0.0130	6.33	27.57 ± 4.92	-21.24
10	**NP$_B$(**Amylase**)**	chymotrypsin	<0.07 [b]	1:10	<0.002	-	-	-
11	**NP$_B$(**Amylase**)**	transferrin	<0.07 [b]	1:10	<0.002	-	-	-
12	**NP$_B$(**Amylase**)**	trypsin	<0.005 [b]	1:10	<0.0001	-	-	-
13	**NP$_B$(**Amylase**)**	Cytc	0.37 ± 0.05	1:10	<0.011	6.23	5.43 ± 4.17	0.80
14	**NP$_B$(**Amylase**)**	Lysozyme	0.82 ± 0.68	1:10	0.024	6.70	11.57 ± 0.30	-4.87
15	**NP$_B$(**Amylase**)**	HRP	<0.03 [b]	1:10	<0.0008	-	-	-

[a] Titrations were performed at 298K in 10mM HEPES buffer (pH=7.5) in triplicates, with the errors <5%. **NP$_B$(**Amylase**)** was prepared in the following formulation unless otherwise indicated: [3]/[4]/[5]/[MBAm]/[FM]/[Amylase] = 50:50:100:100:10:1. for **NP$_A$**; [6]/[7]/[MBAm]/[AM]/[3]= 0.05:0.2:4.8:31.6: 1 for **NP$_B$**. [b] The binding constant was estimated from ITC due to the weak binding. Because of the weak binding, the titration curve fits poorly and has large uncertainties in the estimated binding constant.

Supplementary Table 9. Binding properties of **NP$_A$**(Cytc) determined by ITC.[a]

entry	host	guest	K_a (× 10^4 M^{-1})	FM/Template	K_{rel}	-ΔG (kcal/mol)	-ΔH (kcal/mol)	TΔS (kcal/mol)
1	**NP$_A$(**Cytc**)**	Cytc	25.4 ± 2.8	4:1	0.45	7.37	15.71 ± 0.78	-8.34
2	**NP$_A$(**Cytc**)**	Cytc	35.5 ± 1.7	6:1	0.63	7.57	26.47 ± 0.53	-18.90
3	**NP$_A$(**Cytc**)**	Cytc	44.6 ± 4.5	8:1	0.79	7.71	18.94 ± 0.89	-11.23
4	**NP$_A$(**Cytc**)**	Cytc	56.7 ± 3.1	10:1	1	7.85	23.30 ± 0.37	-15.45
5	**NP$_A$(**Cytc**)**	Cytc	54.0 ± 2.8	12:1	0.95	7.82	25.29 ± 0.43	-17.47
6	NINP	Cytc	<0.09 [b]	10:1	<0.002	-	-	-
7	**NP$_A$(**Cytc**)**	BSA	6.26 ± 0.83	10:1	0.11	6.54	5.51 ± 0.31	1.03
8	**NP$_A$(**Cytc**)**	OVA	12.9 ± 2.2	10:1	0.22	6.97	14.53 ± 1.56	-7.56
9	**NP$_A$(**Cytc**)**	chymotrypsin	<0.3 [b]	10:1	<0.005	-	-	-
10	**NP$_A$(**Cytc**)**	transferrin	<0.3 [b]	6:0	<0.005	-	-	-
11	**NP$_A$(**Cytc**)**	trypsin	<0.0006 [b]	10:1	<0.0001	-	-	-
12	**NP$_A$(**Cytc**)**	Amylase	<0.001 [b]	10:1	<0.0001	-	-	-
13	**NP$_A$(**Cytc**)**	Lysozyme	7.81 ± 1.86	10:1	0.14	6.67	11.83 ± 3.42	-5.16
14	**NP$_A$(**Cytc**)**	HRP	<0.5 [b]	10:1	<0.009	-	-	-

[a] Titrations were performed at 298K in 10mM HEPES buffer (pH=7.5) in triplicates, with the errors <5%. **NP$_A$(**Cytc**)** was prepared using an optimized ratio of [3]/[4]/[5]/[MBAm]/[FM]/[Cytc] = 50:50:100:100:10:1. [b] The binding constant was estimated from ITC due to the weak binding. Because of the weak binding, the titration curve fits poorly and has large uncertainties in the estimated binding constant.

Supplementary Table 10. Binding properties of **NP$_B$**(cytochrome-C) determined by ITC.[a]

entry	host	guest	K_a ($\times 10^5$ M^{-1})	[**7**]/template	K_{rel}	-Δ*G* (kcal/mol)	-Δ*H* (kcal/mol)	TΔ*S* (kcal/mol)
1	**NP$_B$(**Cytc**)**	Cytc	9.24 ± 1.38	1:0	0.22	8.14	46.99 ± 2.17	-38.85
2	**NP$_B$(**Cytc**)**	Cytc	15.2 ± 2.2	1:2	0.37	8.43	70.32 ± 3.37	-61.89
3	**NP$_B$** (Cytc**)**	Cytc	21.1 ± 1.5	1:4	0.51	8.63	84.89 ± 1.41	-76.26
4	**NP$_B$(**Cytc**)**	Cytc	25.2 ± 5.7	1:6	0.61	8.73	81.33 ± 4.62	-72.60
5	**NP$_B$(**Cytc**)**	Cytc	33.6 ± 7.5	1:8	0.81	8.83	18.94 ± 0.83	-10.11
6	**NP$_B$(**Cytc**)**	Cytc	41.4 ± 14.9	1:10	1	9.03	72.92 ± 5.07	-63.89
7	**NP$_B$(**Cytc**)**	Cytc	37.4 ± 7.7	1:12	0.90	8.97	92.88 ± 3.33	-83.91
8	NINP	Cytc	< 0.02 [b]	1:10	<0.0005	-	-	-
9	**NP$_B$(**Cytc**)**	BSA	0.87 ± 0.16	1:10	0.021	6.74	26.76 ± 6.66	-20.02
10	**NP$_B$(**Cytc**)**	OVA	0.72 ± 0.09	1:10	0.017	6.63	31.52 ± 4.93	-24.89
11	**NP$_B$(**Cytc**)**	chymotrypsin	<0.0007 [b]	1:10	<0.00002	-	-	-
12	**NP$_B$(**Cytc**)**	transferrin	<0.007 [b]	1:10	<0.0002	-	-	-
13	**NP$_B$(**Cytc**)**	trypsin	<0.00003[b]	1:10	<0.00001	-	-	-
14	**NP$_B$(**Cytc**)**	Amylase	0.48 ± 0.22	1:10	0.012	6.38	6.07 ± 4.79	-4.2
15	**NP$_B$(**Cytc**)**	Lysozyme	0.47 ± 0.18	1:10	0.011	6.37	9.02 ± 6.12	-23.6
16	**NP$_B$(**Cytc**)**	HRP	0.48± 0.06	1:10	0.012	6.38	12.0 ± 2.40	-4.5

[a] Titrations were performed at 298K in 10mM HEPES buffer (pH=7.5) in triplicates, with the errors <5%. **NP$_B$(**Cytc**)** was prepared in the following formulation unless otherwise indicated: [3]/[4]/[5]/[MBAm]/[FM]/[Cytc] = 50:50:100:100:10:1. for **NP$_A$**; [6]/[7]/[MBAm]/[AM]/[3]= 0.05:0.2:4.8:31.6: 1 for **NP$_B$**. [b]The binding constant was estimated from ITC due to the weak binding. Because of the weak binding, the titration curve fits poorly and has large uncertainties in the estimated binding constant.

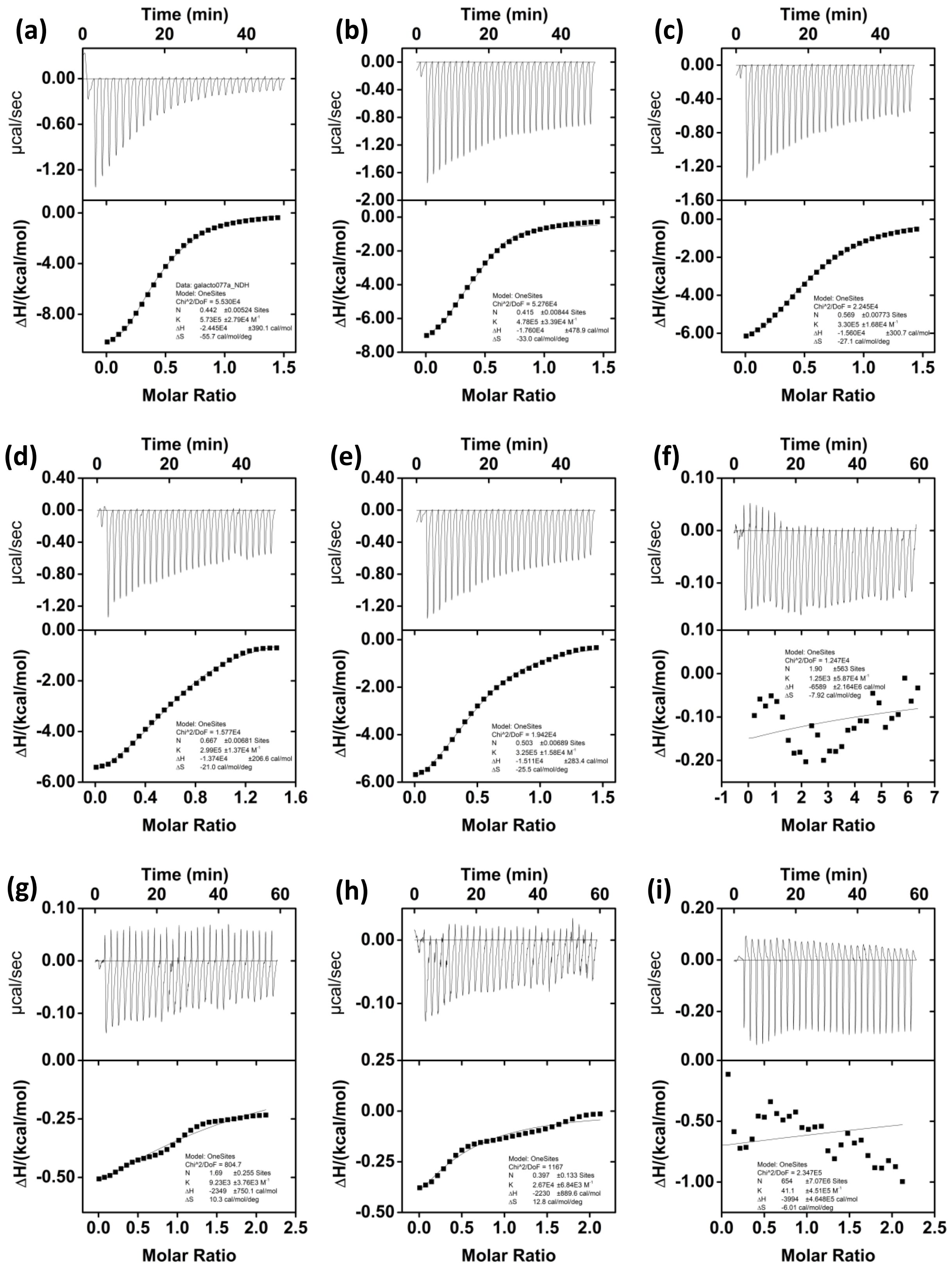

(a)
Time (min)
µcal/sec
ΔH/(kcal/mol)
Molar Ratio
Data: galacto077a_NDH
Model: OneSites
Chi^2/DoF = 5.530E4
N 0.442 ±0.00524 Sites
K 5.73E5 ±2.79E4 M-1
ΔH -2.445E4 ±390.1 cal/mol
ΔS -55.7 cal/mol/deg
(b)
Time (min)
µcal/sec
ΔH/(kcal/mol)
Molar Ratio
Model: OneSites
Chi^2/DoF = 5.276E4
N 0.415 ±0.00844 Sites
K 4.78E5 ±3.39E4 M-1
ΔH -1.760E4 ±478.9 cal/mol
ΔS -33.0 cal/mol/deg
(c)
Time (min)
µcal/sec
ΔH/(kcal/mol)
Molar Ratio
Model: OneSites
Chi^2/DoF = 2.245E4
N 0.569 ±0.00773 Sites
K 3.30E5 ±1.68E4 M-1
ΔH -1.560E4 ±300.7 cal/mol
ΔS -27.1 cal/mol/deg
(d)
Time (min)
µcal/sec
ΔH/(kcal/mol)
Molar Ratio
Model: OneSites
Chi^2/DoF = 1.577E4
N 0.667 ±0.00681 Sites
K 2.99E5 ±1.37E4 M-1
ΔH -1.374E4 ±206.6 cal/mol
ΔS -21.0 cal/mol/deg
(e)
Time (min)
µcal/sec
ΔH/(kcal/mol)
Molar Ratio
Model: OneSites
Chi^2/DoF = 1.942E4
N 0.503 ±0.00689 Sites
K 3.25E5 ±1.58E4 M-1
ΔH -1.511E4 ±283.4 cal/mol
ΔS -25.5 cal/mol/deg
(f)
Time (min)
µcal/sec
ΔH/(kcal/mol)
Molar Ratio
Model: OneSites
Chi^2/DoF = 1.247E4
N 1.90 ±563 Sites
K 1.25E3 ±5.87E4 M-1
ΔH -6589 ±2.164E6 cal/mol
ΔS -7.92 cal/mol/deg
(g)
Time (min)
µcal/sec
ΔH/(kcal/mol)
Molar Ratio
Model: OneSites
Chi^2/DoF = 804.7
N 1.69 ±0.255 Sites
K 9.23E3 ±3.76E3 M-1
ΔH -2349 ±750.1 cal/mol
ΔS 10.3 cal/mol/deg
(h)
Time (min)
µcal/sec
ΔH/(kcal/mol)
Molar Ratio
Model: OneSites
Chi^2/DoF = 1167
N 0.397 ±0.133 Sites
K 2.67E4 ±6.84E3 M-1
ΔH -2230 ±889.6 cal/mol
ΔS 12.8 cal/mol/deg
(i)
Time (min)
µcal/sec
ΔH/(kcal/mol)
Molar Ratio
Model: OneSites
Chi^2/DoF = 2.347E5
N 654 ±7.07E6 Sites
K 41.1 ±4.51E5 M-1
ΔH -3994 ±4.648E5 cal/mol
ΔS -6.01 cal/mol/deg

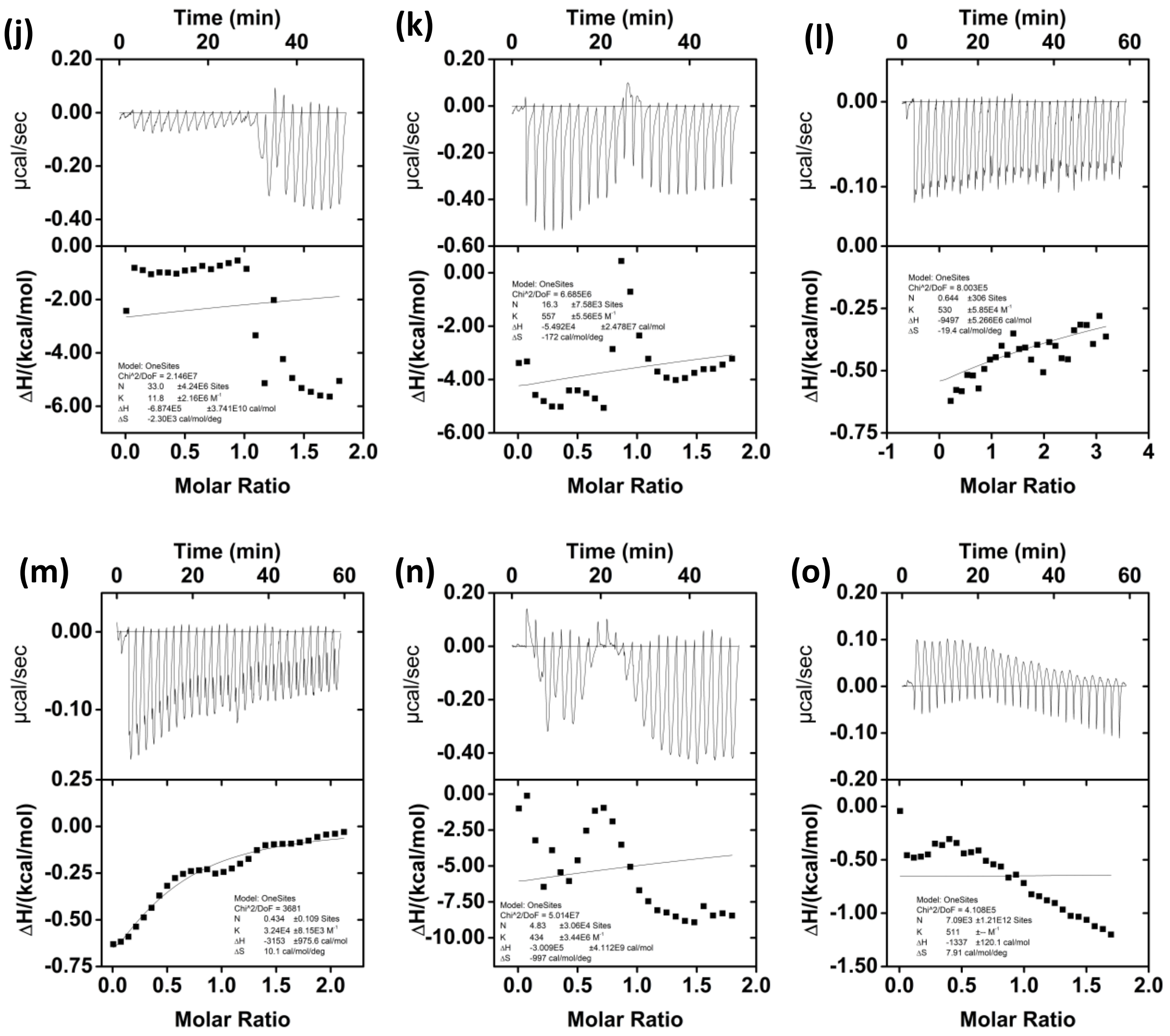

Supplementary Figure 14. ITC titration curves obtained at 298 K in 10 mM HEPES buffer (pH 7.5) for the binding of (a)–(e) lysozyme (0.2 mM) by **NP$_{\mathbf{A}}$**(lysozyme) (0.02 mM); (f) lysozyme (0.2 mM) by NINP (0.02 mM); (g) lysozyme (0.2 mM) by **NP$_{\mathbf{A}}$**(lysozyme) (0.02 mM); (h) BSA (0.2 mM) by **NP$_{\mathbf{A}}$**(lysozyme) (0.02 mM); (i) HRP (0.2 mM) by **NP$_{\mathbf{A}}$**(lysozyme) (0.02 mM); (j) α-amylase (0.2 mM) by **NP$_{\mathbf{A}}$**(lysozyme) (0.02 mM); (k) cytochrome C (0.2 mM) by **NP$_{\mathbf{A}}$**(lysozyme) (0.02 mM); (l) chymotrypsin (0.2 mM) by **NP$_{\mathbf{A}}$**(lysozyme) (0.02 mM); (m) OVA (0.2 mM) by **NP$_{\mathbf{A}}$**(lysozyme) (0.02 mM); (n) transferrin (0.2 mM) by **NP$_{\mathbf{A}}$**(lysozyme) (0.02 mM); (o) trypsin (0.2 mM) by **NP$_{\mathbf{A}}$**(lysozyme) (0.02 mM). The data correspond to entries 1–15, respectively, in Table 1. Experiments were performed in triplicates with the errors among the runs <5%.

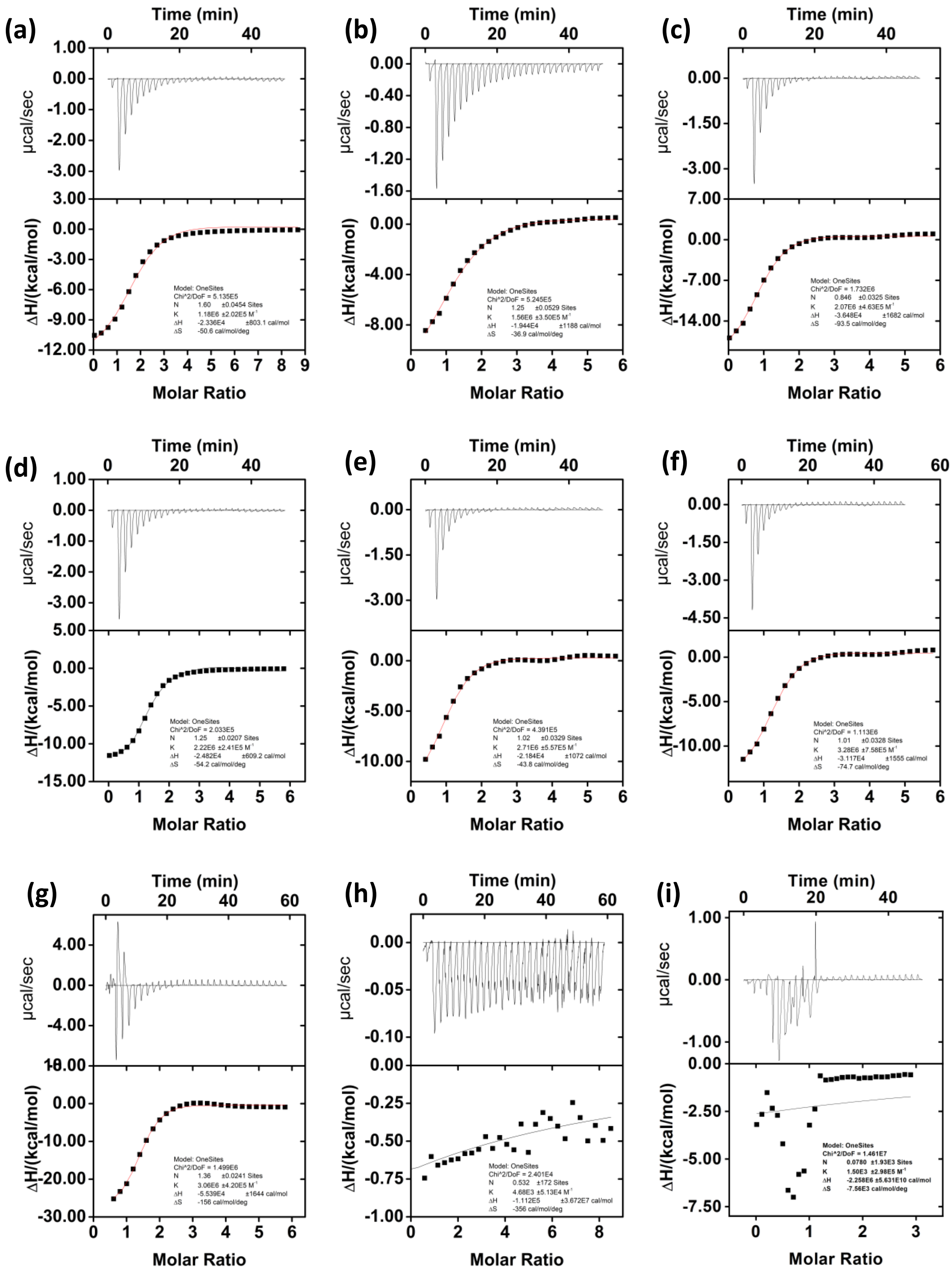

(a)
Time (min)
µcal/sec
ΔH/(kcal/mol)
Molar Ratio
Model: OneSites
Chi^2/DoF = 5.135E5
N 1.60 ±0.0454 Sites
K 1.18E6 ±2.02E5 M-1
ΔH -2.336E4 ±803.1 cal/mol
ΔS -50.6 cal/mol/deg
(b)
Time (min)
µcal/sec
ΔH/(kcal/mol)
Molar Ratio
Model: OneSites
Chi^2/DoF = 5.245E5
N 1.25 ±0.0529 Sites
K 1.56E6 ±3.50E5 M-1
ΔH -1.944E4 ±1188 cal/mol
ΔS -36.9 cal/mol/deg
(c)
Time (min)
µcal/sec
ΔH/(kcal/mol)
Molar Ratio
Model: OneSites
Chi^2/DoF = 1.732E6
N 0.846 ±0.0325 Sites
K 2.07E6 ±4.63E5 M-1
ΔH -3.648E4 ±1682 cal/mol
ΔS -93.5 cal/mol/deg
(d)
Time (min)
µcal/sec
ΔH/(kcal/mol)
Molar Ratio
Model: OneSites
Chi^2/DoF = 2.033E5
N 1.25 ±0.0207 Sites
K 2.22E6 ±2.41E5 M-1
ΔH -2.482E4 ±609.2 cal/mol
ΔS -54.2 cal/mol/deg
(e)
Time (min)
µcal/sec
ΔH/(kcal/mol)
Molar Ratio
Model: OneSites
Chi^2/DoF = 4.391E5
N 1.02 ±0.0329 Sites
K 2.71E6 ±5.57E5 M-1
ΔH -2.184E4 ±1072 cal/mol
ΔS -43.8 cal/mol/deg
(f)
Time (min)
µcal/sec
ΔH/(kcal/mol)
Molar Ratio
Model: OneSites
Chi^2/DoF = 1.113E6
N 1.01 ±0.0328 Sites
K 3.28E6 ±7.58E5 M-1
ΔH -3.117E4 ±1555 cal/mol
ΔS -74.7 cal/mol/deg
(g)
Time (min)
µcal/sec
ΔH/(kcal/mol)
Molar Ratio
Model: OneSites
Chi^2/DoF = 1.499E6
N 1.36 ±0.0241 Sites
K 3.06E6 ±4.20E5 M-1
ΔH -5.539E4 ±1644 cal/mol
ΔS -156 cal/mol/deg
(h)
Time (min)
µcal/sec
ΔH/(kcal/mol)
Molar Ratio
Model: OneSites
Chi^2/DoF = 2.401E4
N 0.532 ±172 Sites
K 4.68E3 ±5.13E4 M-1
ΔH -1.112E5 ±3.672E7 cal/mol
ΔS -356 cal/mol/deg
(i)
Time (min)
µcal/sec
ΔH/(kcal/mol)
Molar Ratio
Model: OneSites
Chi^2/DoF = 1.461E7
N 0.0780 ±1.93E3 Sites
K 1.50E3 ±2.98E5 M-1
ΔH -2.258E6 ±5.631E10 cal/mol
ΔS -7.56E3 cal/mol/deg

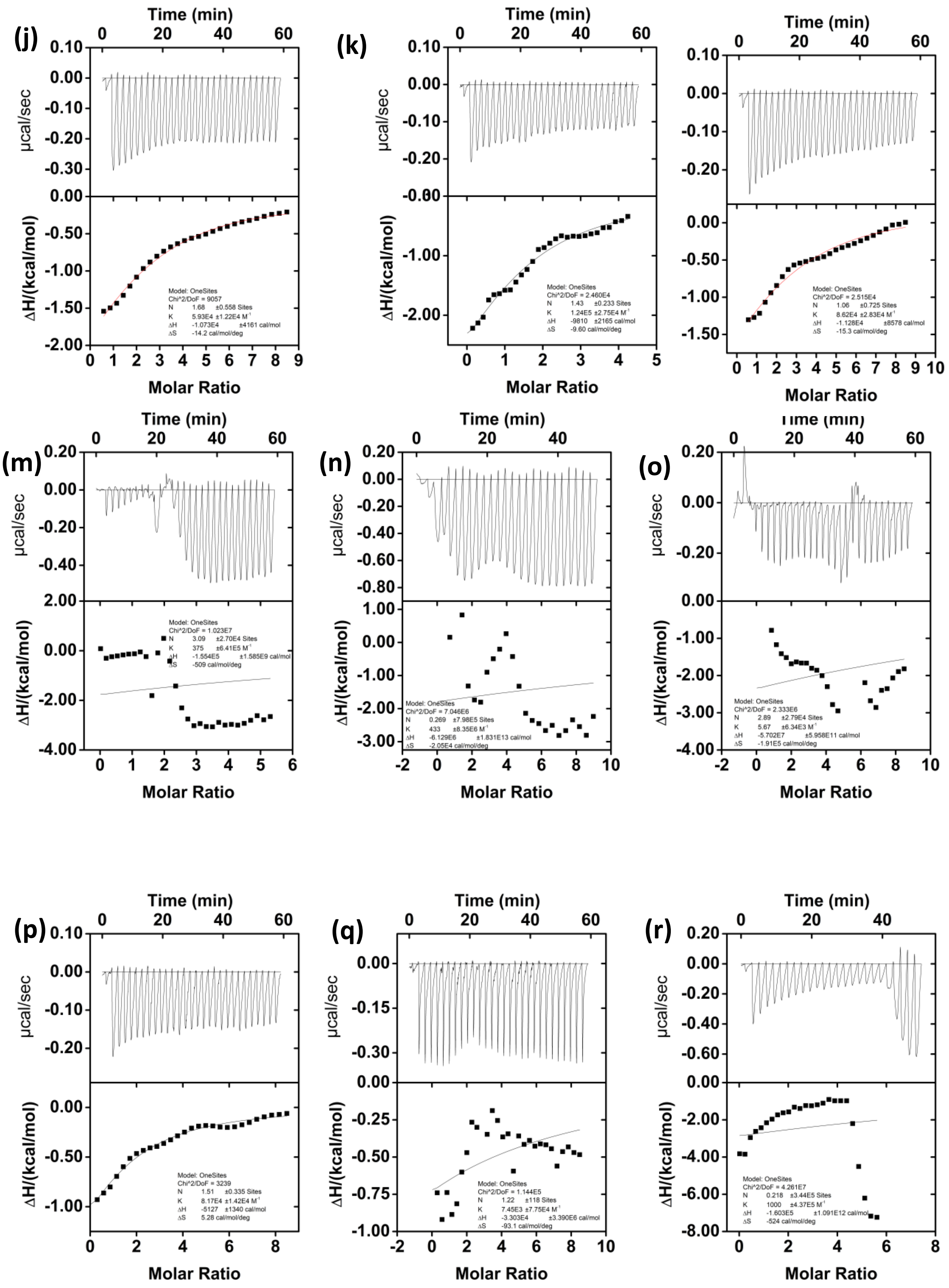

(j)
Time (min)
µcal/sec
ΔH/(kcal/mol)
Molar Ratio
Model: OneSites
Chi^2/DoF = 9057
N 1.68 ±0.558 Sites
K 5.93E4 ±1.22E4 M^-1
ΔH -1.073E4 ±4161 cal/mol
ΔS -14.2 cal/mol/deg
(k)
Time (min)
µcal/sec
ΔH/(kcal/mol)
Molar Ratio
Model: OneSites
Chi^2/DoF = 2.460E4
N 1.43 ±0.233 Sites
K 1.24E5 ±2.75E4 M^-1
ΔH -9810 ±2165 cal/mol
ΔS -9.60 cal/mol/deg
Time (min)
Molar Ratio
Model: OneSites
Chi^2/DoF = 2.515E4
N 1.06 ±0.725 Sites
K 8.62E4 ±2.83E4 M^-1
ΔH -1.128E4 ±8578 cal/mol
ΔS -15.3 cal/mol/deg
(m)
Time (min)
µcal/sec
ΔH/(kcal/mol)
Molar Ratio
Model: OneSites
Chi^2/DoF = 1.023E7
N 3.09 ±2.70E4 Sites
K 375 ±6.41E5 M^-1
ΔH -1.554E5 ±1.585E9 cal/mol
ΔS -509 cal/mol/deg
(n)
Time (min)
µcal/sec
ΔH/(kcal/mol)
Molar Ratio
Model: OneSites
Chi^2/DoF = 7.046E6
N 0.269 ±7.98E5 Sites
K 433 ±8.35E6 M^-1
ΔH -6.129E6 ±1.831E13 cal/mol
ΔS -2.05E4 cal/mol/deg
(o)
µcal/sec
ΔH/(kcal/mol)
Molar Ratio
Model: OneSites
Chi^2/DoF = 2.333E6
N 2.89 ±2.79E4 Sites
K 5.67 ±6.34E3 M^-1
ΔH -5.702E7 ±5.958E11 cal/mol
ΔS -1.91E5 cal/mol/deg
(p)
Time (min)
µcal/sec
ΔH/(kcal/mol)
Molar Ratio
Model: OneSites
Chi^2/DoF = 3239
N 1.51 ±0.335 Sites
K 8.17E4 ±1.42E4 M^-1
ΔH -5127 ±1340 cal/mol
ΔS 5.28 cal/mol/deg
(q)
Time (min)
µcal/sec
ΔH/(kcal/mol)
Molar Ratio
Model: OneSites
Chi^2/DoF = 1.144E5
N 1.22 ±118 Sites
K 7.45E3 ±7.75E4 M^-1
ΔH -3.303E4 ±3.390E6 cal/mol
ΔS -93.1 cal/mol/deg
(r)
Time (min)
µcal/sec
ΔH/(kcal/mol)
Molar Ratio
Model: OneSites
Chi^2/DoF = 4.261E7
N 0.218 ±3.44E5 Sites
K 1000 ±4.37E5 M^-1
ΔH -1.603E5 ±1.091E12 cal/mol
ΔS -524 cal/mol/deg

Supplementary Figure 15. ITC titration curves obtained at 298 K in 10 mM HEPES buffer (pH 7.4) for the binding of (a)–(g) lysozyme (0.2 mM) by $\mathbf{NP_B}$(lysozyme) (0.005 mM); (h) Lysozyme (0.2 mM) by NINP (0.01 mM); (i) Lysozyme (0.2 mM) by NINP (0.01 mM); (j) Lysozyme (0.2 mM) by $\mathbf{NP_B}$(lysozyme) (0.005 mM); (k) BSA (0.2 mM) by $\mathbf{NP_B}$(lysozyme) (0.005 mM); (l) HRP (0.2 mM) by $\mathbf{NP_B}$(lysozyme) (0.005 mM); (m) α-amylase (0.2 mM) by $\mathbf{NP_B}$(lysozyme) (0.005 mM); (n) cytochrome C (0.2 mM) by NP_A(lysozyme) (0.005 mM); (o) chymotrypsin (0.2 mM) by $\mathbf{NP_B}$(lysozyme) (0.005 mM); (p) OVA (0.2 mM) by $\mathbf{NP_B}$(lysozyme) (0.005 mM); (q) transferrin (0.2 mM) by $\mathbf{NP_B}$(lysozyme) (0.005 mM); (r) trypsin (0.2 mM) by $\mathbf{NP_B}$(lysozyme) (0.005 mM). The data correspond to entries 1–18, respectively, in Table 2. Experiments were performed in triplicates with the errors among the runs <5%.

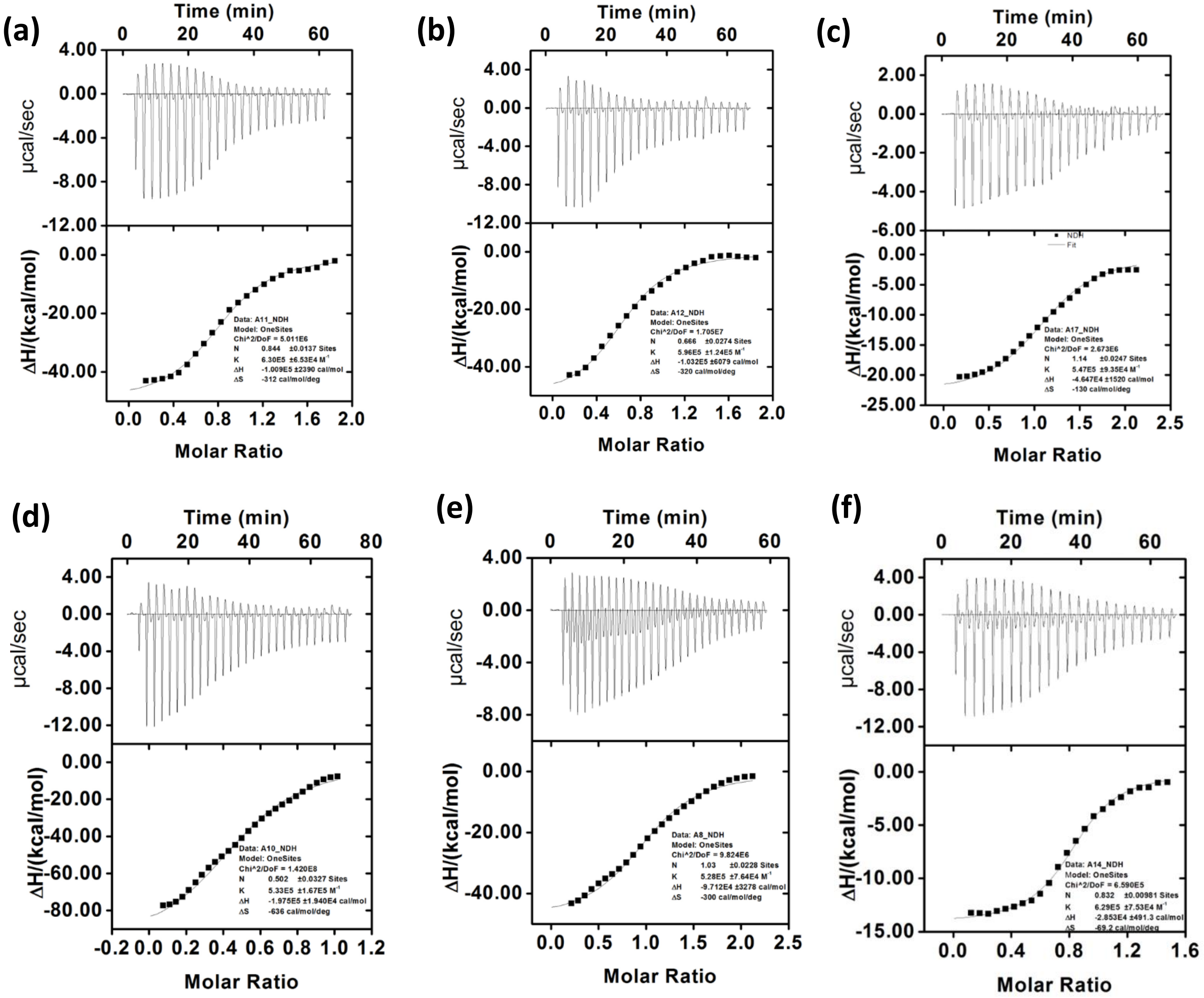

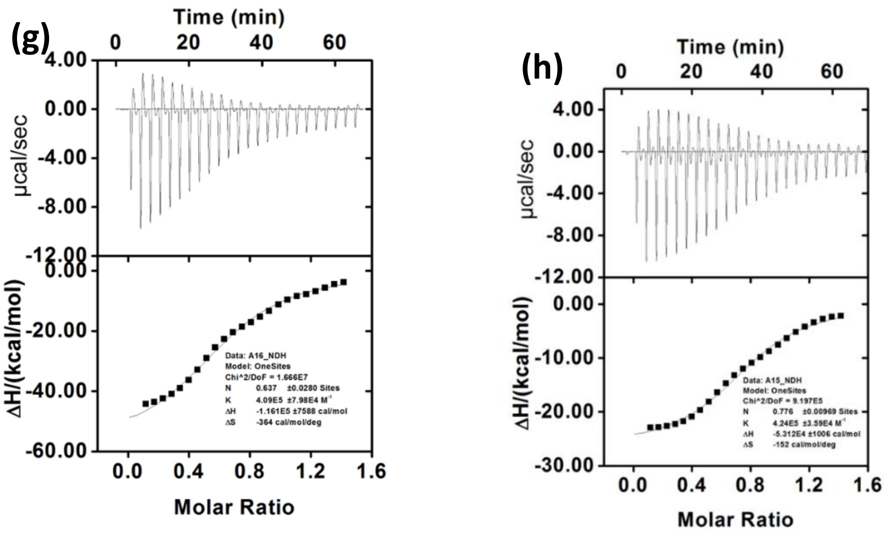

Supplementary Figure 16. ITC titration curves obtained at 298 K in 10 mM HEPES buffer (pH 7.5) for the binding of lysozyme (0.2 mM) by **NP$_{\mathbf{A}}$**(lysozyme) (0.02 mM) in presence of (a) BSA (0.4 mM); (b) HRP (0.4 mM); (c) α-amylase (0.4 mM); (d) cytochrome C (0.4 mM); (e) chymotrypsin (0.4 mM); (f) OVA (0.4 mM); (g) transferrin (0.4 mM); (h) trypsin (0.4 mM). The data correspond to entries 1–8, respectively, in Supplementary Table 2. Experiments were performed in triplicates with the errors among the runs <5%.

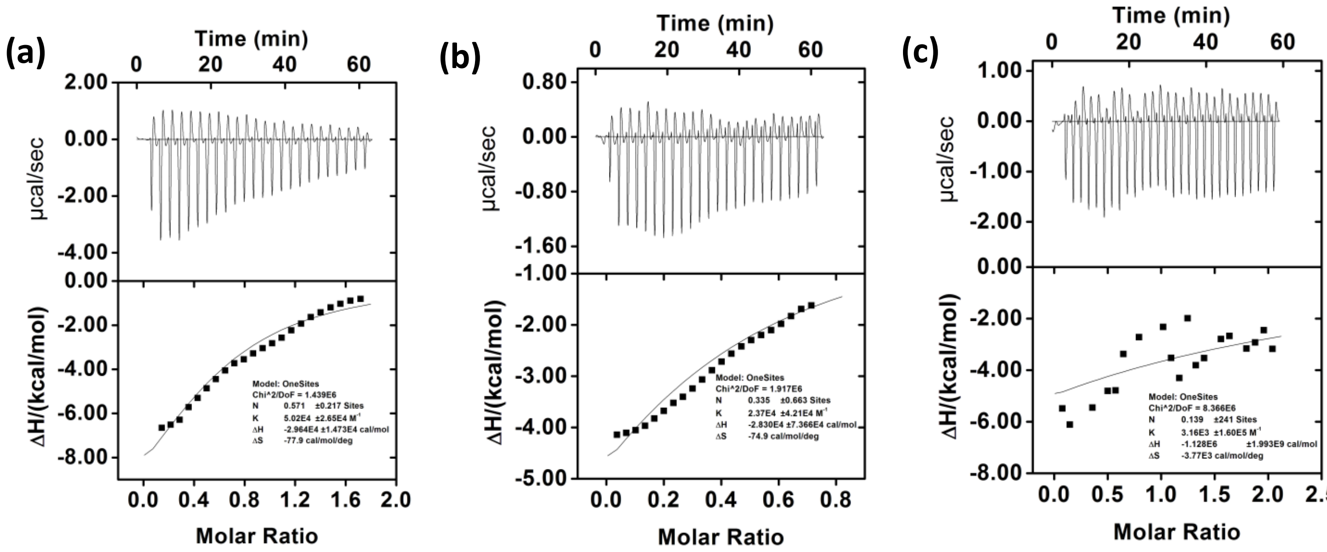

Supplementary Figure 17. ITC titration curves obtained at 298 K in 10 mM HEPES buffer (pH 7.5) for the binding of lysozyme (0.2 mM) by **NP$_{\mathbf{A}}$**(lysozyme) (0.02 mM) in presence of 3-amino-1-propanol at 0.02 mM (a), 0.05 mM (b), and 0.10 mM (c), respectively.

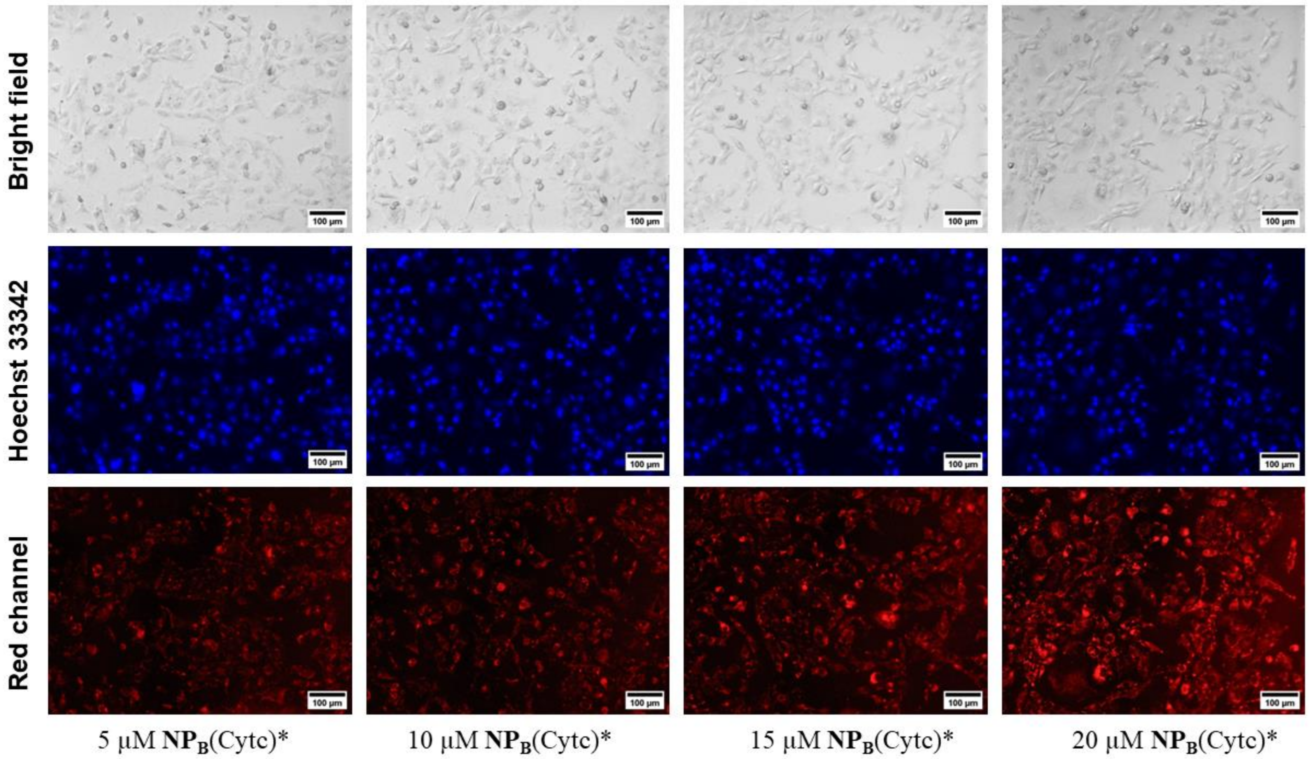

Supplementary Figure 18. Concentration-dependent cellular uptake of $\mathbf{NP_B}$(Cytc)* monitored by fluorescence imaging. MDA-MB-231 cells were incubated with 5–20 µM $\mathbf{NP_B}$(Cytc)* for 1 h. Hoechst 33342 was used to stain the nucleus. The experiments were repeated 3 times with similar results. (Scale bar 100 µm)**.**

(a)

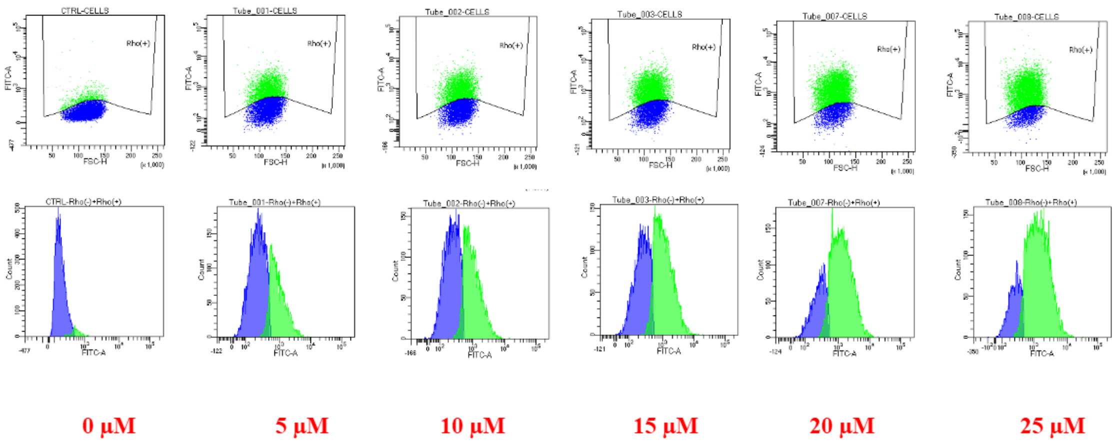

(b)

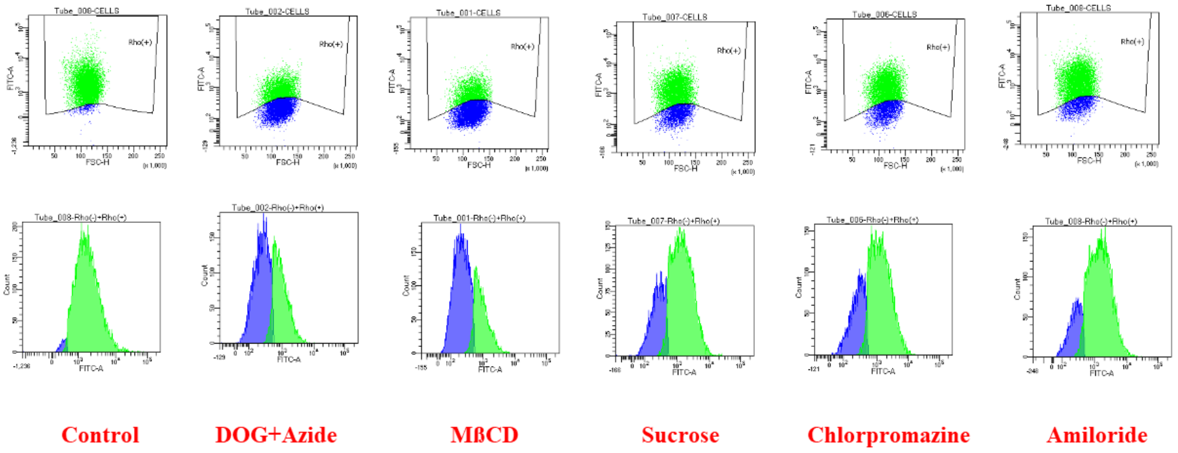

Supplementary Figure 19. Cellular uptake experiments. (a) Concentration-dependent cellular uptake of **NP$_{\mathbf{B}}$**(Cytc)***** measured by FACS. Cells were incubated with 0–25 µM of **NP$_{\mathbf{B}}$**(Cytc)***** for 1 h before FACS measurements. The experiments were repeated 3 times with similar results. (b) Cellular uptake of **NP$_{\mathbf{B}}$**(Cytc)***** in the presence of different endocytosis inhibitor measured by FACS. Cells were incubated with 20 µM of **NP$_{\mathbf{B}}$**(Cytc)***** for 1 h after treated with methyl-β-cyclodextrin (5.0 mM for 30 min), sucrose (0.45 M for 10 min), chlorpromazine (20 µg/mL for 45 min), amiloride (1 mM for 30 min), sodium azide/2-deoxy-D-glucose (10 & 6 mM, respectively, for 1 h). The experiments were repeated 3 times with similar results.

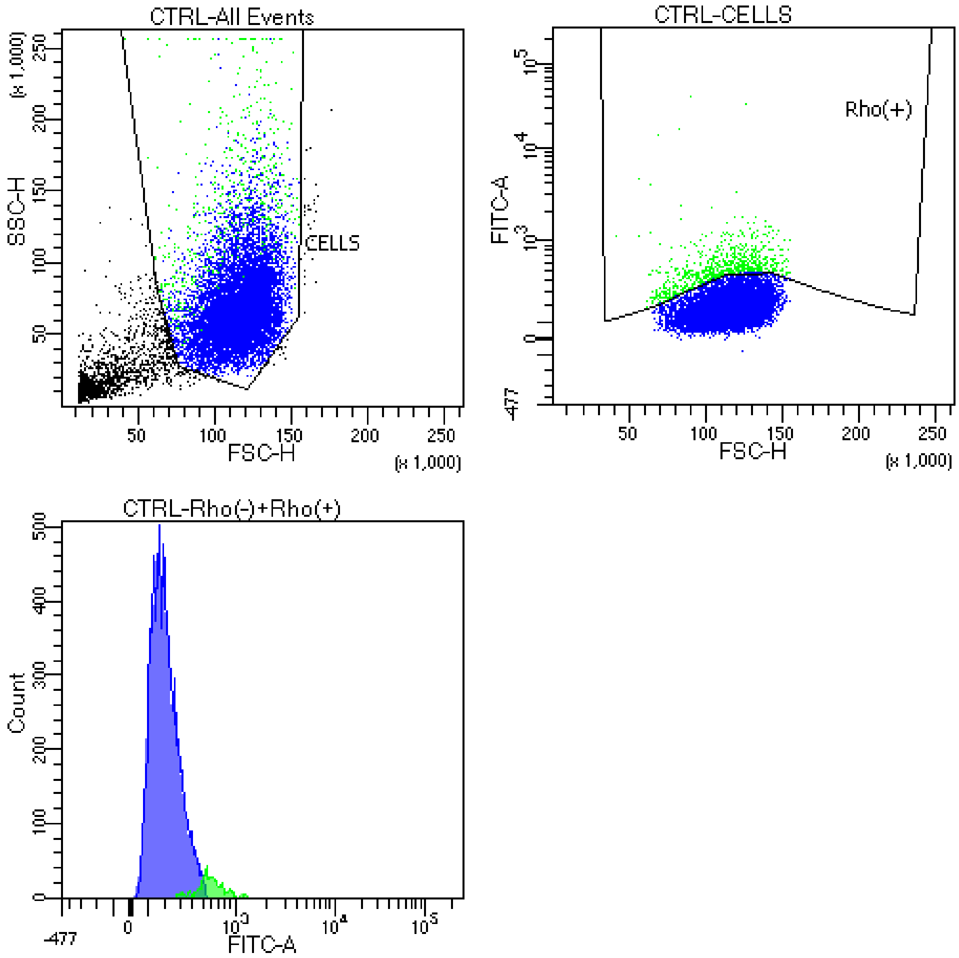

Supplementary Figure 20. Flow cytometry gating strategy for the cellular uptake experiments in Supplementary Figure 19.

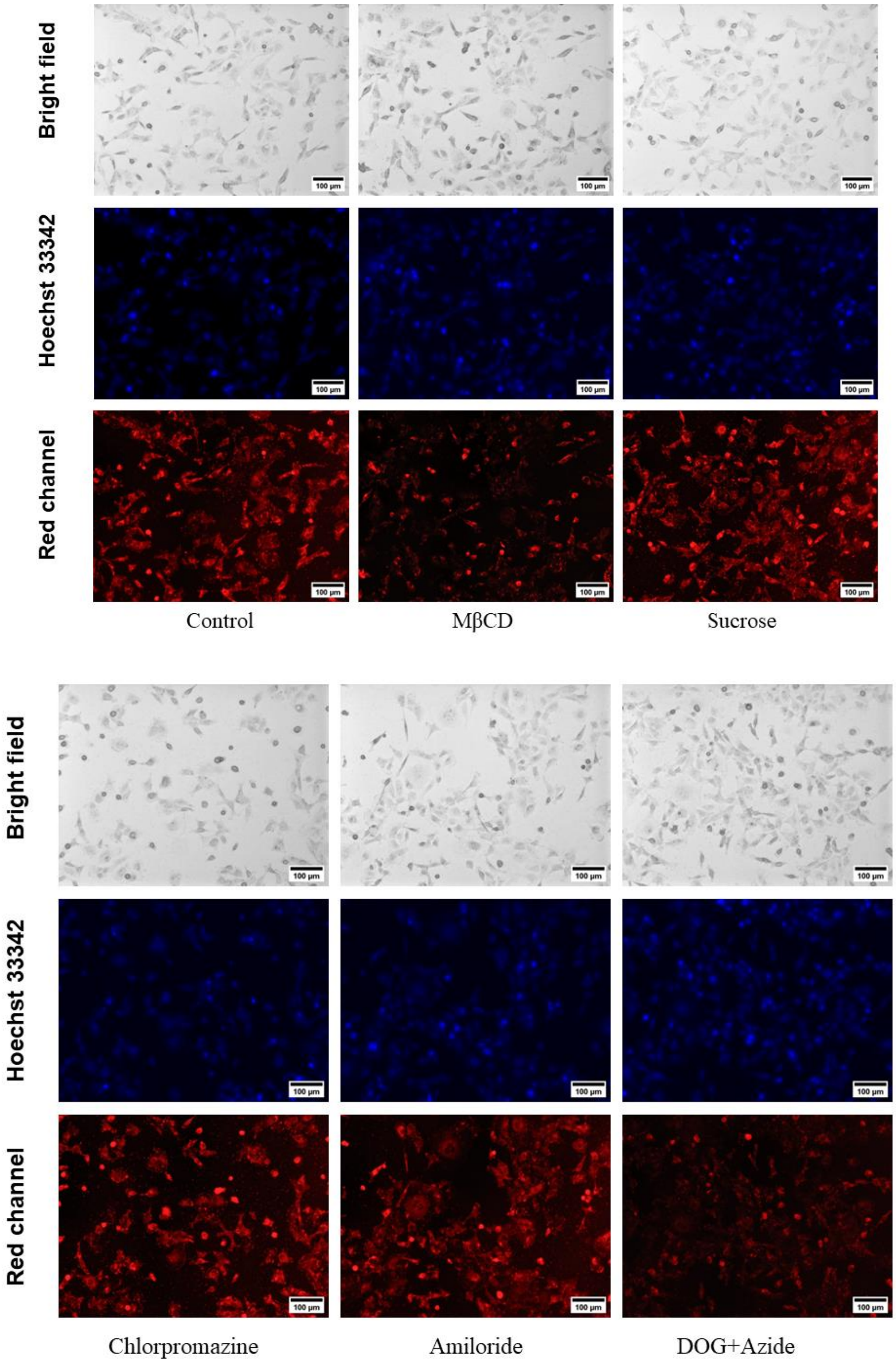

Supplementary Figure 21. Fluorescence images of MDA-MB-231 cells treated with 20 µM **NP$_B$**(Cytc)* and those in addition to different endocytosis inhibitors. The experiments were repeated 3 times with similar results. (Scale bar 100 µm).

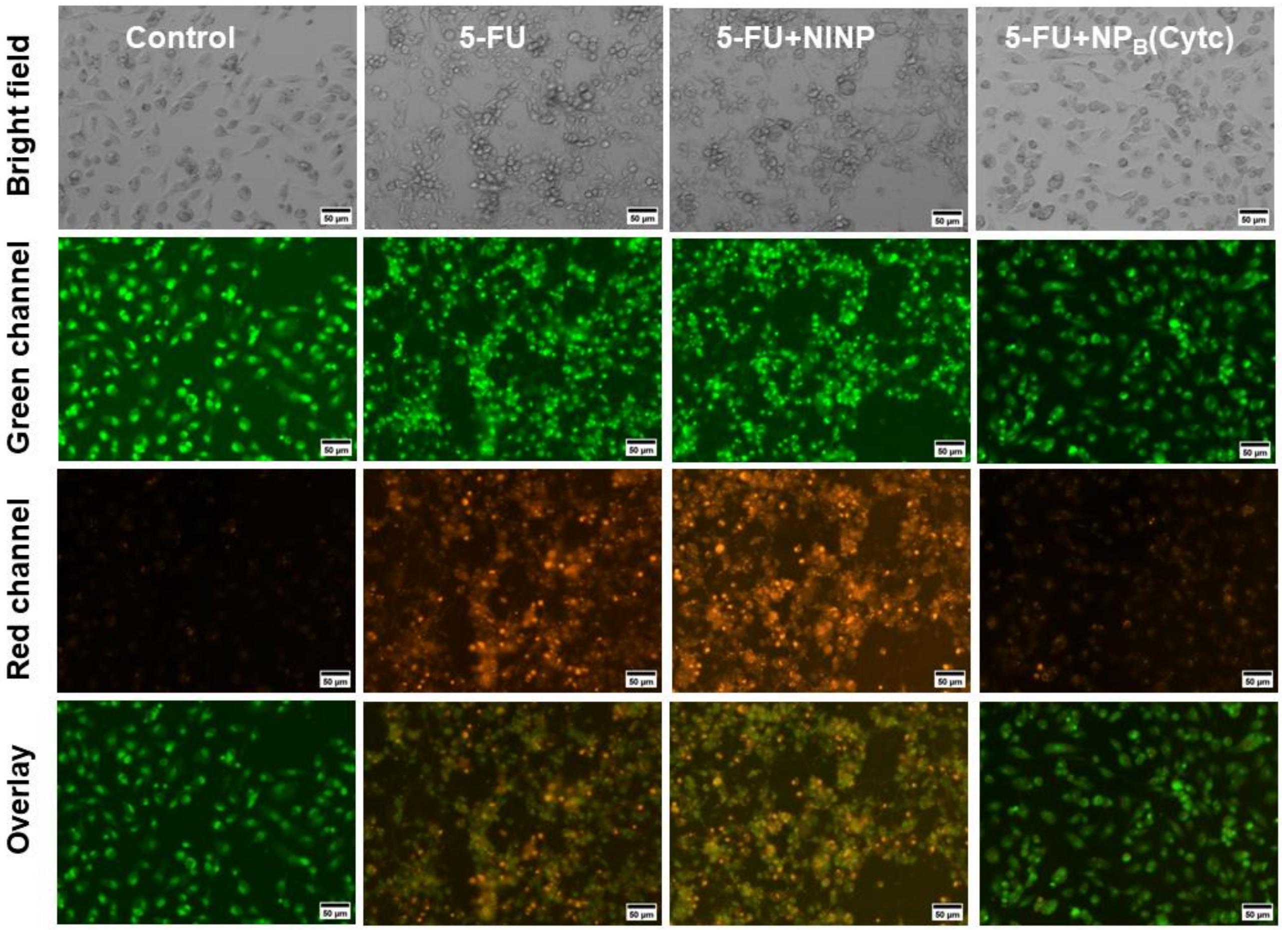

Supplementary Figure 22. Visualization of apoptotic MDA-MB-231 cells by fluorescence imaging after AO/EtBr staining. The experiments were repeated 3 times with similar results (Scale bar 50 µm)**.**

(a)

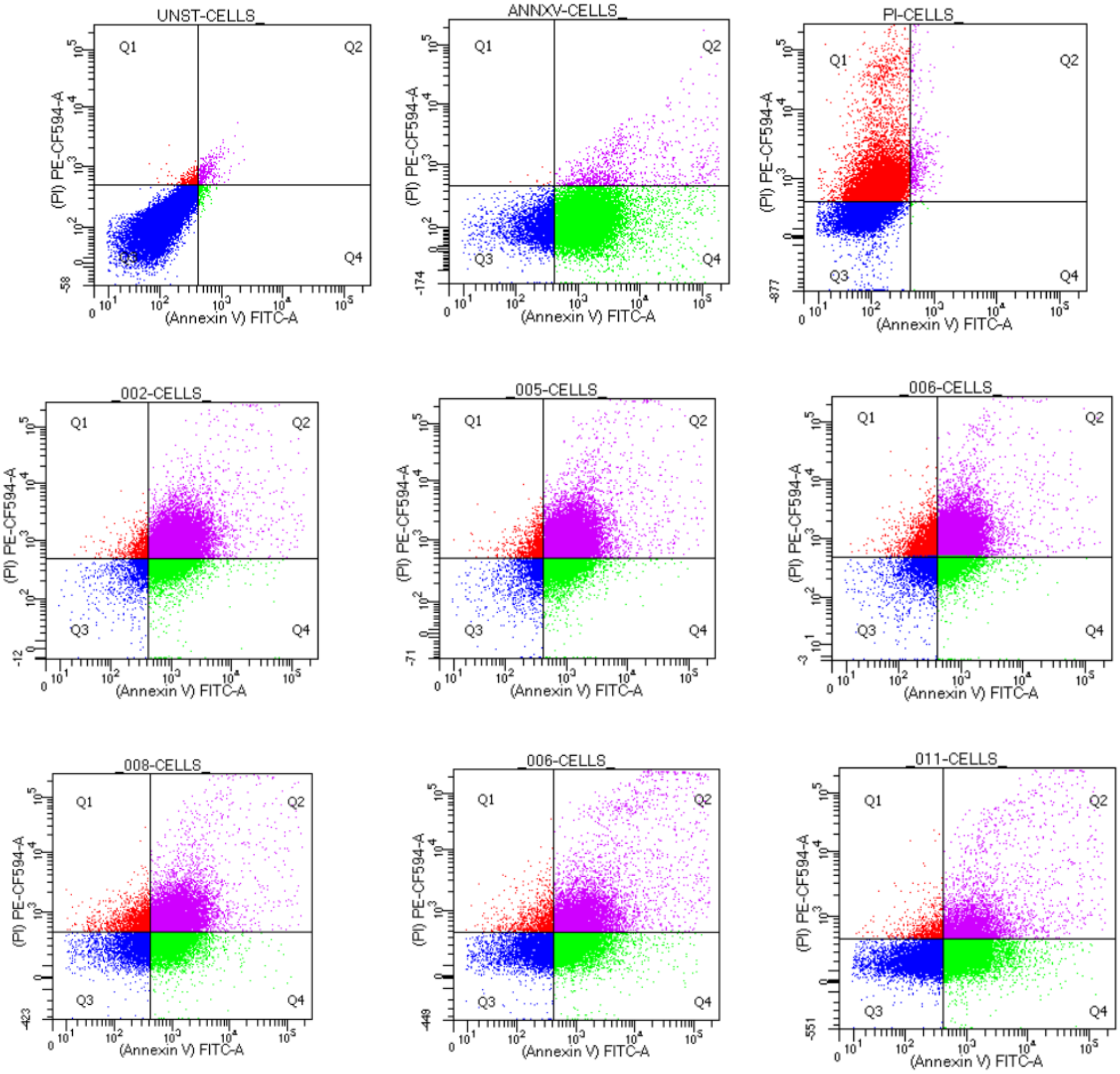

60μM 5-FU+100μM NP_B(cyto-C) **40μM 5-FU+ 100μM NP_B(cyto-C)** **20μM 5-FU+ 100μM NP_B(cyto-C)**

(b)

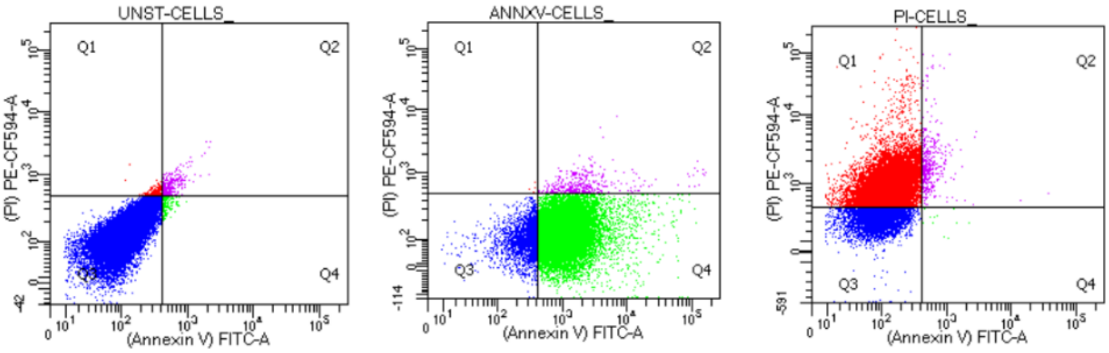

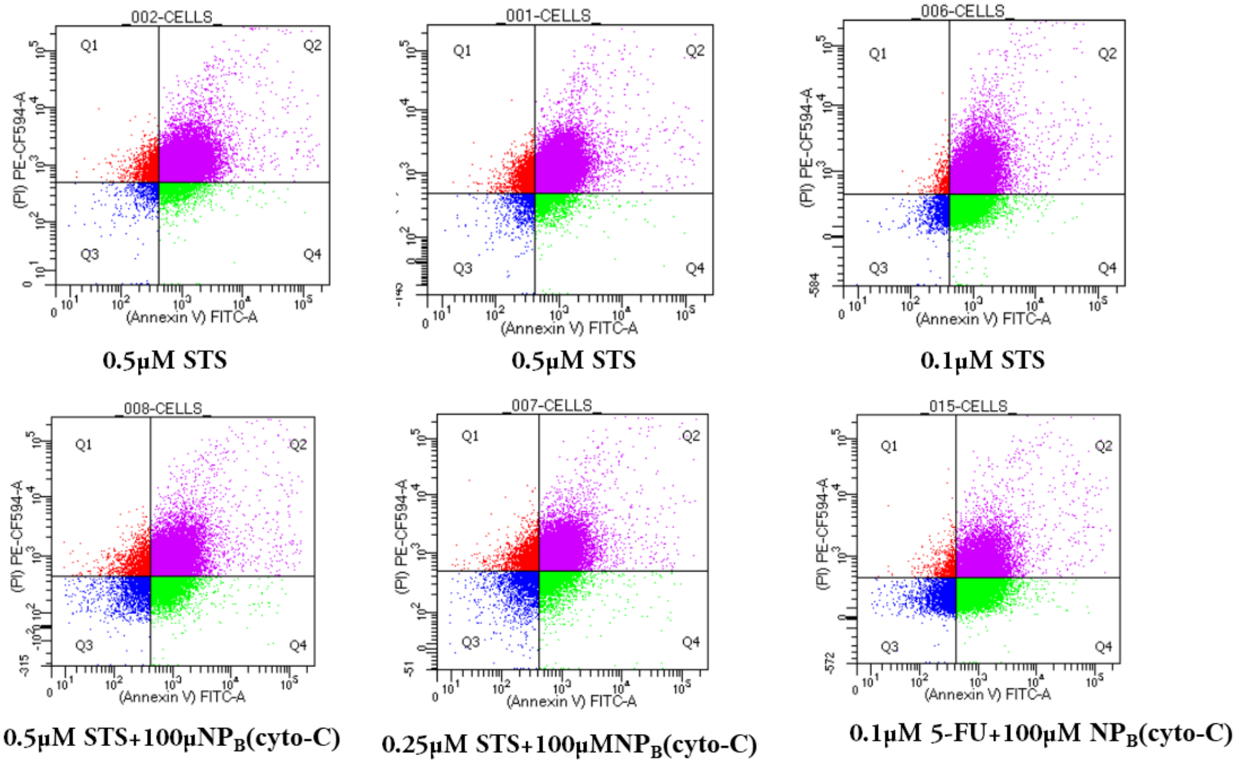

Supplementary Figure 23. Apoptosis Studies. (a) Apoptosis of MDA-MB-231 cells after 5-fluorouracil treatment measured by FACS. The experiments were repeated 3 times with similar results. (b) Apoptosis of MDA-MB-231 cells after STS treatment measured by FACS. The experiments were repeated 3 times with similar results.

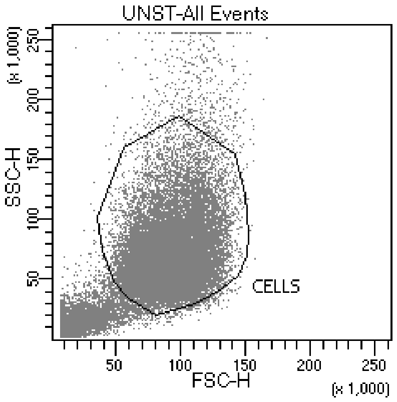
UNST-All Events
SSC-H
(x 1,000)
FSC-H
CELLS
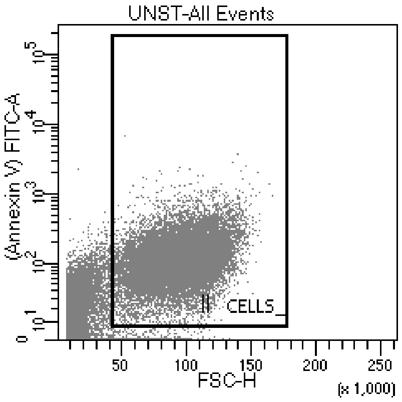
UNST-All Events
(Annexin V) FITC-A
FSC-H
(x 1,000)
CELLS_
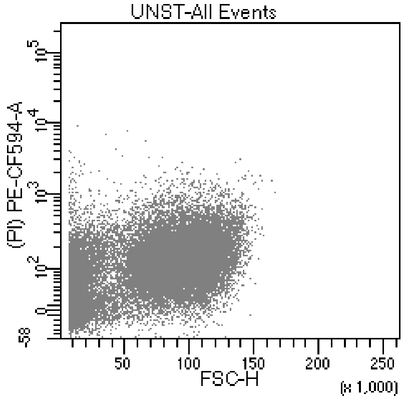
UNST-All Events
(PI) PE-CF594-A
FSC-H
(x 1,000)
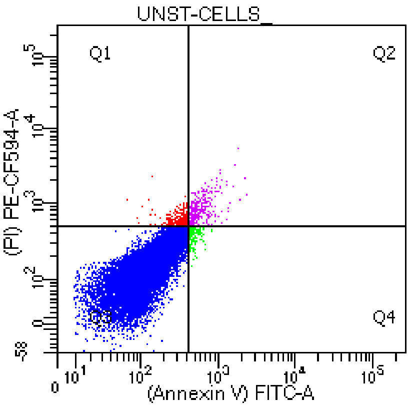
UNST-CELLS_
(PI) PE-CF594-A
(Annexin V) FITC-A
Q1
Q2
Q4
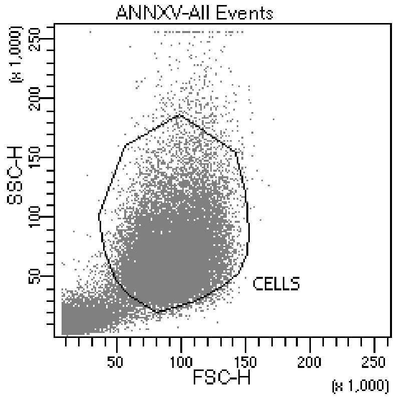
ANNXV-All Events
SSC-H
(x 1,000)
FSC-H
CELLS
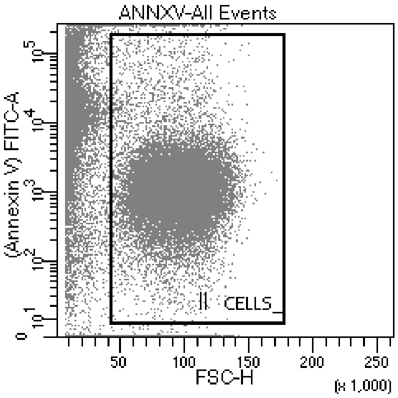
ANNXV-All Events
(Annexin V) FITC-A
FSC-H
(x 1,000)
CELLS_
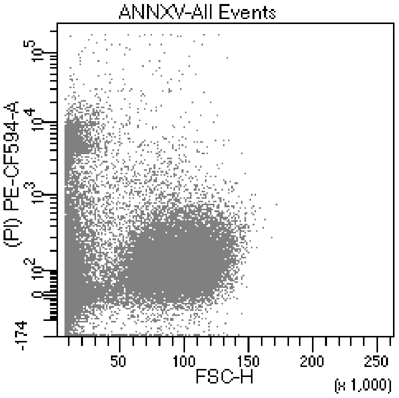
ANNXV-All Events
(PI) PE-CF594-A
FSC-H
(x 1,000)
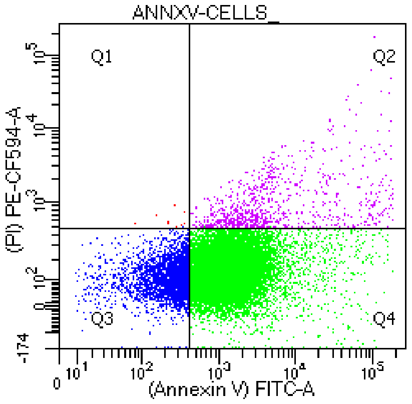
ANNXV-CELLS_
(PI) PE-CF594-A
(Annexin V) FITC-A
Q1
Q2
Q3
Q4

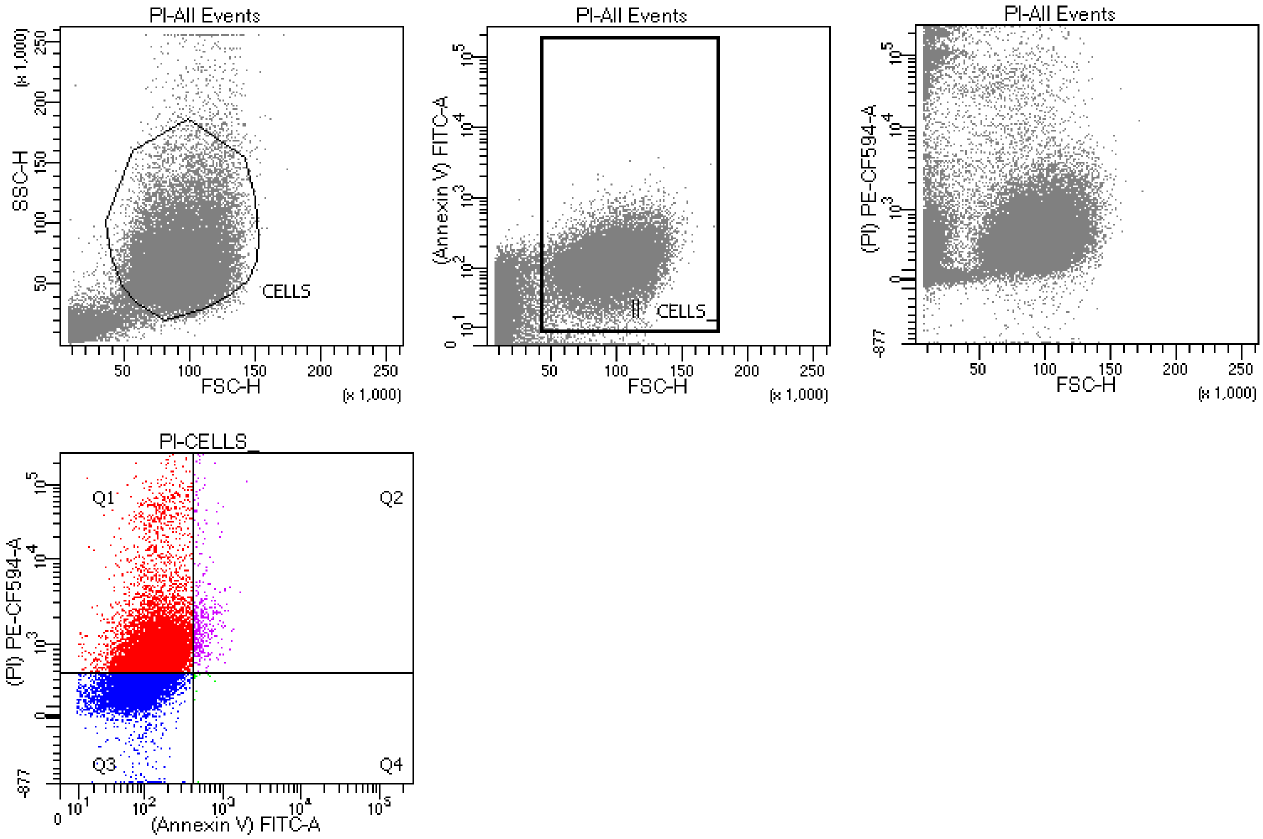

Supplementary Figure 24. Flow cytometry gating strategy for the apoptosis experiments in Supplementary Figure 23.

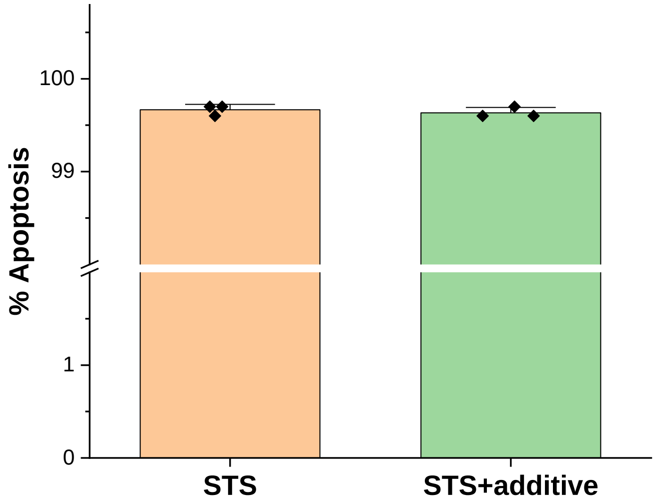

Supplementary Figure 25. Comparison of percent apoptosis induced by 0.5 µM saurosporine (STS) and 0.5 µM STS + additive determined by FACS. [Additive] = [**NP$_B$**(lysozyme)] = 100 µM in the experiment. Data are presented as the mean ± SE, n = 3 independent biological samples.

Statistical Analysis of Apoptosis Data

The experiment was designed as Randomized Complete Block Design (RCBD): 3 different batches of cells served as blocking factors, with treatment and dosage combinations randomized within each block; each treatment and dosage combinations within each block were measured for the % apoptosis independently (3 subsamples were taken and averaged for each block). A liner mixed model was applied to model % apoptosis for STS and 5FU separately. The data was modeled as a function of an overall mean, a random blocking factor, fixed effects of treatment, dosages, and treatment by dosage interaction, and a random residual. Contrasts with 2-sided *t*-test based on the linear mixed model were conducted to compare treatment against untreated within each dose. A difference was considered statistically significant if the *p*-value of the difference was less than 0.05 and the 95% confidence interval doesn't include 0. All data analysis and contrasts were made in R 4.3.1 [R Core Team (2023). R: A Language and Environment for Statistical Computing. R Foundation for Statistical Computing, Vienna, Austria. https://www.R-project.org/.], using lme4 1.1-33 [Douglas Bates, Martin Maechler, Ben Bolker, Steve Walker (2015). Fitting Linear Mixed-Effects Models Using lme4. J. Stat. Softw., 67(1), 1-48. doi:10.18637/jss.v067.i01] and emmeans 1.8.7 [Lenth R (2023). emmeans: Estimated Marginal Means, aka Least-Squares Means. R package version 1.8.7, https://CRAN.R-project.org/package=emmeans].

Supplementary Table 11. Statistical comparison between differently treated cells.

Exp	Treatment	Dose (μM)	Estimated ± SE		Difference	SED	*t*-statistics	DF	*p*-value	95% confidence interval of difference	
			Treated	Untreated						Lower	Upper
STS	pep-A	0.1	20.000±7.420	49.533±7.080	-29.533*	3.141	-9.402	2.583	0.009	-42.210	-16.857
STS	$\mathbf{NP_B}$(Cytc)	0.1	30.333±7.420	49.533±7.080	-19.200*	3.141	-6.113	2.583	0.022	-31.876	-6.524
STS	NINP	0.1	49.000±7.420	49.533±7.080	-0.533	3.141	-0.170	2.583	0.880	-13.210	12.143
STS	$\mathbf{NP_B}$(Cytc)	0.25	42.000±7.420	57.000±7.420	-15.000*	3.847	-3.899	2.583	0.039	-28.439	-1.561
STS	$\mathbf{NP_B}$(Cytc)	0.5	45.000±7.420	60.600±7.420	-15.600*	3.847	-4.055	2.583	0.036	-29.039	-2.161
5FU	$\mathbf{NP_B}$(Cytc)	20	17.667±4.956	39.833±4.665	-22.167*	2.893	-7.661	3.091	0.009	-32.680	-11.654
5FU	NINP	20	37.667±4.956	39.833±4.665	-2.167	2.893	-0.749	3.091	0.519	-12.680	8.346
5FU	$\mathbf{NP_B}$(Cytc)	40	31.333±4.956	49.000±4.665	-17.667*	3.341	-5.288	3.091	0.012	-28.125	-7.208
5FU	$\mathbf{NP_B}$(Cytc)	60	44.000±4.956	58.333±4.665	-14.333*	3.341	-4.290	3.091	0.022	-24.792	-3.875

*Differences between treated and untreated are statistically significant ($P < 0.05$ from two-sided *t*-test based on linear mixed model and 95% confidence interval doesn't contain 0).

Supplementary Table 12. Fixed effects on % Apoptosis for each experiment.

Exp	Effect	df[a]	*F*-value	*p*-value[b]
STS	Treatment	3, 20	43.939	<0.001
STS	Dosage	2, 20	15.310	<0.001
STS	Treatment X Dosage	2, 20	0.447	0.646
5FU	Treatment	2, 20	50.525	<0.001
5FU	Dosage	2, 20	51.857	<0.001
5FU	Treatment X Dosage	2, 20	1.611	0.232

[a] df, Numerator degrees of freedom, denominator degrees of freedom. [b] *F*-test considered significant if *p*-value was less than 0.05.

Supplementary Table 13. Random effects of block and residual on % apoptosis for each experiment.

Exp	Effect	Variance	Standard Deviation
STS	Block	142.99	11.958
STS	Residual	22.199	4.712
5FU	Block	56.928	7.545
5FU	Residual	16.744	4.092

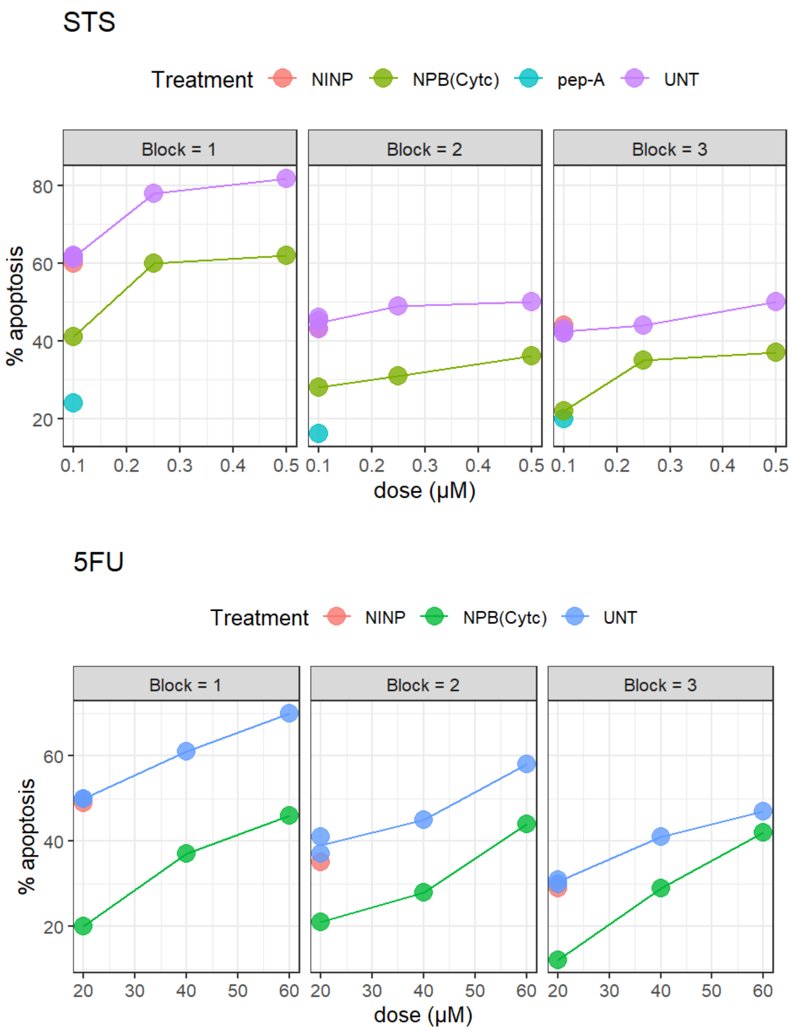

Supplementary Figure 26. Scatter plots for raw data by experiment.

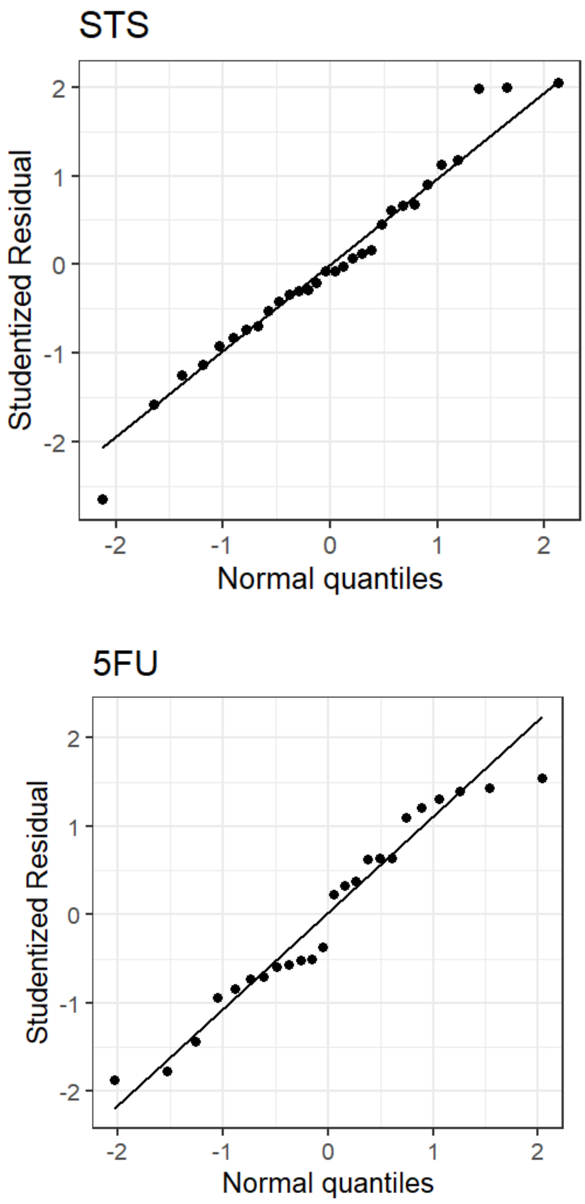

Supplementary Figure 27. Q-Q plots showing residuals from model fitting for each experiment. Data meets normality assumptions, and no outliers were detected.

^{1}H and ^{13}C NMR spectra of key compounds

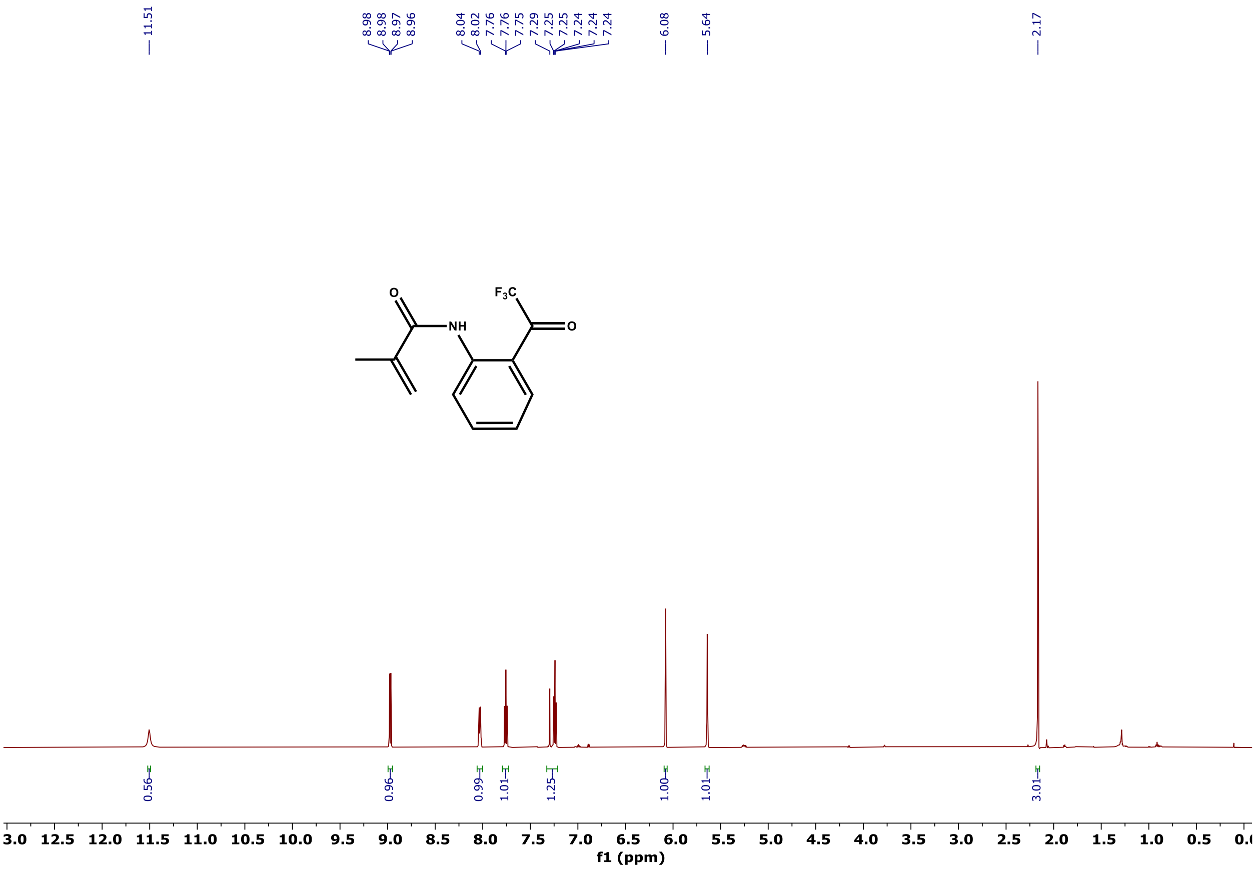

Supplementary Figure 28. ^{1}H NMR spectrum of compound **1a** in $CDCl_3$.

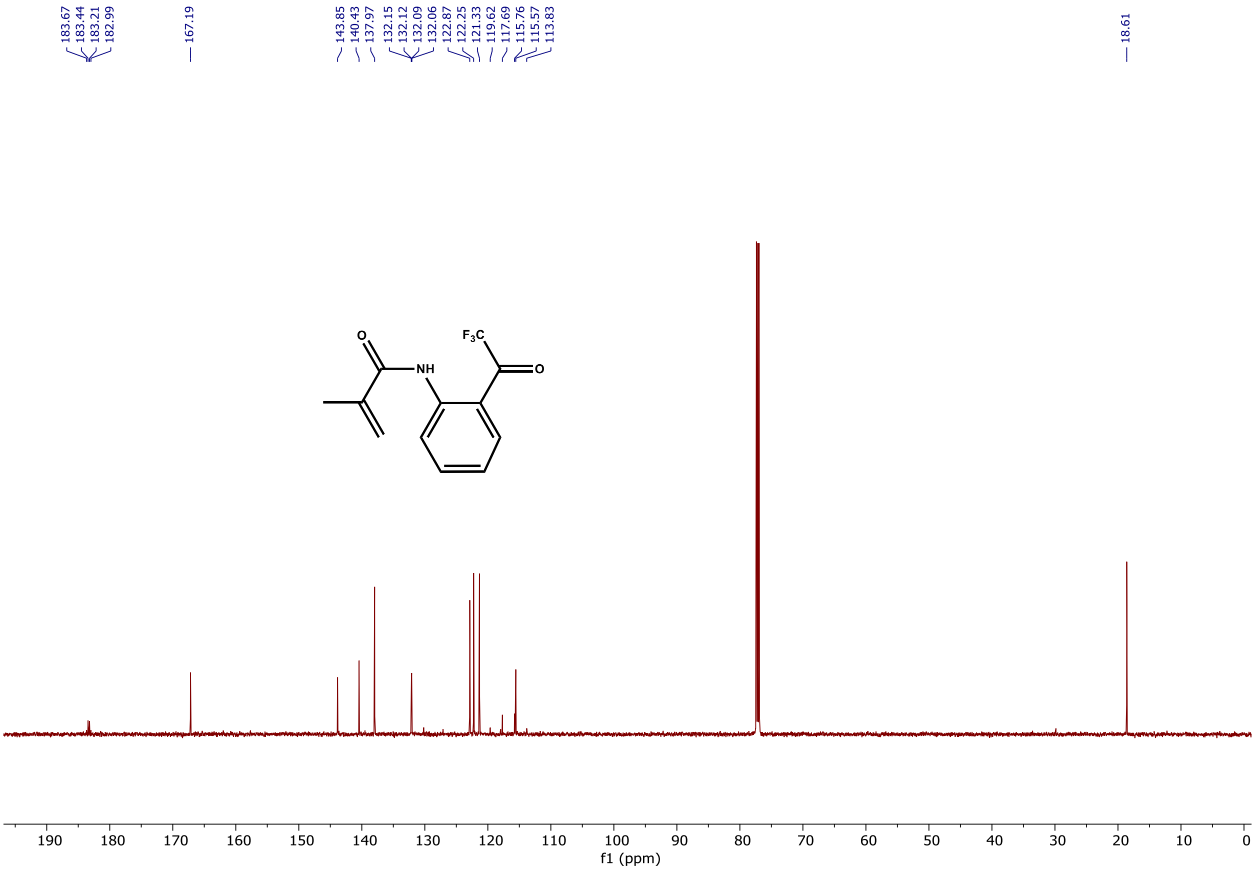

Supplementary Figure 29. ^{13}C NMR spectrum of compound **1a** in $CDCl_3$.

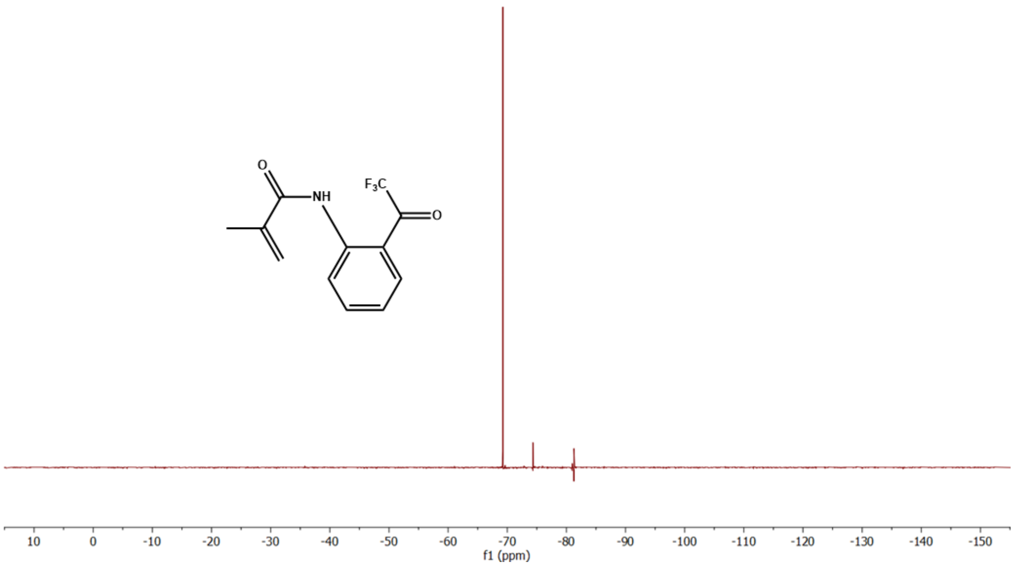

Supplementary Figure 30. ^{19}F NMR spectrum of compound **1a** in $CDCl_3$.

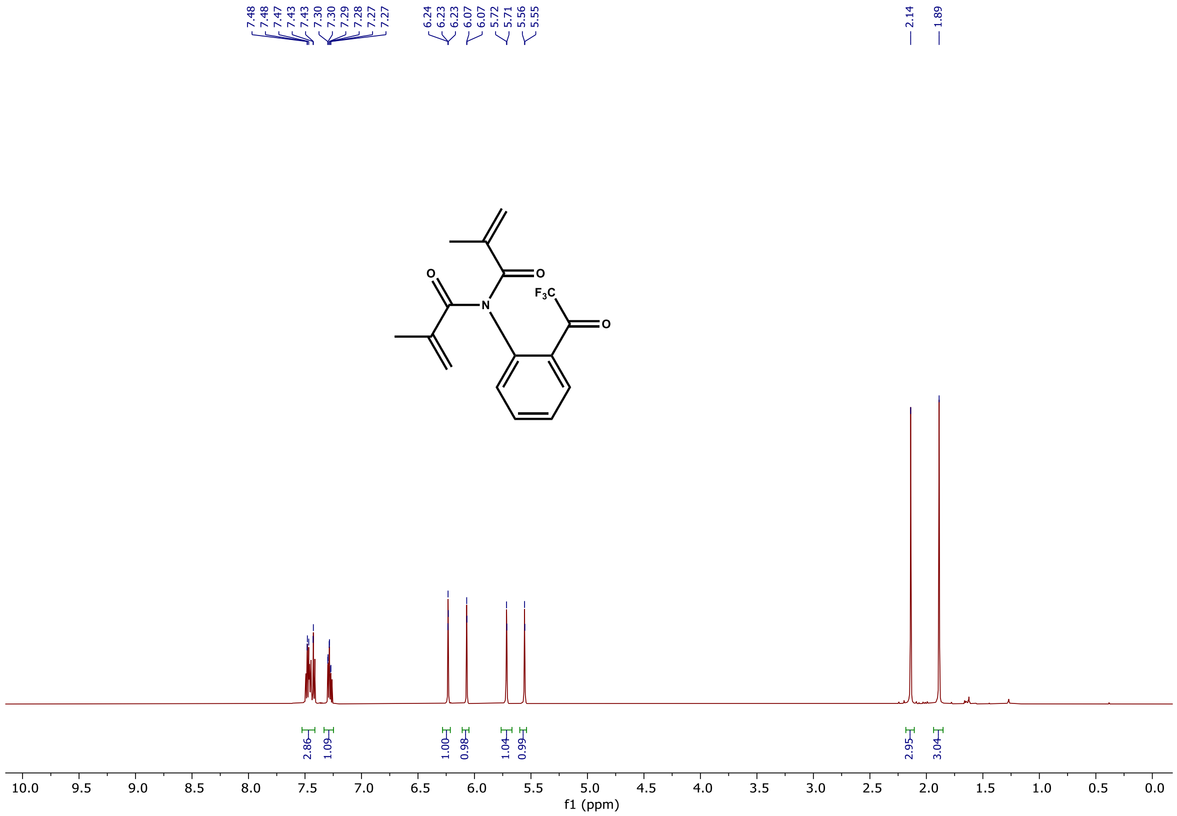

Supplementary Figure 31. ^{1}H NMR spectrum of compound **1b** in $CDCl_3$.

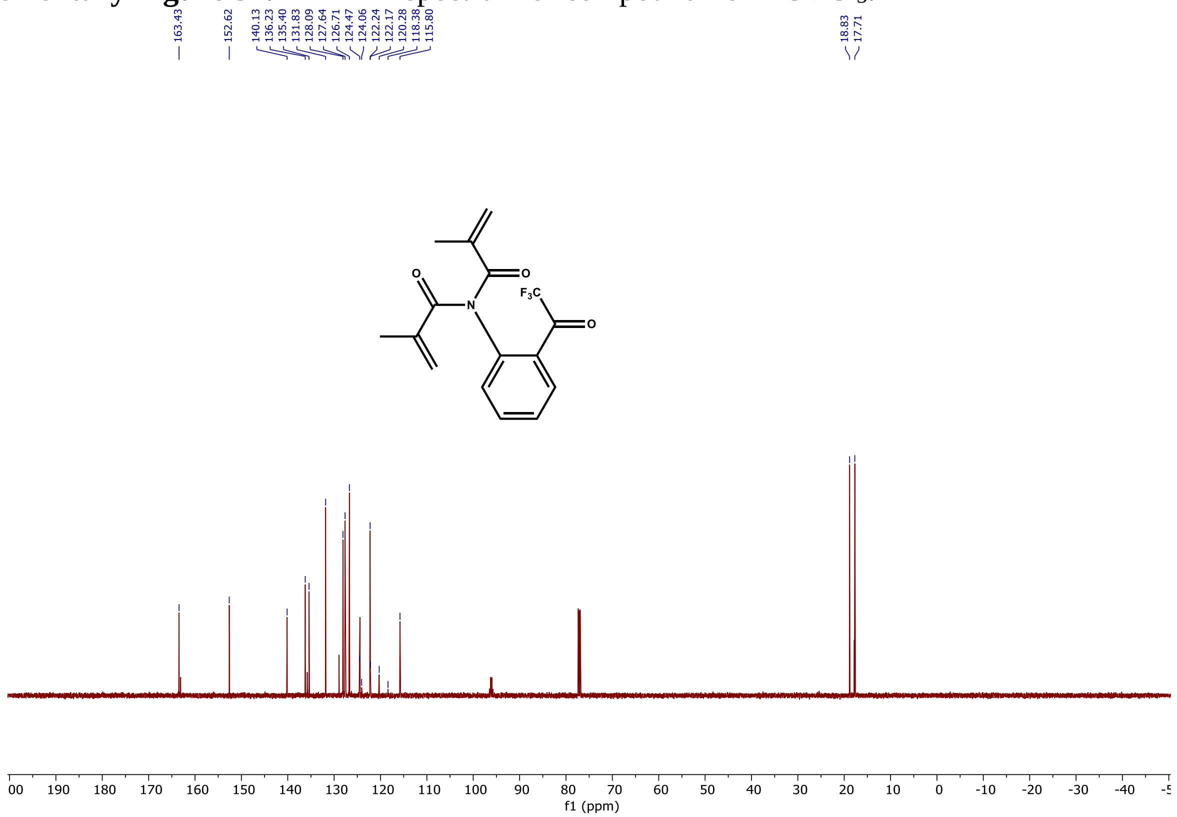

Supplementary Figure 32. ^{13}C NMR spectrum of compound **1b** in $CDCl_3$.

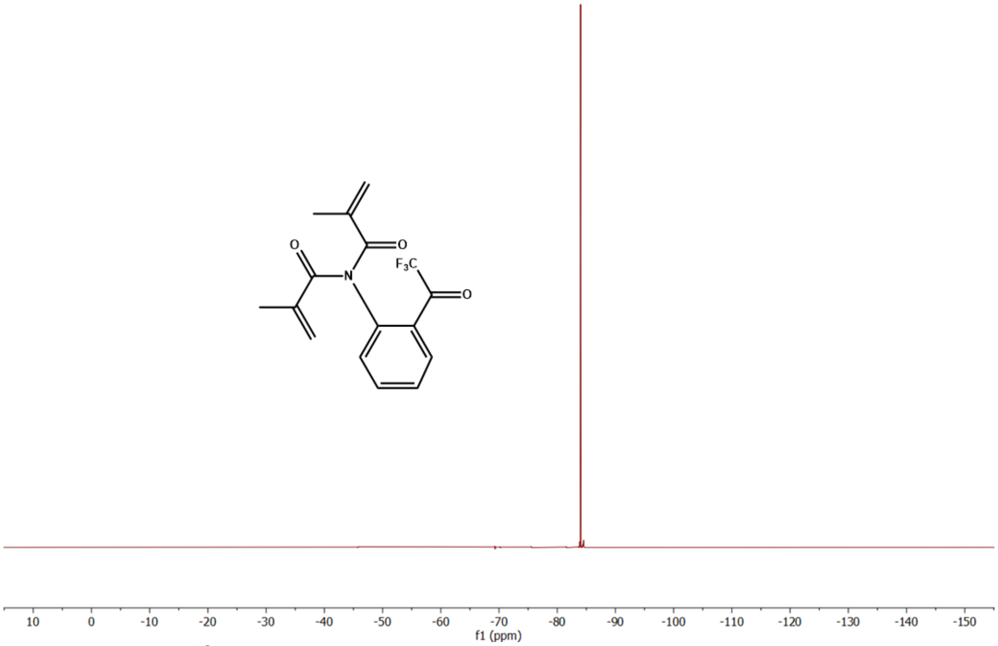

Supplementary Figure 33. ^{19}F NMR spectrum of compound **1b** in $CDCl_3$.

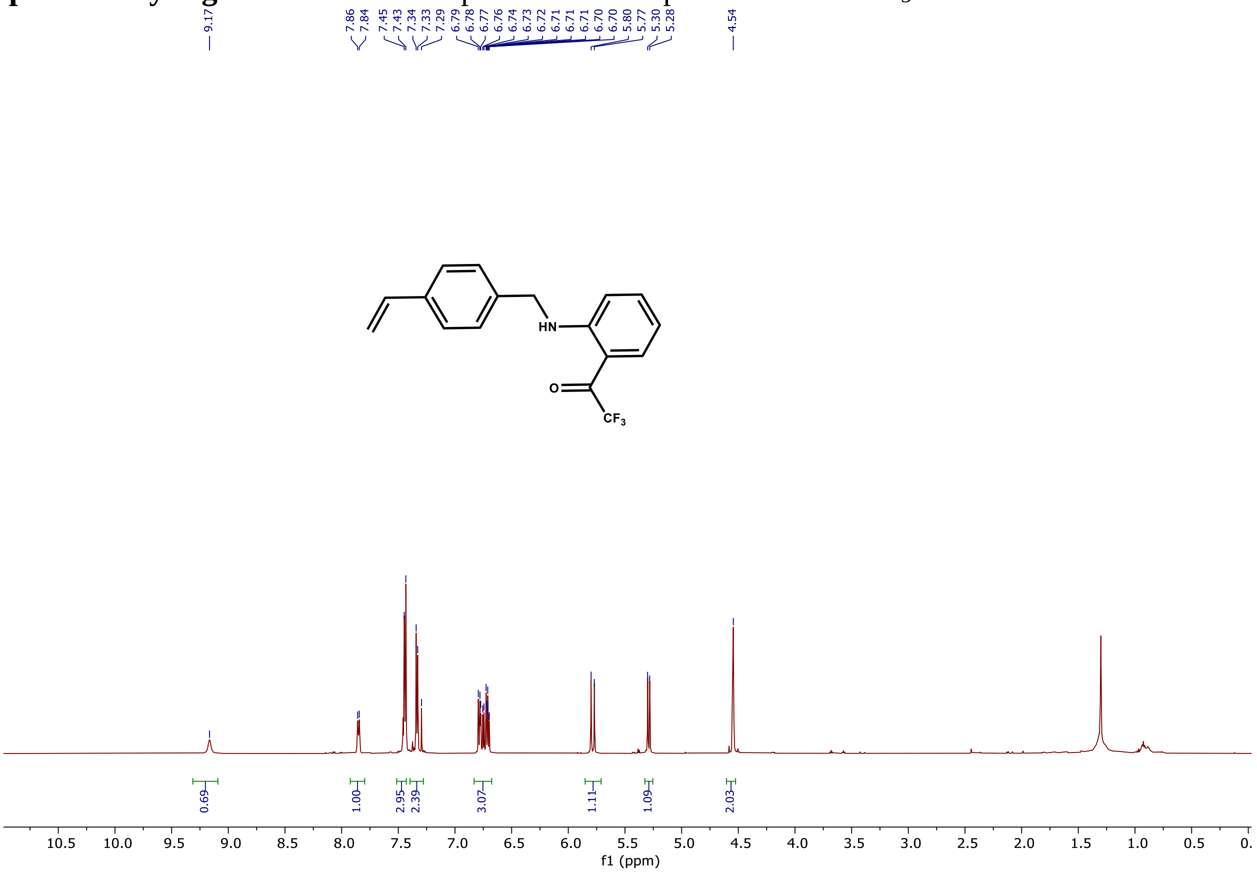

Supplementary Figure 34. ^{1}H NMR spectrum of compound **1c** in $CDCl_3$.

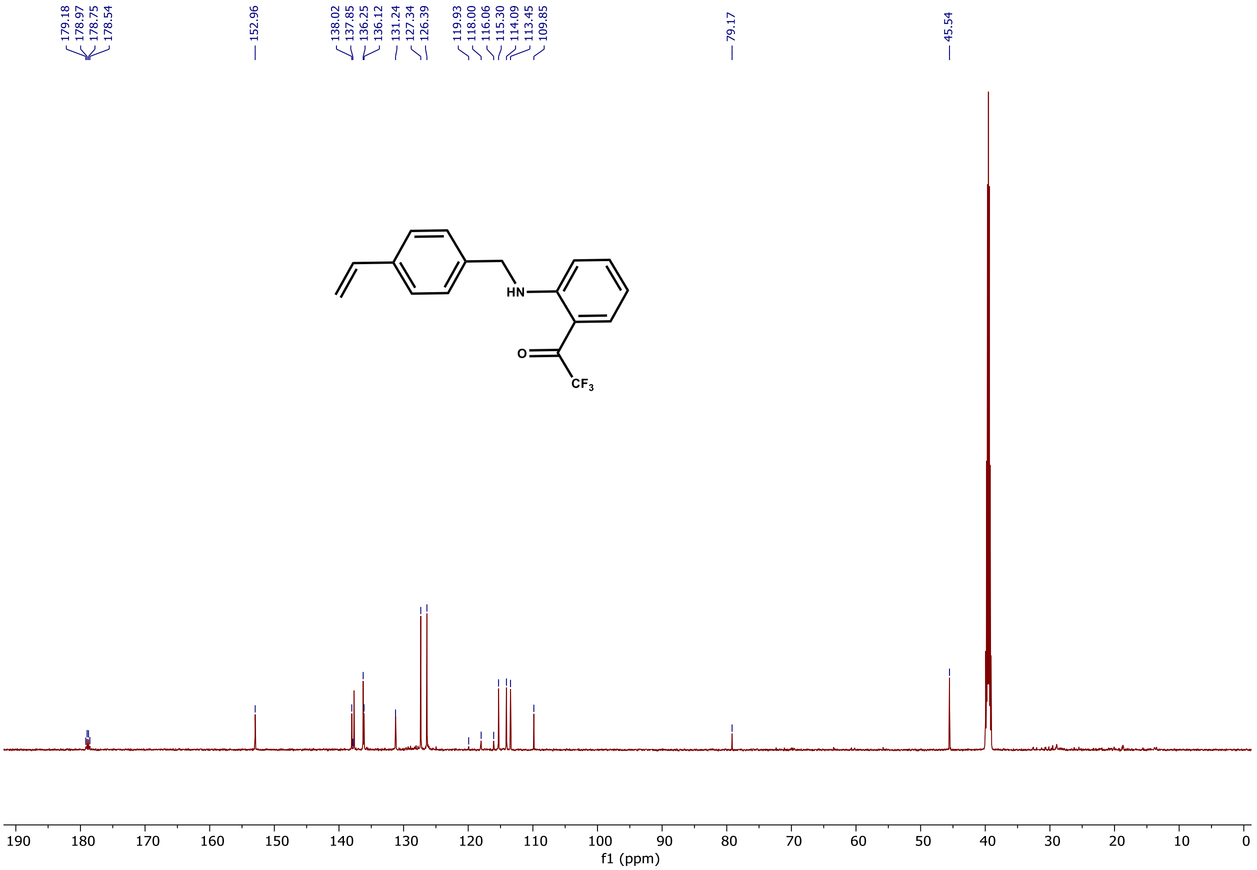

Supplementary Figure 35. ^{13}C NMR spectrum of compound **1c** in DMSO-d$_6$.

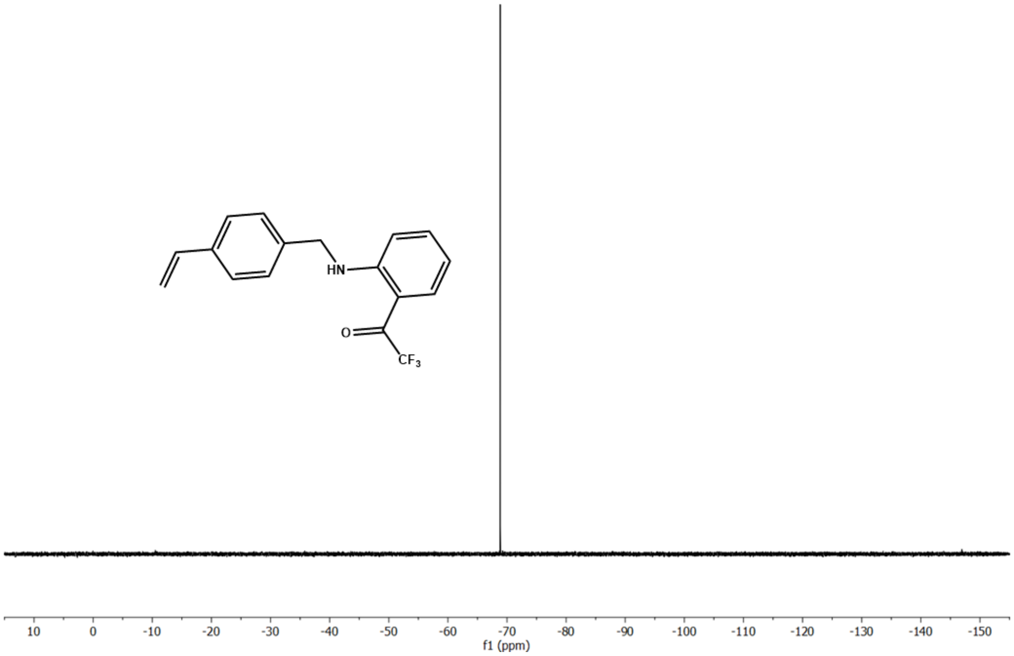

Supplementary Figure 36. ^{19}F NMR spectrum of compound **1c** in in CDCl$_3$.

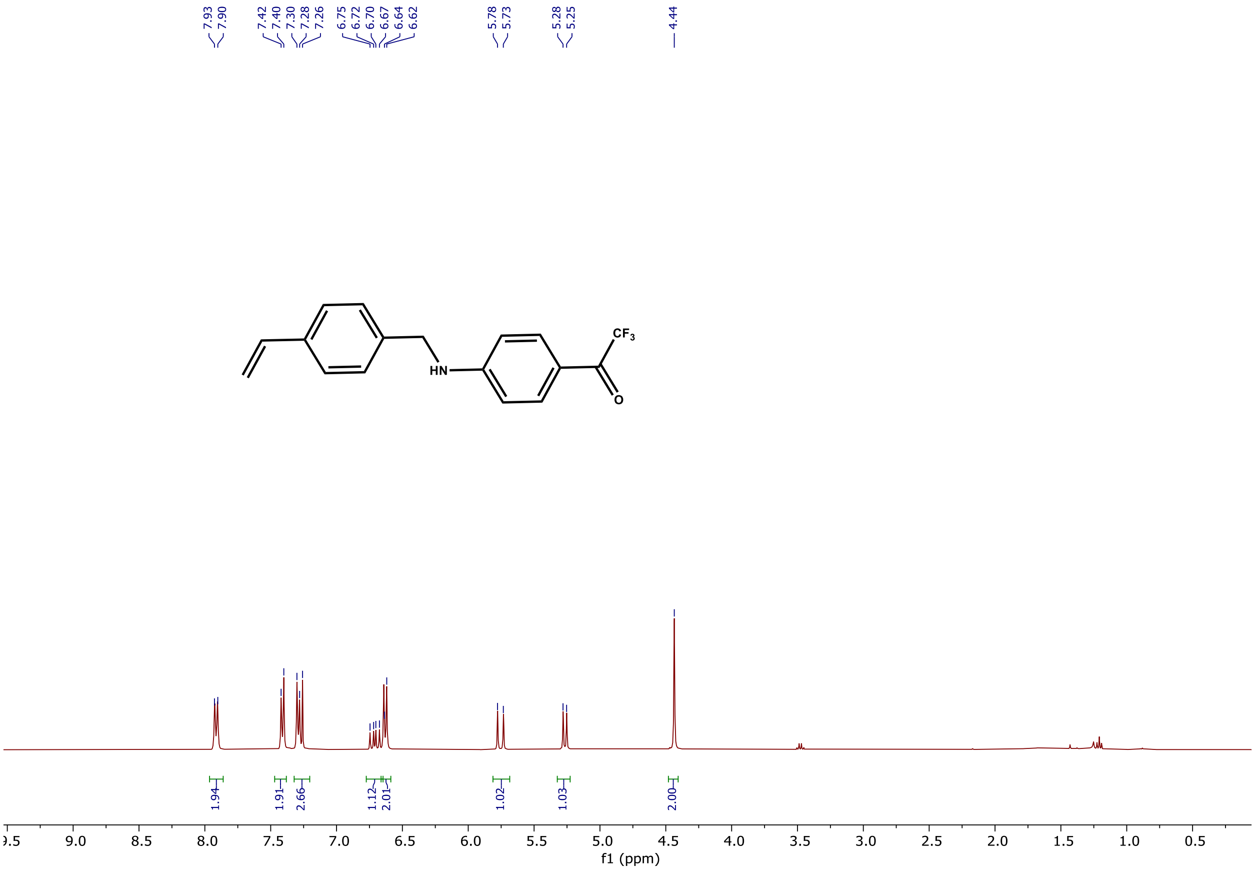

Supplementary Figure 37. ^{1}H NMR spectrum of compound **1d** in $CDCl_3$.

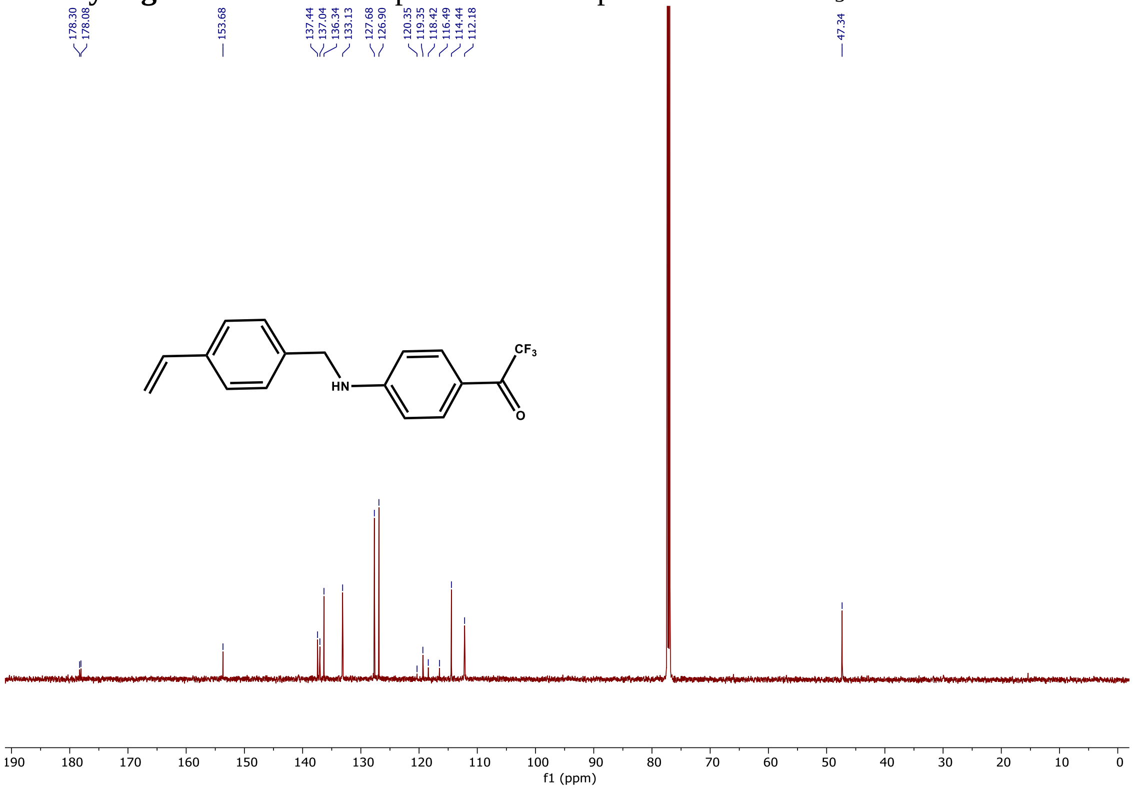

Supplementary Figure 38. ^{13}C NMR spectrum of compound **1d** in $CDCl_3$**.**

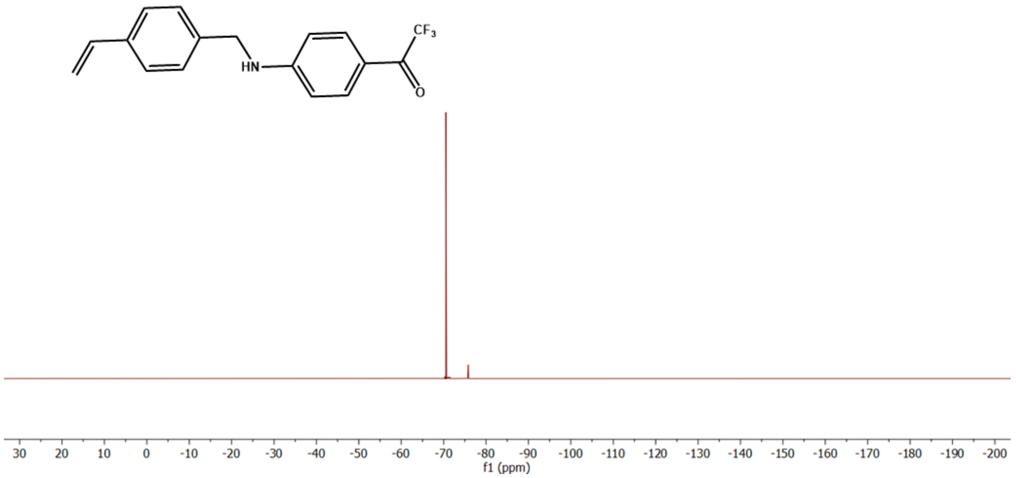

Supplementary Figure 39. ^{19}F NMR spectrum of compound **1d** in $CDCl_3$.

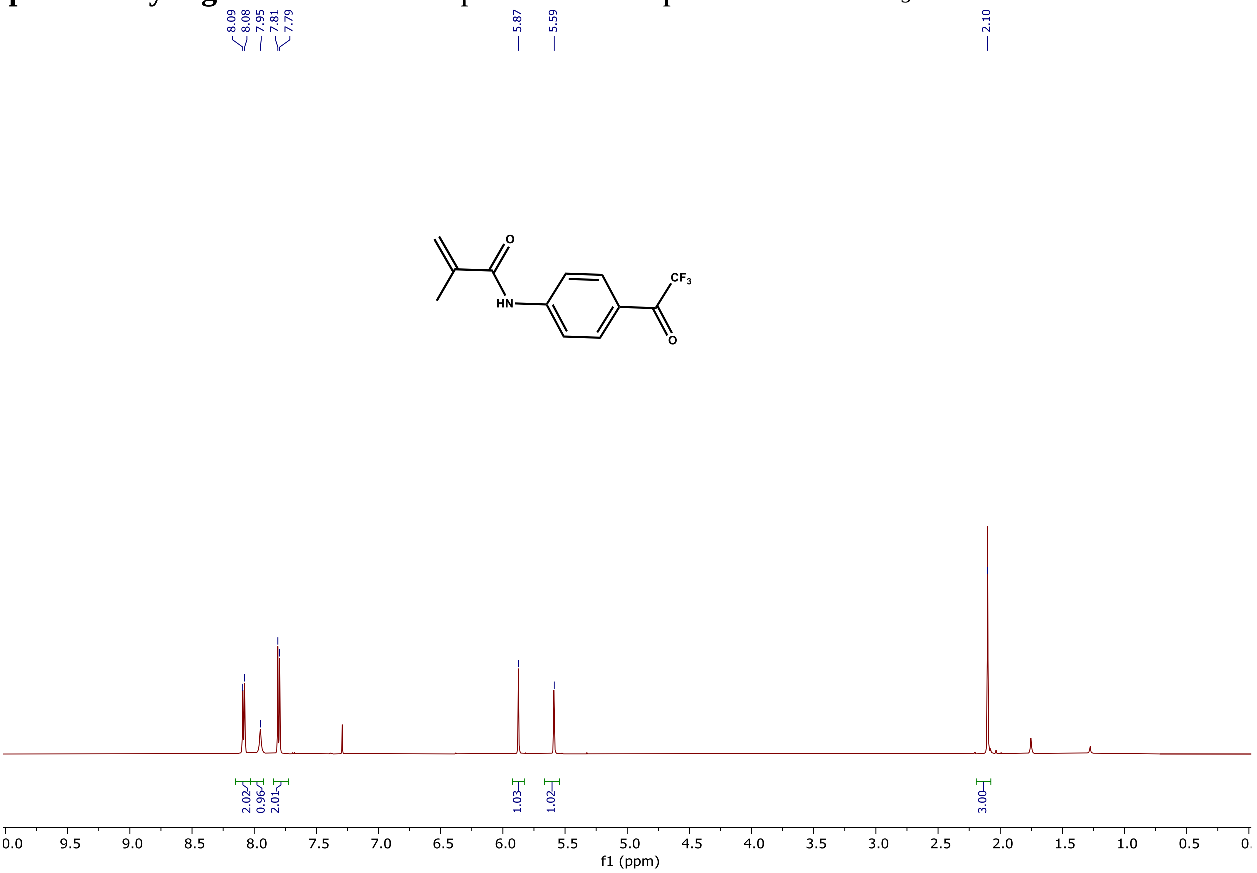

Supplementary Figure 40. ^{1}H NMR spectrum of compound **1e** in $CDCl_3$.

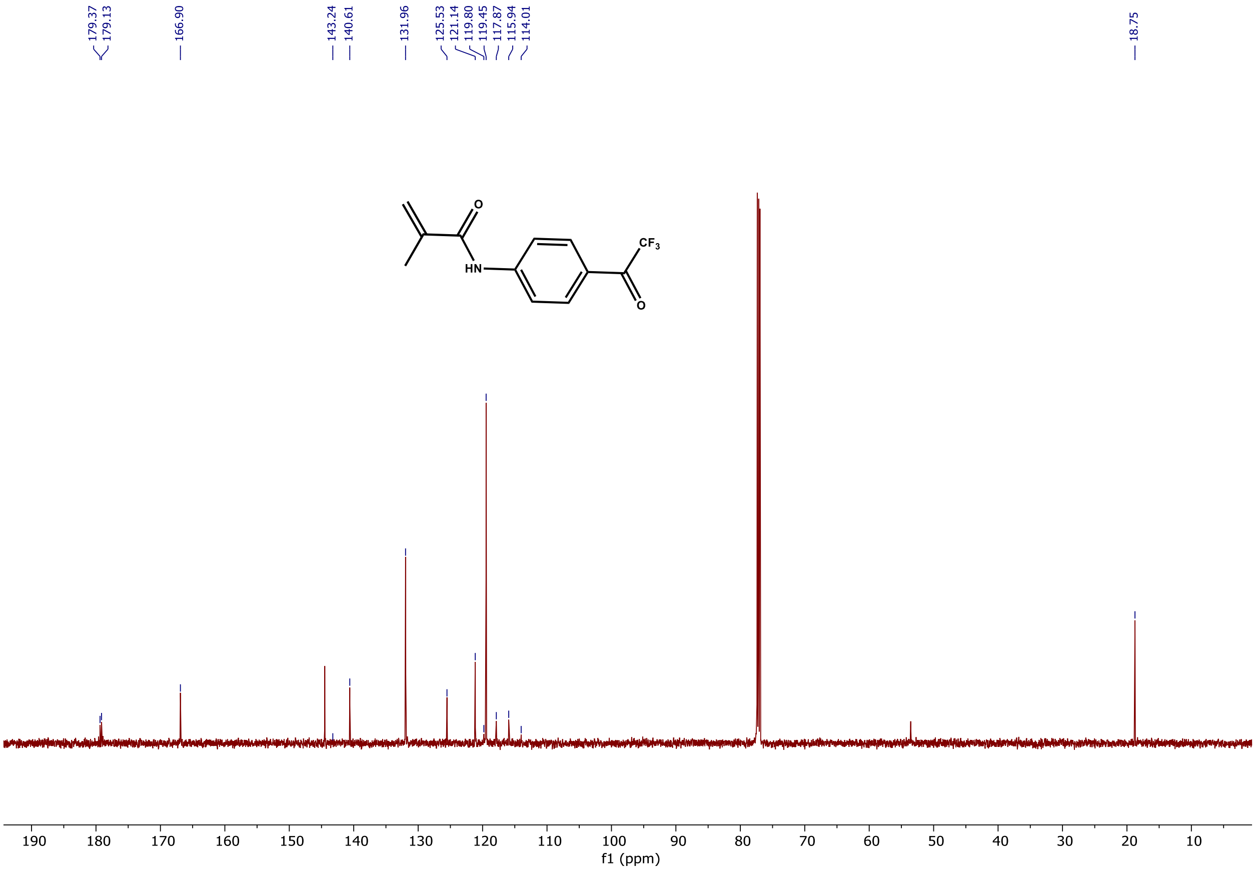

Supplementary Figure 41. ^{13}C NMR spectrum of compound **1e** in $CDCl_3$.

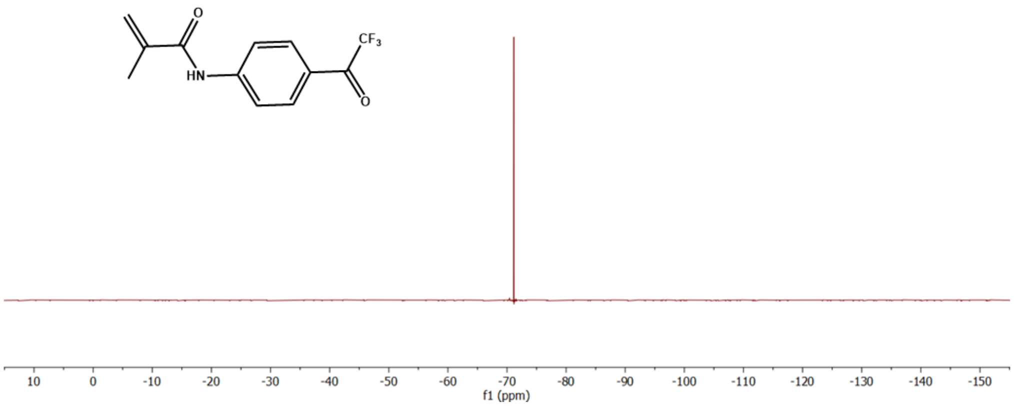

Supplementary Figure 42. ^{19}F NMR spectrum of compound **1e** in $CDCl_3$.

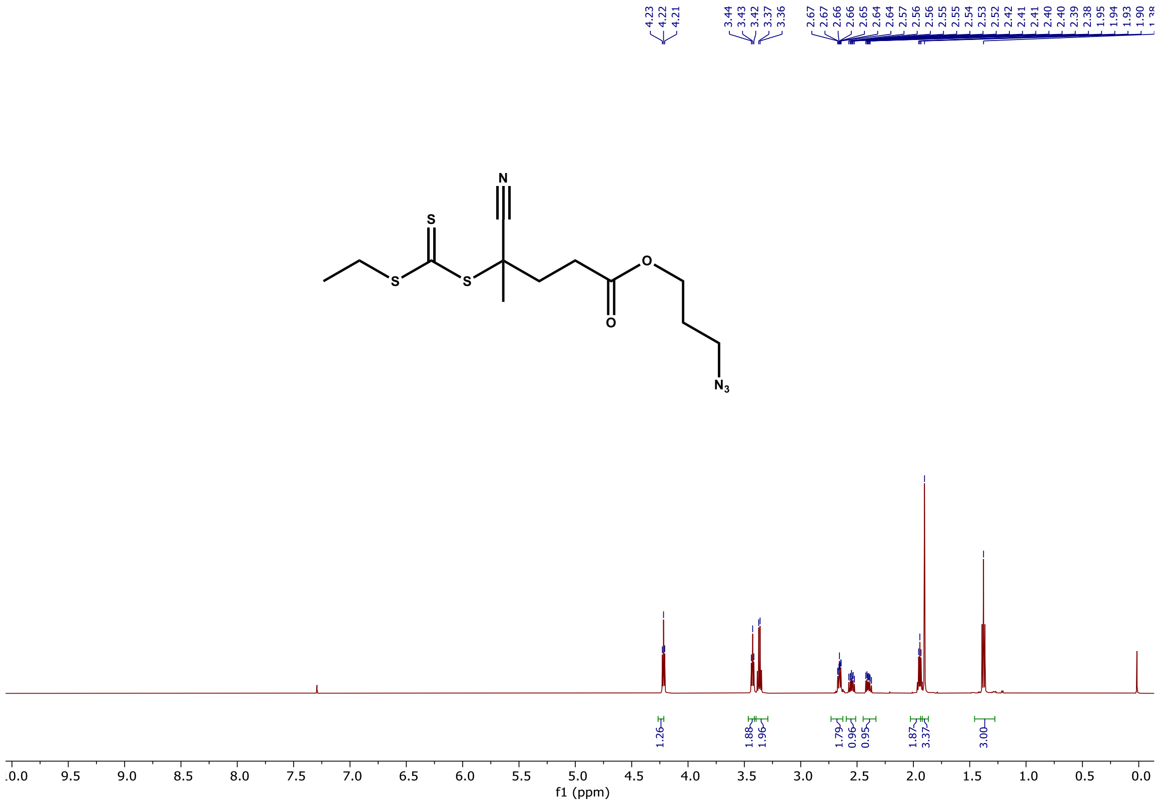

Supplementary Figure 43. ^{1}H NMR spectrum of compound **6** in $CDCl_3$.

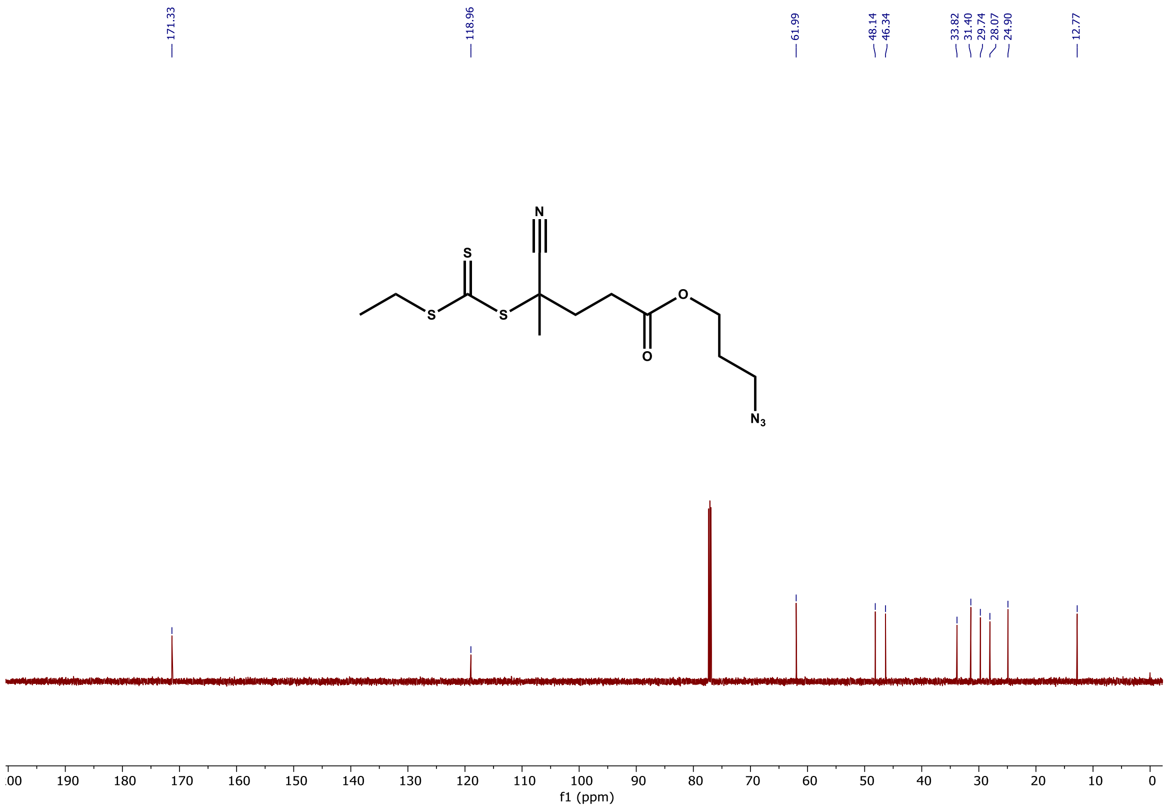

Supplementary Figure 44. ^{13}C NMR spectrum of compound **6** in $CDCl_3$.

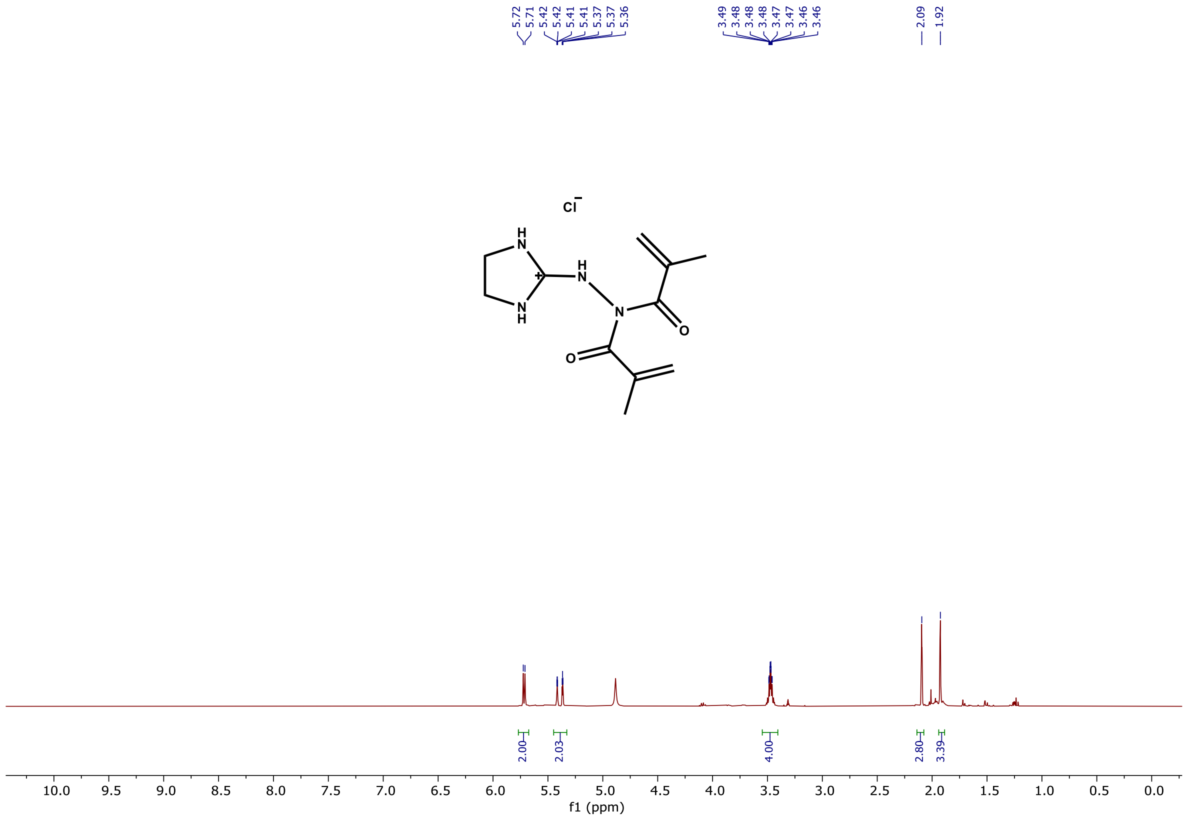

Supplementary Figure 45. ^{1}H NMR spectrum of compound **7** in CD_3OD.

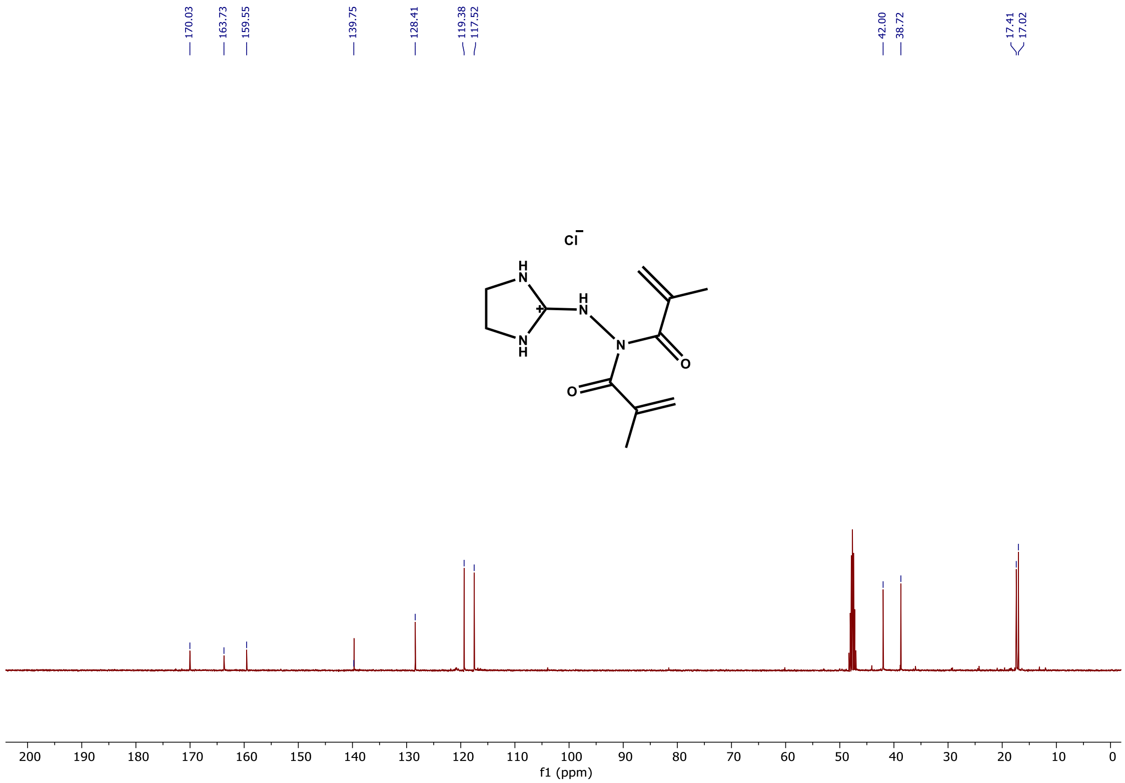

Supplementary Figure 46. ^{13}C NMR spectrum of compound **7** in CD_3OD.

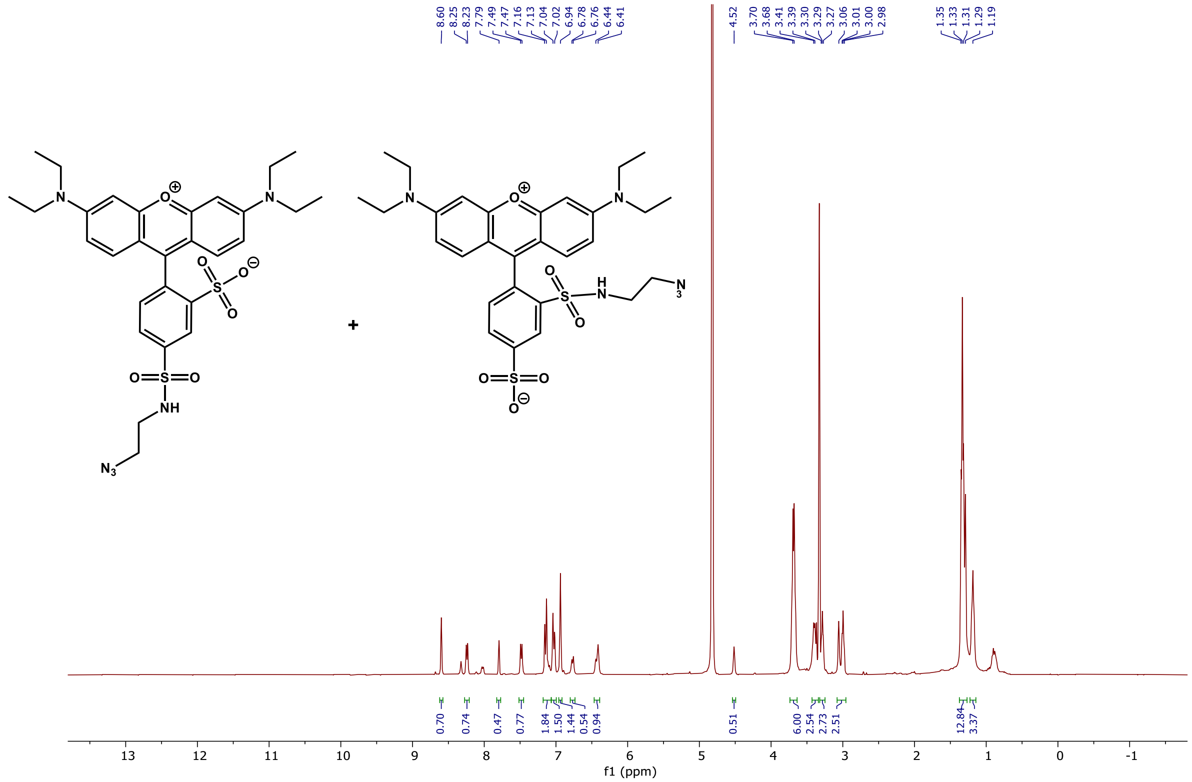

Supplementary Figure 47. ^{1}H NMR spectrum of compound **8** in $CD_3OD/CDCl_3$.

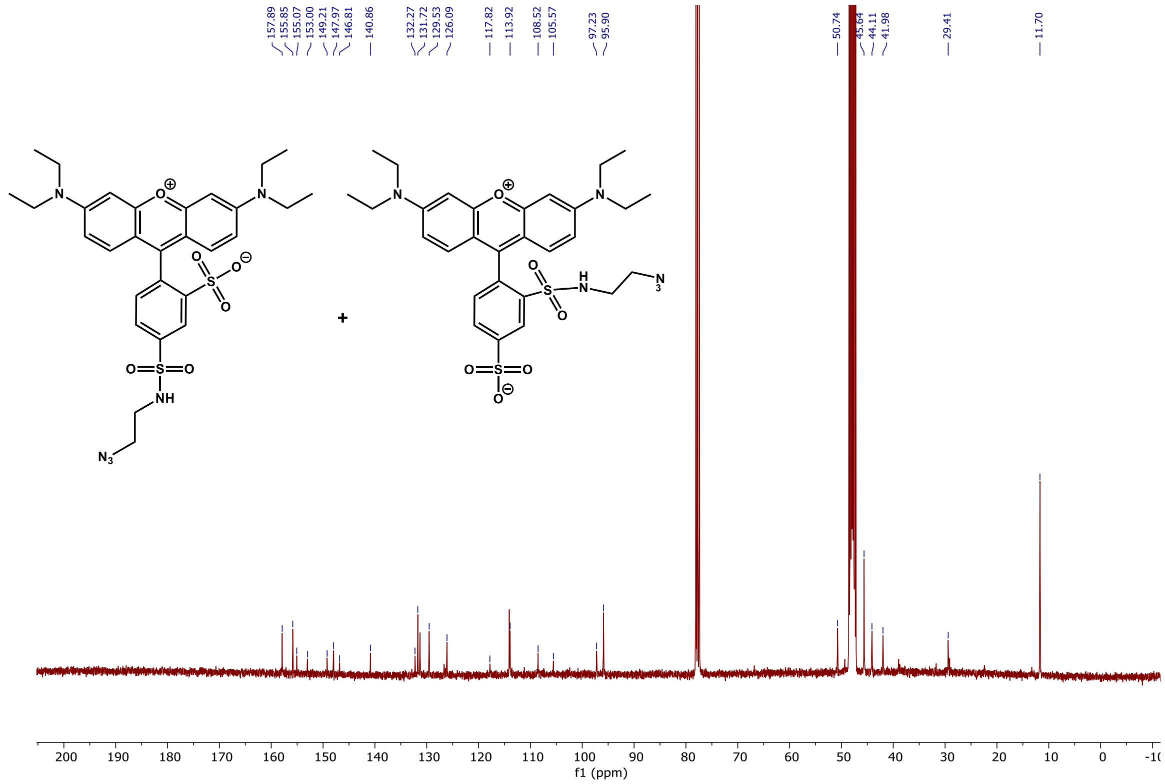

Supplementary Figure 48. ^{13}C NMR spectrum of compound **8** in $CD_3OD/CDCl_3$.

3. Supplementary References

(1) Awino, J. K.; Zhao, Y. Protein-Mimetic, Molecularly Imprinted Nanoparticles for Selective Binding of Bile Salt Derivatives in Water. *J. Am. Chem. Soc.* **2013**, *135*, 12552-12555.

(2) Zhang, S.; Zhao, Y. Facile Synthesis of Multivalent Water-Soluble Organic Nanoparticles via "Surface Clicking" of Alkynylated Surfactant Micelles. *Macromolecules* **2010**, *43*, 4020-4022.

(3) Ryu, E. H.; Zhao, Y. Efficient Synthesis of Water-Soluble Calixarenes Using Click Chemistry. *Org. Lett.* **2005**, *7*, 1035-1037

(4) Prakash,G. K. S; Krishnamurti, R.; Olah, G. A. Y. Protein-Mimetic, Fluoride-induced trifluoromethylation of carbonyl compounds with trifluoromethyltrimethylsilane (TMS-CF_3). A trifluoromethide equivalent. *J. Am. Chem. Soc.* **1989**, *111*, 393-395.

(5) Cheng, H.; Pei, Y.; Leng, F.; Li, J.; Liang, A.; Zou, D.; Wu, Y.; Wu, Y. Highly efficient synthesis of aryl and heteroaryl trifluoromethyl ketones via o-iodobenzoic acid (IBX). *Tetrahedron Lett.* **2013**, *54*, 4483-4486.

(6) Sumerlin, B. S.; Tsarevsky, N. V.; Louche, G.; Lee, R.Y.; Matyjaszewski, K. Highly Efficient "Click" Functionalization of Poly(3-azidopropyl methacrylate) Prepared by ATRP, *Macromolecules* **2005**, *38*, 7540–7545.

(7) Erickson, H. P. Size and Shape of Protein Molecules at the Nanometer Level Determined by Sedimentation, Gel Filtration, and Electron Microscopy. *Biol. Proced. Online* **2009**, *11*, 32.

(8) Das, B.; Lou-Franco, J.; Gilbride, B.; Ellis, M. G.; D. L.; Stewart, Grant, I. R.; Balasubramanian, P.; and Cao, C. Peroxidase-Mimicking Activity of Biogenic Gold Nanoparticles Produced from Prunus nepalensis Fruit Extract: Characterizations and Application for the Detection of Mycobacterium bovis. *ACS Appl. Bio Mater.* **2022**, *5*, 2712-2725.

(9) Cao, G.; Jiang, X.; Zhang, H.; Croley, T. R.; Yin, J. Mimicking horseradish peroxidase and oxidase using ruthenium nanomaterials. *RSC Adv.* **2017**, *7*, 52210-52217.
