# Supplementary material for: Cell-penetrating protein-recognizing polymeric nanoparticles through dynamic covalent chemistry and double imprinting
Source: Nat Commun. 2024 May 3;15:3731. doi: 10.1038/s41467-024-48131-5 (PMC11068882; doi:10.1038/s41467-024-48131-5)
Supplement: Supplementary file 2 — Reporting Summary [file 41467_2024_48131_MOESM2_ESM.pdf]

nature portfolio

Corresponding author(s): Yan Zhao

Last updated by author(s): 03/25/2024

Reporting Summary

Nature Portfolio wishes to improve the reproducibility of the work that we publish. This form provides structure for consistency and transparency in reporting. For further information on Nature Portfolio policies, see our Editorial Policies and the Editorial Policy Checklist.

Statistics

For all statistical analyses, confirm that the following items are present in the figure legend, table legend, main text, or Methods section.

n/a	Confirmed	
☐	☒	The exact sample size (*n*) for each experimental group/condition, given as a discrete number and unit of measurement
☐	☒	A statement on whether measurements were taken from distinct samples or whether the same sample was measured repeatedly
☐	☒	The statistical test(s) used AND whether they are one- or two-sided<br>*Only common tests should be described solely by name; describe more complex techniques in the Methods section.*
☒	☐	A description of all covariates tested
☐	☒	A description of any assumptions or corrections, such as tests of normality and adjustment for multiple comparisons
☐	☒	A full description of the statistical parameters including central tendency (e.g. means) or other basic estimates (e.g. regression coefficient) AND variation (e.g. standard deviation) or associated estimates of uncertainty (e.g. confidence intervals)
☐	☒	For null hypothesis testing, the test statistic (e.g. *F*, *t*, *r*) with confidence intervals, effect sizes, degrees of freedom and *P* value noted<br>*Give P values as exact values whenever suitable.*
☒	☐	For Bayesian analysis, information on the choice of priors and Markov chain Monte Carlo settings
☐	☒	For hierarchical and complex designs, identification of the appropriate level for tests and full reporting of outcomes
☒	☐	Estimates of effect sizes (e.g. Cohen's *d*, Pearson's *r*), indicating how they were calculated

Our web collection on statistics for biologists contains articles on many of the points above.

Software and code

Policy information about availability of computer code

Data collection	NMR spectra were recorded on a Bruker DRX-400, a Bruker AV III 600, or a Varian VXR-400 spectrometer. High resolution mass spectra (HRMS) were recorded on Agilent QTOF 6540 mass spectrometer with a QTOF detector. Dynamic light scattering (DLS) was recorded at 25 °C on a Malvern Zetasizer Nano ZS instrument. Isothermal titration calorimetry (ITC) was performed using a MicroCal VP-ITC Microcalorimeter. TEM was analyzed on a 200kV JEOL 2100 selectron microscope.
Data analysis	ITC data were analyzed with Origin 7 software and VPViewer2000; Fluorescent images were analyzed with ZEN microscopy software; Analysis of apoptosis data and contrasts were made in R 4.3.1.

For manuscripts utilizing custom algorithms or software that are central to the research but not yet described in published literature, software must be made available to editors and reviewers. We strongly encourage code deposition in a community repository (e.g. GitHub). See the Nature Portfolio guidelines for submitting code & software for further information.

Data

Policy information about availability of data

All manuscripts must include a data availability statement. This statement should provide the following information, where applicable:

- Accession codes, unique identifiers, or web links for publicly available datasets
- A description of any restrictions on data availability
- For clinical datasets or third party data, please ensure that the statement adheres to our policy

All the data supporting the findings of this study are available within the article and Supplementary Information, or available from the corresponding author on

request. Protein structures are from Protein Data Bank (PDB ID: 5IY5 and 1w4w).

Research involving human participants, their data, or biological material

Policy information about studies with human participants or human data. See also policy information about sex, gender (identity/presentation), and sexual orientation and race, ethnicity and racism.

Reporting on sex and gender	N/A
Reporting on race, ethnicity, or other socially relevant groupings	N/A
Population characteristics	N/A
Recruitment	N/A
Ethics oversight	N/A

Note that full information on the approval of the study protocol must also be provided in the manuscript.

Field-specific reporting

Please select the one below that is the best fit for your research. If you are not sure, read the appropriate sections before making your selection.

☒ Life sciences ☐ Behavioural & social sciences ☐ Ecological, evolutionary & environmental sciences

For a reference copy of the document with all sections, see nature.com/documents/nr-reporting-summary-flat.pdf

Life sciences study design

All studies must disclose on these points even when the disclosure is negative.

Sample size	No power analysis was conducted before the experiments. Significant tests for each experiment show strong evidence of treatment effects (see Supplementary Table 12).
Data exclusions	No data were excluded from analysis
Replication	The experiment was designed as Randomized Complete Block Design (RCBD) with 3 blocks (replications): different batches of cells served as blocking factors.
Randomization	Treatment and dosage combinations were randomized within each block.
Blinding	Not applicable. All experimental units were measured 3 times and averaged to represent each experimental unit.

Reporting for specific materials, systems and methods

We require information from authors about some types of materials, experimental systems and methods used in many studies. Here, indicate whether each material, system or method listed is relevant to your study. If you are not sure if a list item applies to your research, read the appropriate section before selecting a response.

Materials & experimental systems

n/a	Involved in the study	
☒	☐	Antibodies
☐	☒	Eukaryotic cell lines
☒	☐	Palaeontology and archaeology
☒	☐	Animals and other organisms
☒	☐	Clinical data
☒	☐	Dual use research of concern
☒	☐	Plants

Methods

n/a	Involved in the study	
☒	☐	ChIP-seq
☐	☒	Flow cytometry
☒	☐	MRI-based neuroimaging

Eukaryotic cell lines

Policy information about cell lines and Sex and Gender in Research

Cell line source(s)	American type culture collection (ATCC HTB-26)
Authentication	Cell lines used were not authenticated
Mycoplasma contamination	Cell lines were not tested for mycoplasma
Commonly misidentified lines (See ICLAC register)	No commonly misidentified cell lines were used

Flow Cytometry

Plots

Confirm that:

- [x] The axis labels state the marker and fluorochrome used (e.g. CD4-FITC).
- [x] The axis scales are clearly visible. Include numbers along axes only for bottom left plot of group (a 'group' is an analysis of identical markers).
- [x] All plots are contour plots with outliers or pseudocolor plots.
- [x] A numerical value for number of cells or percentage (with statistics) is provided.

Methodology

Sample preparation	Sample preparation is described in the Supplementary Methods. 1 ×105 MDA-MB-231 cells were seeded onto 12-well plates per well and maintained in a humid 5% CO2-containing incubator. The cells were treated with 5–25 µM NPB(Cytc)* and incubated for 1 h. They were washed with a cold phosphate-buffered saline (PBS) and harvested with 1 × trypsin. The washed cells were centrifugated and resuspended in the FACS-binding buffer to a density of ~1 × 106 cells/mL. Finally, The nanoparticle-stained cells were analyzed by flow cytometry .
Instrument	The FACSAria III
Software	BD FACSDiva 8.0.1
Cell population abundance	All flow cytometry data regard only sample analysis.
Gating strategy	Gating strategy is provided in Supplementary Figure S19, S20, S23 and S 24

- [x] Tick this box to confirm that a figure exemplifying the gating strategy is provided in the Supplementary Information.
